# New efficient synthesis of polysubstituted 3,4-dihydroquinazolines and 4*H*-3,1-benzothiazines through a Passerini/Staudinger/aza-Wittig/addition/nucleophilic substitution sequence

**DOI:** 10.3762/bjoc.18.32

**Published:** 2022-03-04

**Authors:** Long Zhao, Mao-Lin Yang, Min Liu, Ming-Wu Ding

**Affiliations:** 1 Key Laboratory of Pesticide & Chemical Biology of Ministry of Education, Hubei International Scientific and Technological Cooperation Base of Pesticide and Green Synthesis, Central China Normal University, Wuhan, 430079, P. R. China

**Keywords:** aza-Wittig reaction, 3,4-dihydroquinazoline, 4*H*-3,1-benzothiazine, nucleophilic substitution, Passerini reaction, Staudinger reaction

## Abstract

A new efficient synthesis of polysubstituted 3,4-dihydroquinazolines and 4*H*-3,1-benzothiazines via sequential Passerini/Staudinger/aza-Wittig/addition/nucleophilic substitution reaction has been developed. The three-component Passerini reactions of 2-azidobenzaldehydes **1**, benzoic acid (**2**), and isocyanides **3** produced the azide intermediates **4**, which were treated sequentially with triphenylphosphine, isocyanates (or CS_2_), and secondary amines to give polysubstituted 3,4-dihydroquinazolines **8** and 4*H*-3,1-benzothiazines **11** in good overall yields through consecutive Passerini/Staudinger/aza-Wittig/addition/nucleophilic substitution reactions.

## Introduction

**Figure 1:**
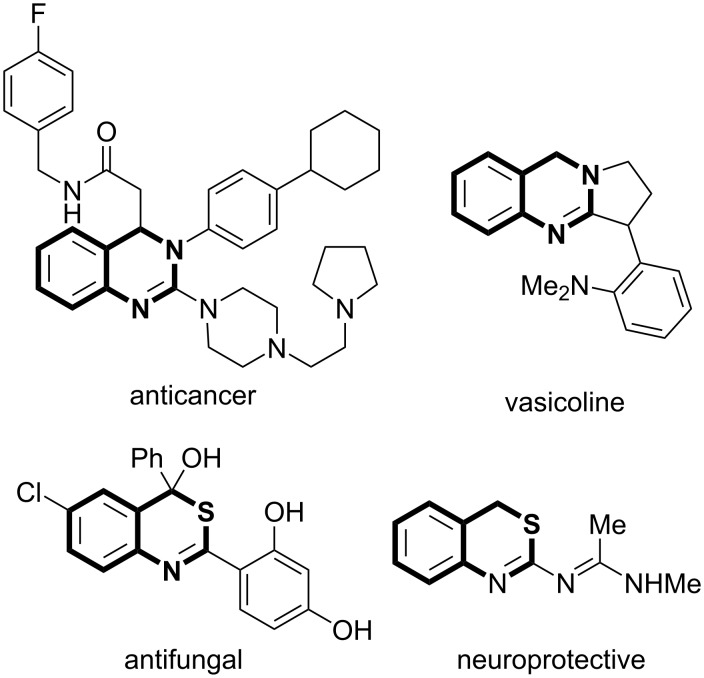
Some bioactive 3,4-dihydroquinazolines and 4*H*-3,1-benzothiazines.

**Scheme 1:**
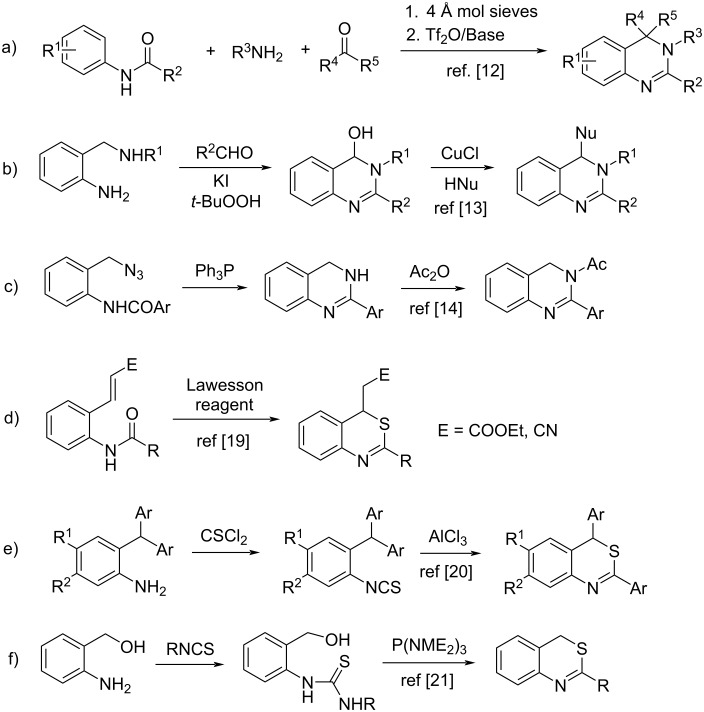
Representative preperation of 3,4-dihydroquinazolines and 4*H*-3,1-benzothiazines.

The chemistry of 3,4-dihydroquinazolines and 4*H*-3,1-benzothiazines is of constant interest owing to the occurrence of these ring systems in various biologically important compounds (Figure 1). A number of 3,4-dihydroquinazolines were found to show remarkable anticancer [1], antiviral [2], antidepressant [3], antifungal [4], selective somatostatin 2 (ss2) agonistical [5], β-site amyloid precursor protein cleaving enzyme 1 (BACE-1) inhibitive [6], and cholinesterase enzyme inhibitive activities [7]. The 3,4-dihydroquinazoline skeleton also exists in some natural products such as vasicine and vasicoline [8]. Some 4*H*-3,1-benzothiazine derivatives have also received attention due to their good biological activities, including anticancer [9], neuroprotective [10], antiproliferative and antifungal activities [11]. Due to the significant bioactive properties of the 3,4-dihydroquinazoline and 4*H*-3,1-benzothiazine moieties, many preparation procedures have appeared in the literature for the synthesis of their derivatives [12–22]. For example (Scheme 1), a one-pot Tf_2_O-mediated assembly of amides, amines, and ketones provided 3,4-dihydroquinazolines in good yields via successive triflic anhydride-mediated amide dehydration, ketimine addition, and Pictet–Spengler-like cyclization processes [12]. Some 4-substituted 3,4-dihydroquinazolines were prepared by copper-catalyzed oxidative cross coupling of hydroxy intermediates with various nucleophiles [13]. Other 3,4-dihydroquinazolines were also obtained efficiently by intramolecular aza-Wittig reactions [14]. Some 4*H*-3,1-benzothiazines were prepared by intramolecular thia-Michael addition with broad reaction scopes [19]. The rearrangement of 2-isothiocyano triarylmethanes in the presence of AlCl_3_ were also used for the synthesis 2,4-diaryl-4*H*-3,1-benzothiazines through aromatic ring transfer [20]. A facile protocol towards the synthesis of 4*H*-3,1-benzothiazines was established by using a P(NMe_2_)_3_-mediated C–N/C–S bond formation reaction of 2-aminobenzyl alcohol with isothiocyanates under aerobic conditions [21]. Despite of the above achievements, the development of new efficient methods for the synthesis of polysubstituted 3,4-dihydroquinazolines and 4*H*-3,1-benzothiazines under mild reaction conditions is still of high demand in the discovery of biologically active compounds.

The Passerini reaction is an isocyanide-based multicomponent reaction, which has been used in preparing various α-acyloxy adducts starting from aldehydes, a carboxylic acid, and a isonitrile as the three components [23]. The sequences of Passerini reactions, followed by post-condensation reactions, constitute useful synthetic methods in the preparation of structurally diverse heterocyclic compounds [24–29]. The aza-Wittig reaction has also been utilized widely in preparation of various heterocycles under mild neutral conditions [30–32]. Recently we have reported the synthesis of 3*H*-2-benzoxepin-1-ones, 4*H*-3,1-benzoxazines and oxazoles by combination of a Passerini with an intramolecular aza-Wittig reaction [33–35]. Continuing our interest in the synthesis of *N*-heterocycles via the aza-Wittig reaction and multicomponent reactions [36–38], we wish to report herein a facile synthesis of polysubstituted 3,4-dihydroquinazolines and 4*H*-3,1-benzothiazines via sequential Passerini/Staudinger/aza-Wittig/addition/nucleophilic substitution reactions. Compared with the synthetic method to 4*H*-3,1-benzothiazines in Scheme 1f, we provide another new sequential synthetic route to 4*H*-3,1-benzothiazines, especially for *N*,*N*-disubstituted 2-amino-4*H*-3,1-benzothiazines.

## Results and Discussion

**Scheme 2:**
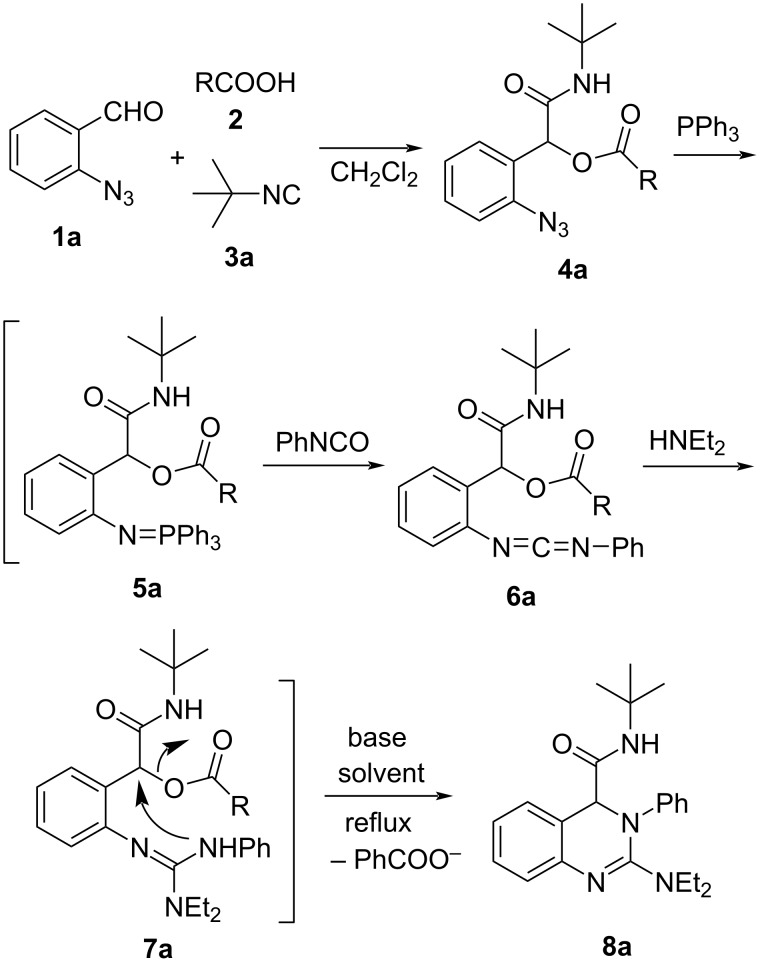
Preparation of 3,4-dihydroquinazoline **8a**.

We initially selected 2-azidobenzaldehyde (**1a**), benzoic acid (**2a**) and *tert*-butyl isocyanide (**3a**) as the reactants (Scheme 2). When a mixture of **1a**, **2a**, and **3a** in CH_2_Cl_2_ was stirred at room temperature for 48 h, the three-component Passerini reaction was carried out smoothly and the azide **4a** (R = Ph) was finally obtained in 87% yield. Compound **4a** was then allowed to react with triphenylphosphine in CH_2_Cl_2_ at room temperature for 2 h to produce the iminophosphorane **5a** by Staudinger reaction. Aza-Wittig reaction of **5a** with phenyl isocyanate generated carbodiimide **6a**, which was then treated with diethylamine to form the guanidine intermediate **7a**. In the presence of K_2_CO_3_ in CH_3_CN at refluxing temperature, the 3,4-dihydroquinazoline **8a** was finally obtained in 84% yield (Table 1, entry 1, the overall yield is 73%) by intramolecular nucleophilic substitution. The reaction conditions for the transformation of guanidine intermediate **7a** into 3,4-dihydroquinazoline **8a** was then optimized (Table 1). As K_2_CO_3_ in different solvents (DMF, CH_2_Cl_2_ and toluene) were used, 0–72% yields of the product **8a** were obtained (Table 1, entries 2–4). Utilizing a stronger base (NaOH and EtONa) resulted in a dark solution and no product was received (entries 5 and 6) owning to side reactions under the stronger base conditions. No product **8a** was obtained when NEt_3_ in CH_3_CN was used (Table 1, entry 7) probably due to the weaker basic conditions. The effect of different R groups on the reaction yield was also investigated. With R = methyl, no product **8a** was obtained in the presence of K_2_CO_3_/CH_3_CN probably due to the lower reactivity of the -OAc leaving group. In case when R was a 4-NO_2_C_6_H_4_ group, 86% yield of the product **8a** was obtained, however, in this case the Passerini product **4a** (R = 4-NO_2_C_6_H_4_) was obtained only in 62% yield and the overall yield of product **8a** was 53%. Therefore, the reaction conditions of entry 1 in Table 1 were optimal for the above transformation.

**Table 1:** Optimization of the reaction conditions for the preparation of compound **8a**.

entry	R	Conditions	Yield (%)

1	Ph	K_2_CO_3_/CH_3_CN	84
2	Ph	K_2_CO_3_/DMF	72
3	Ph	K_2_CO_3_/CH_2_Cl_2_	0
4	Ph	K_2_CO_3_/toluene	41
5	Ph	NaOH/CH_3_CN	0
6	Ph	NaOEt/EtOH	0
7	Ph	NEt_3_/CH_3_CN	0
8	Me	K_2_CO_3_/CH_3_CN	0
9	4-NO_2_C_6_H_4_	K_2_CO_3_/CH_3_CN	86

**Scheme 3:**
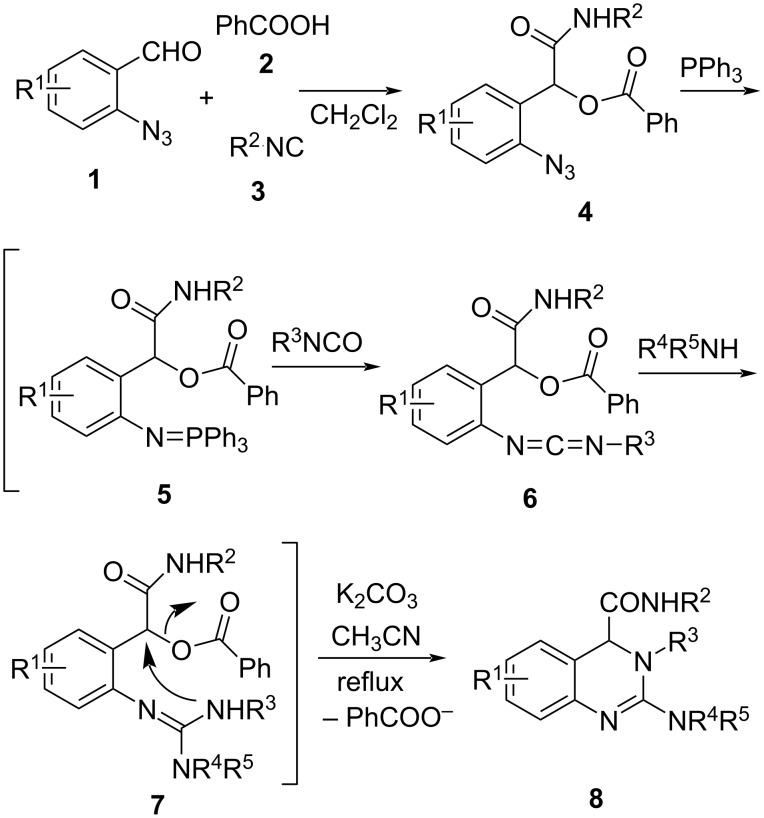
Preparation of 3,4-dihydroquinazolines **8**.

The optimal reaction conditions were then utilized for the sequential reactions of different 2-azidobenzaldehydes **1**, benzoic acid (**2a**), isocyanides **3**, isocyanates and secondary amines. Most of the reactions took place smoothly to give the corresponding 3,4-dihydroquinazolines **8** in good yields (Scheme 3 and Table 2). Various isocyanates and secondary amines can be used in the above one-pot cyclization to prepare 3,4-dihydroquinazolines **8**. As indicated in Table 2, when aromatic isocyanates (Table 2, compounds **8a–l**, R^3^ = Ph, 4-ClC_6_H_4_, 3-MeC_6_H_4_, 4-MeC_6_H_4_ and 4-CF_3_OC_6_H_4_) were used, good yields (69–86%) of the products were obtained, whereas moderate yields (54–57%) were obtained when the more steric secondary amines were utilized (Table 2, compound **8m** and **8n**, NR^4^R^5^ = N(Cy)_2_, N(iPr)_2_). In cases when aliphatic isocyanates (compounds **8o–q**, R^3^ = *n*-Bu, cyclohexyl and PhCH_2_) were used, 65–74% yields of the products were obtained. Even as the steric *tert*-butyl isocyanate was applied, the 3,4-dihydroquinazoline **8r** was obtained in 42% yield, but when diphenylamine was used, no product was obtained (compounds **8s**, NR^4^R^5^ = NPh_2_).

**Table 2:** Yields of 3,4-dihydroquinazolines **8**.

	R^1^	R^2^	R^3^	NR^4^R^5^	Yield^a^ (%)

**8a**	H	*t*-Bu	Ph	NEt_2_	84
**8b**	H	*t*-Bu	4-ClC_6_H_4_	NEt_2_	80
**8c**	H	*t*-Bu	3-MeC_6_H_4_	NEt_2_	76
**8d**	H	*t*-Bu	4-MeC_6_H_4_	NEt_2_	79
**8e**	H	*t*-Bu	Ph	morpholin-4-yl	72
**8f**	H	*t*-Bu	4-MeC_6_H_4_	NPr_2_	85
**8g**	H	*t*-Bu	4-MeC_6_H_4_	NBu_2_	69
**8h**	H	Cy^b^	4-MeC_6_H_4_	NEt_2_	71
**8i**	H	Cy^b^	Ph	NEt_2_	86
**8j**	H	Cy^b^	4-ClC_6_H_4_	NEt_2_	78
**8k**	H	Cy^b^	4-CF_3_OC_6_H_4_	NEt_2_	80
**8l**	H	*t*-Bu	4-MeC_6_H_4_	morpholin-4-yl	70
**8m**	H	*t*-Bu	4-MeC_6_H_4_	NCy_2_^b^	57
**8n**	4-Cl	Cy^b^	4-CH_3_OC_6_H_4_	N(iPr)_2_	54
**8o**	4-Cl	*n*-Bu	*n*-Bu	N(Ph)Me	65
**8p**	5-Me	*t*-Bu	Cy^b^	N(CH_2_Ph)Me	74
**8q**	4-Cl	Cy^b^	PhCH_2_	N(CH_2_Ph)_2_	67
**8r**	5-Me	Cy^b^	*t*-Bu	NEt_2_	42
**8s**	H	*n*-Bu	Ph	NPh_2_	0

^a^Isolated yields based on the azides **4**. ^b^Cyclohexyl.

**Scheme 4:**
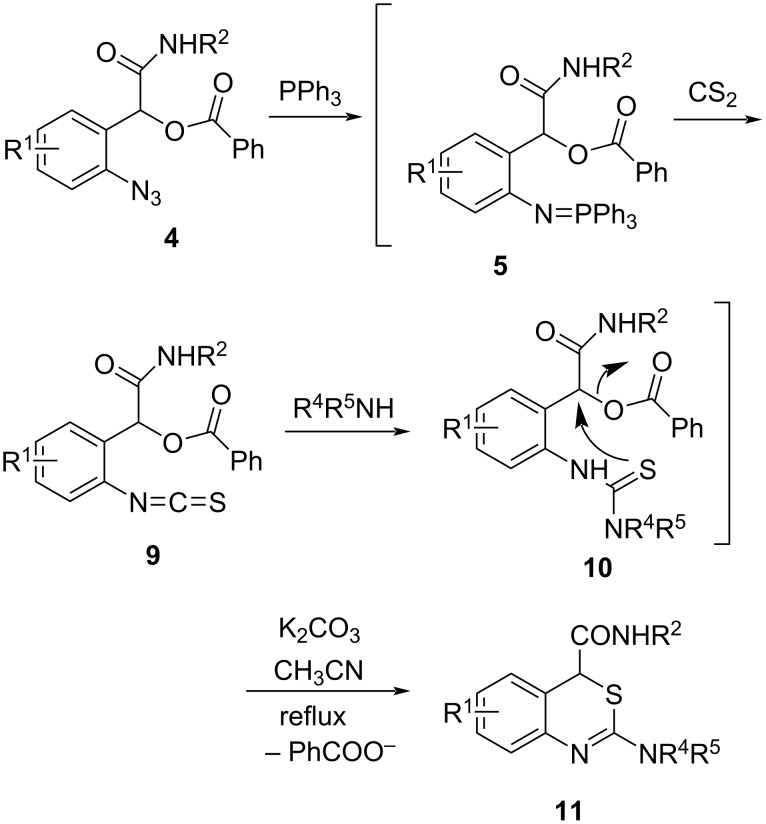
Preparation of 4*H*-3,1-benzothiazines **11**.

The aza-Wittig reaction of iminophosphoranes **5** with an excess of CS_2_ took place smoothly at 40 °C to produce isothiocyanates **9**, which were allowed to react with secondary amines to generate thiourea intermediates **10**. In the presence of K_2_CO_3_ in CH_3_CN at refluxing temperature, thioureas **10** were also successfully transformed into 4*H*-3,1-benzothiazines **11** via intramolecular nucleophilic substitution (Scheme 4). The results were listed in Table 3. Various secondary amines can be used in this one-pot cyclization to prepare 4*H*-3,1-benzothiazines **11**. As indicated in Table 3, when dialkylamines including cyclic dialkylamines (Table 3, compounds **11a**–**k**, NR^4^R^5^ = NEt_2_, NPr_2_, N(CH_2_Ph)Me, N(CH_2_Ph)_2_, piperidin-1-yl, morpholin-4-yl and pyrrolidin-1-yl) were used, good yields (72–84%) of the products were obtained, whereas mederate yield (48–54%) was obtained when the more steric dialkylamines were utilized (Table 3, compounds **11l** and **11m**, NR^4^R^5^ = N(Cy)_2_, N(iPr)_2_). In cases when phenylmethylamine (compounds **11n** and **11o**, NR^4^R^5^ = N(Ph)Me) was used, 51–56% yields of the products were obtained, but when diphenylamine was used, no product was obtained (compound **11p**, NR^4^R^5^ = NPh_2_).

**Table 3:** Yields of 4*H*-3,1-benzothiazines **11**.

	R^1^	R^2^	NR^4^R^5^	Yield^a^ (%)

**11a**	H	*t*-Bu	NEt_2_	82
**11b**	H	*t*-Bu	piperidin-1-yl	83
**11c**	H	*t*-Bu	morpholin-4-yl	84
**11d**	H	*n*-Bu	morpholin-4-yl	78
**11e**	H	Cy^b^	pyrrolidin-1-yl	77
**11f**	H	Cy^b^	N(CH_2_Ph)Me	79
**11g**	5-Me	Cy^b^	NEt_2_	72
**11h**	5-Me	*n*-Bu	piperidin-1-yl	81
**11i**	5-Me	Cy^b^	N(CH_2_Ph)_2_	78
**11j**	5-Me	*t*-Bu	NPr_2_	75
**11k**	4-Cl	Cy^b^	NEt_2_	83
**11l**	4-Cl	*t*-Bu	NCy_2_^b^	54
**11m**	5-Me	Cy^b^	N(iPr)_2_	48
**11n**	H	Cy^b^	N(Ph)Me	56
**11o**	5-Me	Cy^b^	N(Ph)Me	51
**11p**	H	*n*-Bu	NPh_2_	0

^a^Isolated yields based on the azides **4**. ^b^Cyclohexyl.

## Conclusion

In conclusion, we have developed a new Passerini/Staudinger/aza-Wittig/addition/nucleophilic substitution sequence for the synthesis of polysubstituted 3,4-dihydroquinazolines and 4*H*-3,1-benzothiazines. By this method, 3,4-dihydroquinazolines and 4*H*-3,1-benzothiazines were prepared in good overall yields with the advantages of mild one-pot operation conditions and easily accessible starting materials containing various common substituents.

## Supporting Information

File 1 Experimental section and copies of NMR spectra.
