# Supplementary material for: New efficient synthesis of polysubstituted 3,4-dihydroquinazolines and 4H-3,1-benzothiazines through a Passerini/Staudinger/aza-Wittig/addition/nucleophilic substitution sequence
Source: Beilstein J Org Chem. 2022 Mar 4;18:286–92. doi: 10.3762/bjoc.18.32 (PMC8919415; doi:10.3762/bjoc.18.32)
Supplement: File 1 — Experimental section and copies of NMR spectra. [file Beilstein_J_Org_Chem-18-286-s001.pdf]

BEILSTEIN JOURNAL OF ORGANIC CHEMISTRY

Supporting Information

for

New efficient synthesis of polysubstituted 3,4-dihydroquinazolines and 4*H*-3,1-benzothiazines through a Passerini/Staudinger/aza-Wittig/addition/nucleophilic substitution sequence

Long Zhao, Mao-Lin Yang, Min Liu and Ming-Wu Ding

Beilstein J. Org. Chem. **2022,** *18,* 286–292. doi:10.3762/bjoc.18.32

Experimental section and copies of NMR spectra

License and Terms: This is a supporting information file under the terms of the Creative Commons Attribution License (https://creativecommons.org/licenses/by/4.0). Please note that the reuse, redistribution and reproduction in particular requires that the author(s) and source are credited and that individual graphics may be subject to special legal provisions.
The license is subject to the *Beilstein Journal of Organic Chemistry* terms and conditions: (https://www.beilstein-journals.org/bjoc/terms)

Content

1. Experimental section……………………………………………………………………….……….S2

1.1 General information……………………………………………………………………………..S2

1.2 Genaral procedure for preparation of azides **4** via Passerini reaction…………………………..S2

1.3 Genaral procedure for preparation of 3,4-dihydroquinazolines **8**……………………………….S3

1.4 Genaral procedure for preparation of 4*H*-3,1-benzothiazines **11**………………………………..S7

1.5 Reference……………………………………………………………………………………...…S10

2. Copies of ^{1}H and ^{13}C NMR spectrum of compound **4**, **8** and **11**………………………….…………S11

1 Experimental section

1.1 General information

Melting points were determined using an X-4 model apparatus and were uncorrected. ^{1}H NMR were recorded in $CDCl_3$ on a Varian Mercury 600 spectrometer and resonances were relative to TMS. ^{13}C{^{1}H} NMR spectra were recorded in $CDCl_3$ on a Varian Mercury 600 (150 MHz) with complete proton decoupling spectrophotometers ($CDCl_3$: 77.0 ppm). HRMS was measured on an Agilent 6224 TOF LC/MS spectrometer. Amines, CS_2 and isocyanides were purchased from commercial suppliers and used without further purification. CH_2Cl_2, toluene and CH_3CN were used after drying and distillation. Column chromatography purifications were performed using 400-630 mesh silica gel. Analytical thin-layer chromatography (TLC) was carried out on silica gel 60 F_{254} plates, which were visualized by exposure to ultraviolet light.

1.2 Genaral procedure for preparation of azides 4 via Passerini reaction

To a solution of 2-azidobenzaldehydes **1** (1 mmol) in CH_2Cl_2 (5 mL) was added sequentially benzoic acid (**2**) (0.122 g, 1 mmol) and isocyanides **3** (1 mmol) at room temperature. After the reaction mixture was stirred for 48 hours at ambient temperature, the solvent was removed under reduced pressure and the residue was recrystallized from ether/petroleum ether to give azide **4**.

1-(2-Azidophenyl)-2-(*tert*-butylamino)-2-oxoethyl benzoate (4a)

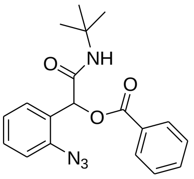

White solid (0.306 g, yield 87%), m.p. 134-136 °C, lit[1] m.p. 136-137 °C. ^{1}H NMR ($CDCl_3$, 600 MHz): δ (ppm) 8.10 (d, *J* = 7.6 Hz, 2H, Ar-H), 7.60-7.18 (m, 7H, Ar-H), 6.41 (s, 1H, CH), 6.11 (s, 1H, NH), 1.37 (s, 9H, $3CH_3$).

1-(2-Azidophenyl)-2-(cyclohexylamino)-2-oxoethyl benzoate (4b)

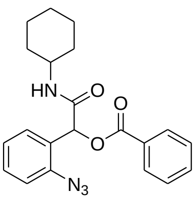

White solid (0.283 g, yield 75%), m.p. 151-153 °C; ^{1}H NMR ($CDCl_3$, 600 MHz): δ (ppm) 8.10 (d, *J* = 7.2 Hz, 2H, Ar-H), 7.61-7.18 (m, 7H, Ar-H), 6.46 (s, 1H, CH), 6.13 (d, *J* = 7.2 Hz, 1H, NH), 3.84-3.79 (m, 1H, NCH), 2.00-1.13 (m, 10H, $5CH_2$); ^{13}C NMR ($CDCl_3$, 150 MHz): δ (ppm) 166.8, 165.0, 137.9, 133.5, 130.1, 129.7, 129.4, 129.1, 128.5, 126.9, 125.1, 118.3, 71.2, 48.2, 32.8, 32.6, 25.3, 24.6. HRMS (ESI-TOF) *m/z* $[M+H]^+$ Calcd for $C_{21}H_{23}N_4O_3^+$ 379.1765; Found 379.1771.

1-(2-Azido-4-chlorophenyl)-2-(*tert*-butylamino)-2-oxoethyl benzoate (4c)

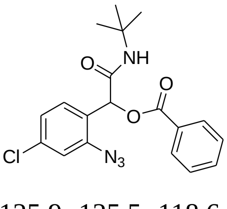

White solid (yield 0.309 g, 80%), mp 168-169 °C; ^{1}H NMR ($CDCl_3$, 600 MHz) δ (ppm) 8.07 (d, *J* = 7.2 Hz, 2H, Ar-H), 7.61 (t, *J* = 7.2 Hz, 1H, Ar-H), 7.52 (d, *J* = 8.4 Hz, 1H, Ar-H), 7.48 (t, *J* = 7.8 Hz, 2H, Ar-H), 7.19 (s, 1H, Ar-H), 7.16 (d, *J* = 7.8 Hz, 1H, Ar-H), 6.35 (s, 1H, CH), 6.11 (s, 1H, NH), 1.37 (s, 9H, $3CH_3$); ^{13}C NMR ($CDCl_3$, 150 MHz) δ (ppm) 166.5, 164.9, 139.4, 135.8, 133.7, 130.6, 129.8, 129.1, 128.6, 125.9, 125.5, 118.6, 70.9, 51.7, 28.6. HRMS (ESI-TOF) *m/z* $[M+H]^+$ Calcd for $C_{19}H_{20}ClN_4O_3^+$ 387.1218; Found 387.1217.

1-(2-Azido-4-chlorophenyl)-2-(butylamino)-2-oxoethyl benzoate (4d)

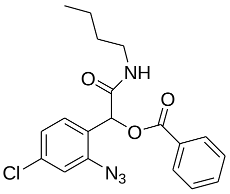

White solid (yield 0.289 g, 75%), mp 127-128 °C; ^{1}H NMR ($CDCl_3$, 600 MHz) δ (ppm) 8.08 (d, *J* = 7.8 Hz, 2H, Ar-H), 7.61 (t, *J* = 7.2 Hz, 1H, Ar-H), 7.53 (d, *J* = 7.8 Hz, 1H, Ar-H), 7.48 (t, *J* = 7.8 Hz, 2H, Ar-H), 7.18 (s, 1H, Ar-H), 7.16 (t, *J* = 9.0 Hz, 1H, Ar-H), 6.43 (s, 1H, CH), 6.27 (s, 1H, NH), 3.37-3.27 (m, 2H, NCH_2), 1.54-1.49

(m, 2H, CH_2), 1.37-1.31 (m, 2H, CH_2), 0.92 (t, J = 7.2 Hz, 3H, CH_3); ^{13}C NMR ($CDCl_3$, 150 MHz) δ (ppm) 167.3, 165.0, 139.3, 135.9, 133.7, 130.6, 129.8, 129.0, 128.6, 125.5, 125.4, 118.5, 70.8, 39.3, 31.5, 19.9, 13.7. HRMS (ESI-TOF) *m/z* $[M+H]^+$ Calcd for $C_{19}H_{20}ClN_4O_3^+$ 387.1218; Found 387.1219.

1-(2-Azido-5-methylphenyl)-2-(cyclohexylamino)-2-oxoethyl benzoate (4e)

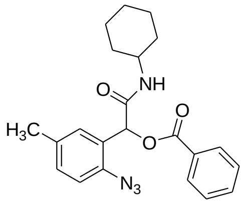

White solid (yield 0.309 g, 79%), mp 169-171 °C; ^1H NMR ($CDCl_3$, 600 MHz) δ (ppm) 8.10 (d, J = 7.8 Hz, 2H, Ar-H), 7.60 (t, J = 7.2 Hz, 1H, Ar-H), 7.47 (t, J = 7.8 Hz, 2H, Ar-H), 7.40 (s, 1H, Ar-H), 7.20 (d, J = 7.8 Hz, 1H, Ar-H), 7.09 (t, J = 7.8 Hz, 1H, Ar-H), 6.41 (s, 1H, CH), 6.12 (d, J = 8.4 Hz, 1H, NH), 3.84-3.79 (m, 1H, NCH), 2.34 (s, 3H, CH_3), 2.00-1.12 (m, 10H, $5CH_2$); ^{13}C NMR ($CDCl_3$, 150 MHz) δ (ppm) 166.9, 165.1, 135.1, 133.5, 130.9, 130.0, 129.8, 129.4, 128.5, 126.7, 118.3, 71.5, 48.2, 32.9, 32.7, 25.4, 24.6, 20.9. HRMS (ESI-TOF) *m/z* $[M+H]^+$ Calcd for $C_{22}H_{25}N_4O_3^+$ 393.1921; Found 393.1933.

1-(2-Azido-5-methylphenyl)-2-(butylamino)-2-oxoethyl benzoate (4f)

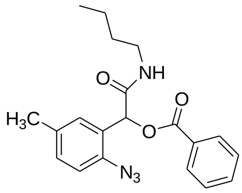

White solid (yield 0.319 g, 87%), mp 135-137 °C; ^1H NMR ($CDCl_3$, 600 MHz) δ (ppm) 8.11 (d, J = 8.4 Hz, 2H, Ar-H), 7.60 (t, J = 7.8 Hz, 1H, Ar-H), 7.47 (t, J = 7.8 Hz, 2H, Ar-H), 7.40 (s, 1H, Ar-H), 7.20 (d, J = 7.8 Hz, 1H, Ar-H), 7.09 (d, J = 7.8 Hz, 1H, Ar-H), 6.44 (s, 1H, CH), 6.22 (s, 1H, NH), 3.37-3.25 (m, 2H, NCH_2), 2.35 (s, 3H, CH_3), 1.54-1.50 (m, 2H, CH_2), 1.35-1.31 (m, 2H, CH_2), 0.91 (t, J = 7.2 Hz, 3H, CH_3); ^{13}C NMR ($CDCl_3$, 150 MHz) δ (ppm) 167.8, 165.2, 135.1, 133.5, 130.9, 130.5, 129.9, 129.3, 128.5, 126.6, 118.2, 71.3, 39.3, 31.5, 20.9, 19.9, 13.7. HRMS (ESI-TOF) *m/z* $[M+H]^+$ Calcd for $C_{20}H_{23}N_4O_3^+$ 367.1765; Found 367.1767.

1-(2-Azido-5-methylphenyl)-2-(*tert*-butylamino)-2-oxoethyl benzoate (4g)

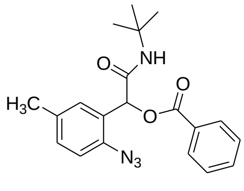

White solid (yield 0.296 g, 81%), mp 145-147 °C; ^1H NMR ($CDCl_3$, 600 MHz) δ (ppm) 8.10 (d, J = 7.8 Hz, 2H, Ar-H), 7.59 (t, J = 7.2 Hz, 1H, Ar-H), 7.47 (t, J = 7.8 Hz, 2H, Ar-H), 7.39 (s, 1H, Ar-H), 7.20 (d, J = 7.8 Hz, 1H, Ar-H), 7.09 (d, J = 7.8 Hz, 1H, Ar-H), 6.36 (s, 1H, CH), 6.09 (s, 1H, NH), 2.34 (s, 3H, CH_3), 1.37 (s, 9H, $3CH_3$); ^{13}C NMR ($CDCl_3$, 150 MHz) δ (ppm) 167.0, 165.1, 135.1, 133.5, 130.8, 129.9, 129.8, 129.4, 128.5, 126.9, 118.3, 71.5, 51.6, 28.7, 20.9. HRMS (ESI-TOF) *m/z* $[M+H]^+$ Calcd for $C_{20}H_{23}N_4O_3^+$ 367.1765; Found 367.1770.

1-(2-Azido-4-chlorophenyl)-2-(cyclohexylamino)-2-oxoethyl benzoate (4h)

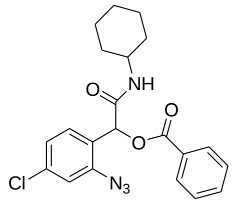

White solid (yield 0.347 g, 84%), mp 168-170 °C; ^1H NMR ($CDCl_3$, 600 MHz) δ (ppm) 8.07 (d, J = 7.2 Hz, 2H, Ar-H), 7.61 (t, J = 7.2 Hz, 1H, Ar-H), 7.53-7.46 (m, 3H, Ar-H), 7.18 (s, 1H, Ar-H), 7.16 (d, J = 8.4 Hz, 1H, Ar-H), 6.40 (s, 1H, CH), 6.12 (d, J = 7.8 Hz, 1H, NH), 3.83-3.79 (m, 1H, NCH), 1.99-1.12 (m, 10H, $5CH_2$); ^{13}C NMR ($CDCl_3$, 150 MHz) δ (ppm) 166.4, 165.0, 139.4, 135.9, 133.7, 130.7, 129.8, 129.1, 128.6, 125.7, 125.5, 118.6, 70.8, 48.3, 32.9, 32.7, 25.4, 24.6. HRMS (ESI-TOF) *m/z* $[M+H]^+$ Calcd for $C_{21}H_{22}ClN_4O_3^+$ 413.1375; Found 413.1382.

1.3 Genaral procedure for preparation of 3,4-dihydroquinazolines 8

To a stirred solution of azide **4** (1 mmol) in toluene (5 mL) was added dropwise triphenylphosphine (0.26 g, 1 mmol) in toluene (5 mL) at room temperature. After the reaction mixture was stirred for 2–4 h at ambient temperature, isocyanate (1 mmol) was added and the reaction mixture was stirred for 2–8 h at room temperature (for aromatic isocyanate) or 60–80 °C (for aliphatic isocyanate). Then secondary amine (1 mmol) was added and the reaction mixture was stirred for 1–6 h at room temperature. After the addition reaction was completed, the sovent was removed and CH_3CN (5 mL) with K_2CO_3 (0.136 g, 1 mmol) was

added and the mixture was left to stirred at 80 °C for 1–4 h. The solvent was removed under reduced pressure and the residual was purified by column chromatography with EtOAc/ petroleum ether to give 3,4-dihydroquinazolines **8**.

***N-tert*-Butyl-2-(diethylamino)-3,4-dihydro-3-phenylquinazoline-4-carboxamide (8a)**

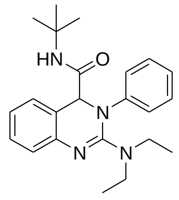

White solid (0.317 g, yield 84%), m.p. 118-121 °C; ^{1}H NMR ($CDCl_3$, 600 MHz): δ (ppm) 7.31-6.96 (m, 9H, Ar-H), 5.46 (s, 1H, NH), 4.99 (s, 1H, CH), 3.57-3.55 (m, 2H, NCH_2), 3.14-3.08 (m, 2H, NCH_2), 1.23 (s, 9H, $3CH_3$), 1.01 (t, J = 6.6 Hz, 6H, $2CH_3$); ^{13}C NMR ($CDCl_3$, 150 MHz): δ (ppm) 169.5, 153.2, 146.5, 144.4, 129.1, 128.9, 126.2, 124.2, 123.3, 123.2, 122.1, 67.3, 51.3, 41.6, 41.2, 28.5, 28.1, 12.5, 12.4. Anal. Calcd for $C_{23}H_{30}N_4O$: C, 72.98; H, 7.99; N, 14.80. Found: C, 72.75; H, 7.72; N, 14.67. HRMS (ESI-TOF) *m/z* $[M+H]^+$ Calcd for $C_{23}H_{31}N_4O^+$ 379.2492; Found 379.2494.

***N-tert*-Butyl-3-(4-chlorophenyl)-2-(diethylamino)-3,4-dihydroquinazoline-4-carboxamide (8b)**

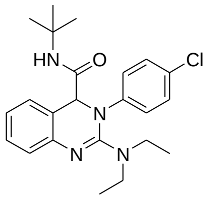

White solid (0.331 g, yield 80%), m.p. 169-171 °C; ^{1}H NMR ($CDCl_3$, 600 MHz): δ (ppm) 7.32-6.99 (m, 8H, Ar-H), 5.37 (s, 1H, NH), 4.93 (s, 1H, CH), 3.61-3.53 (m, 2H, NCH_2), 3.09-3.05 (m, 2H, NCH_2), 1.21 (s, 9H, $3CH_3$), 1.01 (t, J = 7.8 Hz, 6H, $2CH_3$); ^{13}C NMR ($CDCl_3$, 150 MHz): δ (ppm) 169.3, 152.9, 145.1, 144.3, 129.6, 129.4, 129.0, 126.1, 124.6, 123.4, 122.4, 122.0, 67.2, 51.4, 41.9, 28.3, 12.6. HRMS (ESI-TOF) *m/z* $[M+H]^+$ Calcd for $C_{23}H_{30}ClN_4O^+$ 413.2103; Found 413.2100.

***N-tert*-Butyl-2-(diethylamino)-3,4-dihydro-3-*m*-tolylquinazoline-4-carboxamide (8c)**

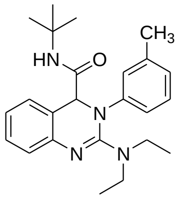

White solid (0.297 g, yield 76%), m.p. 106-108 °C; ^{1}H NMR ($CDCl_3$, 600 MHz): δ (ppm) 7.30-6.95 (m, 7H, Ar-H), 6.89 (d, J = 7.2 Hz, 1H, Ar-H), 5.46 (s, 1H, NH), 4.99 (s, 1H, CH), 3.62-3.56 (m, 2H, NCH_2), 3.13-3.07 (m, 2H, NCH_2), 2.30 (s, 3H, CH_3), 1.23 (s, 9H, $3CH_3$), 1.01 (t, J = 6.6 Hz, 6H, $2CH_3$); ^{13}C NMR ($CDCl_3$, 150 MHz): δ (ppm) 169.5, 153.2, 146.5, 144.5, 138.9, 129.1, 128.7, 126.2, 125.0, 123.8, 123.1, 122.2, 122.1, 120.3, 67.3, 51.3, 41.7, 28.4, 21.3, 12.5. HRMS (ESI-TOF) *m/z* $[M+H]^+$ Calcd for $C_{24}H_{33}N_4O^+$ 393.2649; Found 393.2650.

***N-tert*-Butyl-2-(diethylamino)-3,4-dihydro-3-*p*-tolylquinazoline-4-carboxamide (8d)**

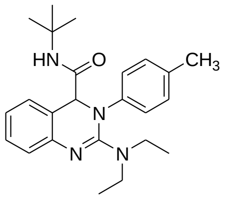

White solid (0.309 g, yield 79%), m.p. 121-123 °C; ^{1}H NMR ($CDCl_3$, 600 MHz): δ (ppm) 7.30-6.95 (m, 8H, Ar-H), 5.47 (s, 1H, NH), 4.95 (s, 1H, CH), 3.60-3.55 (m, 2H, NCH_2), 3.12-3.06 (m, 2H, NCH_2), 2.29 (s, 3H, CH_3), 1.23 (s, 9H, $3CH_3$), 1.01 (t, J = 6.6 Hz, 6H, $2CH_3$); ^{13}C NMR ($CDCl_3$, 150 MHz): δ (ppm) 169.5, 153.3, 144.4, 144.0, 133.9, 129.4, 129.1, 126.1, 123.2, 123.1, 123.0, 122.0, 67.4, 51.2, 41.8, 28.7, 28.4, 28.1, 27.8, 21.1, 20.5, 12.7, 12.3. HRMS (ESI-TOF) *m/z* $[M+H]^+$ Calcd for $C_{24}H_{33}N_4O^+$ 393.2649; Found 393.2649.

***N-tert*-Butyl-3,4-dihydro-3-phenyl-2-(piperidin-1-yl)quinazoline-4-carboxamide (8e)**

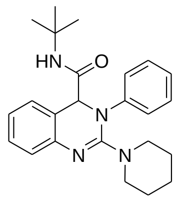

White solid (0.281 g, yield 72%), m.p. 177-179 °C; ^{1}H NMR ($CDCl_3$, 600 MHz): δ (ppm) 7.28-6.97 (m, 9H, Ar-H), 5.45 (s, 1H, NH), 5.02 (s, 1H, CH), 3.38 (t, J = 7.2 Hz, 4H, $2NCH_2$), 1.50-1.35 (m, 6H, $3CH_2$), 1.24 (s, 9H, $3CH_3$); ^{13}C NMR ($CDCl_3$, 150 MHz): δ (ppm) 169.5, 153.3, 146.1, 144.2, 129.1, 128.9, 126.1, 124.0, 123.1, 122.8, 122.5, 122.2, 66.8, 51.3, 47.4, 28.3, 25.2, 24.6. HRMS (ESI-TOF) *m/z* $[M+H]^+$ Calcd for $C_{24}H_{31}N_4O^+$ 391.2492; Found 391.2491.

***N-tert*-Butyl-2-(dipropylamino)-3,4-dihydro-3-*p*-tolylquinazoline-4-carboxamide (8f)**

White solid (0.358 g, yield 85%), m.p. 108-109 °C; ^{1}H NMR ($CDCl_3$, 600 MHz): δ (ppm) 7.30-6.94 (m, 8H, Ar-H), 5.40 (s, 1H, NH), 4.94 (s, 1H, CH), 3.56-3.49 (m, 2H, NCH_2), 2.87-2.83 (m, 2H, NCH_2), 2.29 (s, 3H,

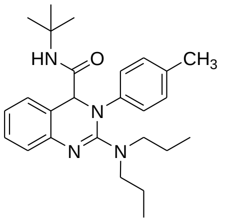

CH_3), 1.54-1.41 (m, 4H, $2CH_2$), 1.21 (s, 9H, $3CH_3$), 0.82 (t, J = 7.2 Hz, 6H, $2CH_3$); ^{13}C NMR ($CDCl_3$, 150 MHz): δ (ppm) 169.5, 154.0, 144.6, 144.3, 134.0, 129.5, 129.1, 126.2, 123.5, 123.1, 122.0, 121.9, 67.6, 51.2, 49.4, 28.3, 20.8, 20.6, 11.5. HRMS (ESI-TOF) *m/z* $[M+H]^+$ Calcd for $C_{26}H_{37}N_4O^+$ 421.2961; Found 421.2962.

***N*-*tert*-Butyl-2-(dibutylamino)-3,4-dihydro-3-*p*-tolylquinazoline-4-carboxamide (8g)**

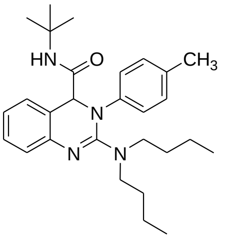

White solid (0.309 g, yield 69%), m.p. 114-116 °C; ^1H NMR ($CDCl_3$, 600 MHz): δ (ppm) 7.30-6.95 (m, 8H, Ar-H), 5.40 (s, 1H, NH), 4.94 (s, 1H, CH), 3.62-3.58 (m, 2H, NCH_2), 2.90-2.86 (m, 2H, NCH_2), 2.29 (s, 3H, CH_3), 1.50-1.21 (m, 17H, $4CH_2$ and $3CH_3$), 0.87 (t, J = 7.2 Hz, 6H, $2CH_3$); ^{13}C NMR ($CDCl_3$, 150 MHz): δ (ppm) 169.5, 153.9, 144.6, 144.3, 134.0, 129.4, 129.1, 126.2, 123.6, 123.1, 122.0, 121.9, 67.6, 51.2, 47.4, 29.6, 28.3, 20.8, 20.3, 13.9. HRMS (ESI-TOF) *m/z* $[M+H]^+$ Calcd for $C_{28}H_{41}N_4O^+$ 449.3275; Found 449.3275.

***N*-Cyclohexyl-2-(diethylamino)-3,4-dihydro-3-*p*-tolylquinazoline-4-carboxamide (8h)**

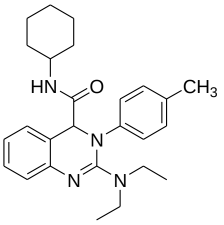

White solid (0.296 g, yield 71%), m.p. 125-127 °C; ^1H NMR ($CDCl_3$, 600 MHz): δ (ppm) 7.30-6.95 (m, 8H, Ar-H), 5.51 (d, J = 7.2 Hz, 1H, NH), 5.01 (s, 1H, CH), 3.70-3.65 (m, 1H, NCH), 3.57-3.54 (m, 2H, NCH_2), 3.14-3.08 (m, 2H, NCH_2), 2.30 (s, 3H, CH_3), 1.79-1.08 (m, 10H, $5CH_2$), 0.99 (t, J = 6.6 Hz, 6H, $2CH_3$); ^{13}C NMR ($CDCl_3$, 150 MHz): δ (ppm) 169.3, 153.3, 144.5, 144.0, 133.9, 129.4, 129.0, 126.0, 123.3, 123.0, 121.9, 121.7, 66.9, 48.3, 41.8, 32.3, 25.2, 24.3, 20.7, 12.5. HRMS (ESI-TOF) *m/z* $[M+H]^+$ Calcd for $C_{26}H_{35}N_4O^+$ 419.2805; Found 419.2804.

***N*-Cyclohexyl-2-(diethylamino)-3,4-dihydro-3-phenylquinazoline-4-carboxamide (8i)**

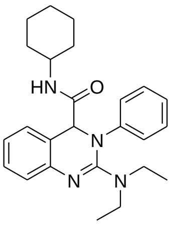

White solid (0.347 g, yield 86%), m.p. 145-147 °C; ^1H NMR ($CDCl_3$, 600 MHz): δ (ppm) 7.29-6.96 (m, 9H, Ar-H), 5.50 (d, J = 7.8 Hz, 1H, NH), 5.06 (s, 1H, CH), 3.71-3.66 (m, 1H, NCH), 3.57-3.52 (m, 2H, NCH_2), 3.16-3.10 (m, 2H, NCH_2), 1.80-1.10 (m, 10H, $5CH_2$), 1.00 (t, J = 6.6 Hz, 6H, $2CH_3$); ^{13}C NMR ($CDCl_3$, 150 MHz): δ (ppm) 169.3, 153.2, 146.5, 144.5, 129.1, 128.9, 126.0, 124.3, 123.3, 123.2, 122.1, 121.9, 66.8, 48.4, 41.9, 32.4, 32.3, 25.3, 24.4, 12.5. HRMS (ESI-TOF) *m/z* $[M+H]^+$ Calcd for $C_{25}H_{33}N_4O^+$ 405.2649; Found 405.2647.

3-(4-Chlorophenyl)-*N*-cyclohexyl-2-(diethylamino)-3,4-dihydroquinazoline-4-carboxamide (8j)

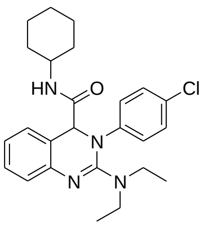

White solid (0.343 g, yield 78%), m.p. 136-138 °C; ^1H NMR ($CDCl_3$, 600 MHz): δ (ppm) 7.32-6.98 (m, 8H, Ar-H), 5.43 (d, J = 7.2 Hz, 1H, NH), 5.00 (s, 1H, CH), 3.69-3.64 (m, 1H, NCH), 3.55-3.51 (m, 2H, NCH_2), 3.13-3.07 (m, 2H, NCH_2), 1.80-1.07 (m, 10H, $5CH_2$), 1.01 (t, J = 7.2 Hz, 6H, $2CH_3$); ^{13}C NMR ($CDCl_3$, 150 MHz): δ (ppm) 169.1, 152.9, 145.0, 144.4, 129.6, 129.3, 128.9, 126.0, 124.6, 123.3, 122.4, 121.7, 66.6, 48.5, 42.0, 32.8, 32.3, 24.4, 13.0, 12.2. HRMS (ESI-TOF) *m/z* $[M+H]^+$ Calcd for $C_{25}H_{32}ClN_4O^+$ 439.2259; Found 439.2254.

***N*-Cyclohexyl-2-(diethylamino)-3,4-dihydro-3-(4-(trifluoromethoxy)phenyl)quinazoline-4-carboxamide (8k)**

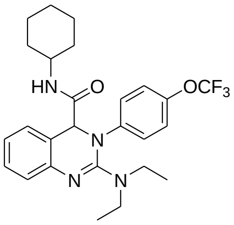

White solid (0.391 g, yield 80%), m.p. 140-142 °C; ^1H NMR ($CDCl_3$, 600 MHz): δ (ppm) 7.35-7.00 (m, 8H, Ar-H), 5.43 (d, J = 7.2 Hz, 1H, NH), 5.02 (s, 1H, CH), 3.70-3.65 (m, 1H, NCH), 3.56-3.49 (m, 2H, NCH_2), 3.15-3.09 (m, 2H, NCH_2), 1.78-1.00 (m, 16H, $5CH_2$ and $2CH_3$); ^{13}C NMR ($CDCl_3$, 150 MHz): δ (ppm) 169.0, 152.9, 145.4, 145.0, 144.4, 129.3, 125.9, 124.4, 123.4, 122.4, 122.0 (q, $^1J_{F\text{-}C}$ = 255.1

Hz), 121.6, 121.5, 66.6, 48.4, 42.0, 32.7, 32.3, 25.3, 24.4, 12.8. HRMS (ESI-TOF) *m/z* $[M+H]^+$ Calcd for $C_{26}H_{32}F_3N_4O_2^+$ 489.2472; Found 489.2470.

***N*-*tert*-butyl-3,4-dihydro-2-morpholino-3-*p*-tolylquinazoline-4-carboxamide (8l)**

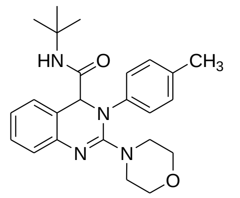

White solid (0.284 g, yield 70%), m.p. 138-139 °C; ^1H NMR ($CDCl_3$, 600 MHz): δ (ppm) 7.32-7.00 (m, 8H, Ar-H), 5.45 (s, 1H, NH), 4.99 (s, 1H, CH), 3.55-3.36 (m, 8H, $2CH_2O$ and $2CH_2N$), 2.31 (s, 3H, CH_3), 1.24 (s, 9H, $3CH_3$); ^{13}C NMR ($CDCl_3$, 150 MHz): δ (ppm) 169.5, 153.2, 143.8, 143.1, 134.1, 129.6, 129.4, 129.2, 126.0, 123.3, 122.7, 122.6, 66.7, 66.3, 51.3, 46.7, 28.3, 20.7. HRMS (ESI-TOF) *m/z* $[M+H]^+$ Calcd for $C_{24}H_{31}N_4O_2^+$ 407.2442; Found 407.2440.

***N*-*tert*-Butyl-2-(dicyclohexylamino)-3,4-dihydro-3-*p*-tolylquinazoline-4-carboxamide (8m)**

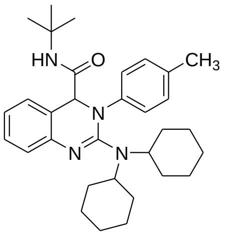

White solid (0.232 g, yield 57%), m.p. 112-115 °C; ^1H NMR ($CDCl_3$, 600 MHz): δ (ppm) 7.59-6.91 (m, 8H, Ar-H), 5.88 (s, 0.25H, 0.25NH), 5.67 (s, 0.75H, 0.75NH), 4.82 and 4.79 (ss, 1H, CH), 2.38-2.27 (m, 5H, 2CHN and CH_3), 1.77-0.94 (m, 29H, $10CH_2$ and $3CH_3$); ^{13}C NMR ($CDCl_3$, 150 MHz): δ (ppm) 173.7 (minor), 169.7, 152.9, 145.4 (minor), 144.7, 143.4 (minor), 136.3 (minor), 134.6, 132.2 (minor), 130.5, (minor), 129.4, 128.8, 127.8 (minor), 126.6, 124.8, 123.4, 123.2 (minor), 122.9, 122.2, 121.7 (minor), 118.9 (minor), 71.0 (minor), 68.3, 59.7 (minor), 57.8, 51.2, 51.3, 33.8, 28.7, 28.5, 26.8, 25.7, 20.9. HRMS (ESI-TOF) *m/z* $[M+H]^+$ Calcd for $C_{32}H_{45}N_4O^+$ 501.3588; Found 501.3586.

7-Chloro-*N*-cyclohexyl-2-(diisopropylamino)-3-(4-methoxyphenyl)-3,4-dihydroquinazoline-4-carbox-amide (8n)

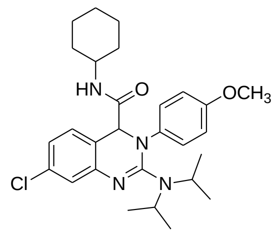

White solid (yield 0.269 g, 54%), mp 82-84 °C; ^1H NMR ($CDCl_3$, 600 MHz) δ (ppm) 7.17 (s, 1H, Ar-H), 7.05 (d, *J* = 7.8 Hz, 2H, Ar-H), 6.92-6.88 (m, 2H, Ar-H), 6.78 (d, *J* = 8.4 Hz, 2H, Ar-H), 5.68 (d, *J* = 7.2 Hz, 1H, NH), 4.82-3.69 (m, 6H, OCH_3 and 3NCH), 1.82-1.02 (m, 22H, $5CH_2$ and $4CH_3$); ^{13}C NMR ($CDCl_3$, 150 MHz) δ (ppm) 169.1, 157.2, 153.0, 146.2, 139.9, 134.2, 127.5, 125.9, 122.9, 121.5, 120.2, 114.3, 67.6, 55.4, 48.5, 32.7, 32.6, 25.4, 24.6, 23.2, 19.0. HRMS (ESI-TOF) *m/z* $[M+H]^+$ Calcd for $C_{28}H_{38}ClN_4O_2^+$ 497.2678; Found 497.2669.

***N*,3-Dibutyl-7-chloro-2-(methyl(phenyl)amino)-3,4-dihydroquinazoline-4-carboxamide (8o)**

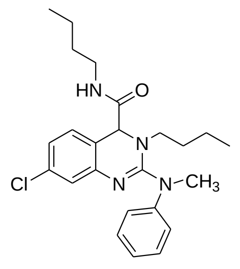

White solid (yield 0.277 g, 65%), mp 127-129 °C; ^1H NMR ($CDCl_3$, 600 MHz) δ (ppm) 7.23 (t, *J* = 7.2 Hz, 2H, Ar-H), 7.17 (d, *J* = 8.4 Hz, 1H, Ar-H), 7.13 (s, 1H, Ar-H), 7.05 (d, *J* = 7.8 Hz, 1H, Ar-H), 6.96 (d, *J* = 7.2 Hz, 2H, Ar-H), 6.84 (t, *J* = 6.6 Hz, 1H, Ar-H), 5.08 (s, 1H, CH), 3.98 (s, 1H, NH), 3.67-3.23 (m, 4H, $2NCH_2$), 2.54 (s, 3H, NCH_3), 1.57-1.06 (m, 8H, $4CH_2$), 0.98 (t, *J* = 7.2 Hz, 3H, CH_3), 0.76 (t, *J* = 7.2 Hz, 3H, CH_3); ^{13}C NMR ($CDCl_3$, 150 MHz) δ (ppm) 168.3, 149.6, 146.6, 146.1, 135.3, 130.8, 128.9, 124.9, 123.7, 123.5, 119.5, 116.9, 71.0, 46.6, 41.7, 38.4, 31.3, 29.8, 20.2, 19.7, 13.8, 13.6. HRMS (ESI-TOF) *m/z* $[M+H]^+$ Calcd for $C_{24}H_{32}ClN_4O^+$ 427.2259; Found 427.2261.

2-(Benzyl(methyl)amino)-*N*-(*tert*-butyl)-3-cyclohexyl-6-methyl-3,4-dihydroquinazoline-4-carbox-amide (8p)

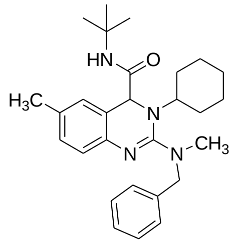

White solid (yield 0.329 g, 74%), mp 51-53 °C; ^1H NMR ($CDCl_3$, 600 MHz) δ (ppm) 7.47-7.11 (m, 8H, Ar-H and NH), 7.03 (d, *J* = 7.8 Hz, 1H, Ar-H), 4.62 (s, 1H, CH), 3.59-3.47 (m, 3H, NCH_2 and NCH), 2.30 (s, 3H, NCH_3), 2.07 (s, 3H, CH_3), 2.00-1.24 (m, 19H, $5CH_2$ and $3CH_3$); ^{13}C NMR ($CDCl_3$, 150 MHz) δ (ppm) 170.9, 139.3, 137.5, 136.0, 133.9, 130.6, 129.4, 129.3, 128.5, 128.3, 127.0, 123.9, 69.0,

59.7, 56.7, 50.6, 39.3, 35.1, 28.8, 25.3, 24.4, 21.1. HRMS (ESI-TOF) *m/z* $[M+H]^+$ Calcd for $C_{28}H_{39}N_4O^+$ 447.3118; Found 447.3115.

3-Benzyl-7-chloro-*N*-cyclohexyl-2-(dibenzylamino)-3,4-dihydroquinazoline-4-carboxamide (8q)

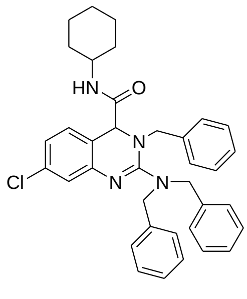

White solid (yield 0.385 g, 67%), mp 60-62 °C; ^1H NMR ($CDCl_3$, 600 MHz) δ (ppm) 7.33-7.18 (m, 15H, Ar-H), 7.07 (d, *J* = 6.6 Hz, 2H, Ar-H), 6.76 (d, *J* = 7.8 Hz, 1H, NH), 6.52 (s, 1H, Ar-H), 4.78 (s, 1H, CH), 4.46 (d, *J* = 15.6 Hz, 1H, NCH_2^a), 4.28 (d, *J* = 15.6 Hz, 1H, NCH_2^b), 4.23-4.14 (m, 2H, NCH_2), 4.14 (t, *J* = 6.6 Hz, 1H, NCH), 3.82 (d, *J* = 13.8 Hz, 1H, NCH_2^a), 3.74-3.69 (m, 1H, NCH), 3.57 (d, *J* = 14.4 Hz, 1H, NCH_2^b), 1.86-1.02 (m, 10H, $5CH_2$); ^{13}C NMR ($CDCl_3$, 150 MHz) δ (ppm) 171.3, 155.0, 151.4, 139.1, 138.6, 137.9, 133.3, 130.7, 128.7, 128.6, 128.2, 127.7, 127.5, 127.4, 127.2, 126.8, 121.5, 120.8, 64.7, 54.8, 52.0, 48.8, 47.6, 33.2, 32.9, 25.5, 24.8. HRMS (ESI-TOF) *m/z* $[M+H]^+$ Calcd for $C_{36}H_{38}ClN_4O^+$ 577.2729; Found 577.2725.

3-(*tert*-Butyl)-*N*-cyclohexyl-2-(diethylamino)-6-methyl-3,4-dihydroquinazoline-4-carboxamide (8r)

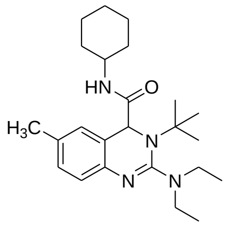

White solid (yield 0.168 g, 42%), mp 51-52 °C; ^1H NMR ($CDCl_3$, 600 MHz) δ (ppm) 7.53 (d, *J* = 7.2 Hz, 1H, NH), 7.08 (d, *J* = 8.4 Hz, 1H, Ar-H), 7.04 (s, 1H, Ar-H), 7.00 (d, *J* = 7.8 Hz, 1H, Ar-H), 4.80 (s, 1H, CH), 3.84-3.80 (m, 1H, NCH), 2.61-2.58 (m, 2H, NCH_2), 2.43-2.39 (m, 2H, NCH_2), 2.28 (s, 3H, CH_3), 1.90-1.26 (m, 19H, $5CH_2$ and $3CH_3$), 1.01 (t, *J* = 7.2 Hz, 5H, $5/6\times2CH_3$), 0.88 (t, *J* = 6.6 Hz, 1H, $1/6\times2CH_3$); ^{13}C NMR ($CDCl_3$, 150 MHz) δ (ppm) 171.6, 137.5, 136.4, 133.8, 130.7, 129.8, 129.2, 123.5, 64.8, 57.3 (minor), 47.2 (minor), 43.8, 33.2, 33.0, 31.6, 30.1 (minor), 29.7, 29.3 (minor), 25.6, 24.7, 22.7 (minor), 21.0, 14.1 (minor), 12.5. HRMS (ESI-TOF) *m/z* $[M+H]^+$ Calcd for $C_{24}H_{39}N_4O^+$ 399.3118; Found 399.3110.

1.4 Genaral procedure for preparation of 4*H*-3,1-benzothiazines 11

To a stirred solution of azide **4** (1 mmol) in CH_2Cl_2 (3 mL) was added dropwise triphenylphosphine (0.26 g, 1 mmol) in CH_2Cl_2 (2 mL) at room temperature. After the reaction mixture was stirred for 2–4 h at ambient temperature, CS_2 (0.76 g, 10 mmol) was added and the reaction mixture was stirred at 40 °C for 24 h. The sovent and excess CS_2 was completely removed and CH_3CN (5 mL) was added. Then secondary amine (1 mmol) was added and the reaction mixture was stirred for 1–6 h at room temperature. After the addition reaction was completed, K_2CO_3 (0.136 g, 1 mmol) was added and the mixture was left to stirred at 80 °C for 1–4 h. the solvent was removed under reduced pressure and the residual was purified by column chromatography with EtOAc/ petroleum ether to give 4*H*-3,1-benzothiazines **11**.

***N*-(*tert*-Butyl)-2-(diethylamino)- 4*H*-3,1-benzothiazine-4-carboxamide (11a)**

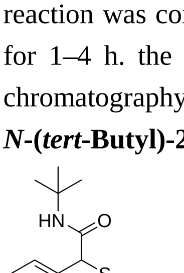

White solid (yield 0.262 g, 82%), mp 136-138 °C; ^1H NMR ($CDCl_3$, 600 MHz) δ (ppm) 7.29 (t, *J* = 7.2 Hz, 1H, Ar-H), 7.14 (d, *J* = 7.2 Hz, 2H, Ar-H), 7.01 (t, *J* = 6.6 Hz, 1H, Ar-H), 5.49 (s, 1H, NH), 4.46 (s, 1H, CH), 3.80-3.50 (m, 4H, $2\times NCH_2$), 1.23 (t, *J* = 7.2 Hz, 6H, $2CH_3$), 1.20 (s, 9H, $3CH_3$); ^{13}C NMR ($CDCl_3$, 150 MHz) δ (ppm) 168.4, 152.7, 145.7, 129.3, 127.5, 125.2, 122.9, 118.0, 51.4, 48.5, 43.7, 28.3, 14.1. HRMS (ESI-TOF) *m/z* $[M+H]^+$ Calcd for $C_{17}H_{26}N_3OS^+$ 320.1791; Found 320.1785.

***N*-(*tert*-Butyl)-2-(piperidin-1-yl)-4*H*-3,1-benzothiazine-4-carboxamide (11b)**

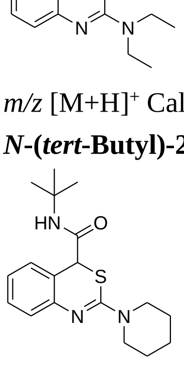

White solid (yield 0.274 g, 83%), mp 141-142 °C; ^1H NMR ($CDCl_3$, 600 MHz) δ (ppm) 7.30 (t, *J* = 7.8 Hz, 1H, Ar-H), 7.14 (d, *J* = 7.8 Hz, 2H, Ar-H), 7.04 (t, *J* = 7.8 Hz, 1H, Ar-H), 5.51 (s, 1H, NH), 4.50 (s, 1H, CH), 3.83-3.73 (m, 4H, $2\times NCH_2$), 1.71-1.59 (m, 6H, $3CH_2$), 1.22 (s, 9H, $3CH_3$); ^{13}C NMR ($CDCl_3$, 150 MHz) δ (ppm) 168.3, 153.7, 145.4,

129.3, 127.5, 125.3, 123.3, 118.1, 51.4, 48.5, 48.0, 28.3, 26.0, 24.9. HRMS (ESI-TOF) *m/z* $[M+H]^+$ Calcd for $C_{18}H_{26}N_3OS^+$ 332.1791; Found 332.1788.

***N*-(*tert*-Butyl)-2-morpholino-4*H*-3,1-benzothiazine-4-carboxamide (11c)**

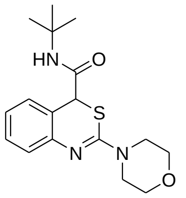

White solid (yield 0.279 g, 84%), mp 157-159 °C; ^1H NMR ($CDCl_3$, 600 MHz) δ (ppm) 7.32 (t, *J* = 7.8 Hz, 1H, Ar-H), 7.16 (d, *J* = 7.8 Hz, 2H, Ar-H), 7.09 (t, *J* = 7.8 Hz, 1H, Ar-H), 5.43 (s, 1H, NH), 4.52 (s, 1H, CH), 3.83-3.73 (m, 8H, $4CH_2$), 1.22 (s, 9H, $3CH_3$); ^{13}C NMR ($CDCl_3$, 150 MHz) δ (ppm) 167.9, 154.4, 144.8, 129.5, 127.5, 125.5, 124.0, 118.3, 66.7, 51.5, 48.4, 47.4, 28.3. HRMS (ESI-TOF) *m/z* $[M+H]^+$ Calcd for $C_{17}H_{24}N_3O_2S^+$ 334.1584; Found 334.1582.

***N*-Butyl-2-morpholino-4*H*-3,1-benzothiazine-4-carboxamide (11d)**

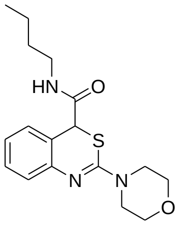

White solid (yield 0.259 g,, 78%), mp 123-125 °C; ^1H NMR ($CDCl_3$, 600 MHz) δ (ppm) 7.34 (t, *J* = 7.2 Hz, 1H, Ar-H), 7.18-7.15 (m, 2H, Ar-H), 7.10 (t, *J* = 7.2 Hz, 1H, Ar-H), 5.50 (s, 1H, NH), 4.63 (s, 1H, CH), 3.84-3.73 (m, 8H, $4CH_2$), 3.20-3.03 (m, 2H, NCH_2), 1.36-1.32 (m, 2H, CH_2), 1.24-1.19 (m, 2H, CH_2), 0.86 (t, *J* = 7.8 Hz, 3H, CH_3); ^{13}C NMR ($CDCl_3$, 150 MHz) δ (ppm) 169.0, 154.6, 144.9, 129.7, 127.7, 125.6, 124.1, 117.5, 66.6, 47.4, 47.2, 39.6, 31.3, 19.7, 13.7. HRMS (ESI-TOF) *m/z* $[M+H]^+$ Calcd for $C_{17}H_{24}N_3O_2S^+$ 334.1584; Found 334.1581.

***N*-Cyclohexyl-2-(pyrrolidin-1-yl)-4*H*-3,1-benzothiazine-4-carboxamide (11e)**

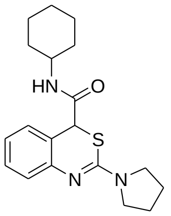

White solid (yield 0.265 g, 77%), mp 148-150 °C; ^1H NMR ($CDCl_3$, 600 MHz) δ (ppm) 7.30 (t, *J* = 7.8 Hz, 1H, Ar-H), 7.16 (d, *J* = 7.8 Hz, 1H, Ar-H), 7.12 (d, *J* = 7.2 Hz, 1H, Ar-H), 7.01 (t, *J* = 7.8 Hz, 1H, Ar-H), 5.55 (d, *J* = 6.6 Hz, 1H, NH), 4.53 (s, 1H, CH), 3.76-3.56 (m, 5H, $2\times NCH_2$ and NCH), 1.98-1.94 (m, 4H, $2CH_2$), 1.71-0.96 (m, 10H, $5CH_2$); ^{13}C NMR ($CDCl_3$, 150 MHz) δ (ppm) 168.5, 151.9, 145.7, 129.5, 127.7, 125.3, 122.9, 117.2, 48.1, 48.0, 47.6, 32.2, 25.3, 25.0, 24.1. HRMS (ESI-TOF) *m/z* $[M+H]^+$ Calcd for $C_{19}H_{26}N_3OS^+$ 344.1791; Found 344.1785.

2-(Benzyl(methyl)amino)-*N*-cyclohexyl-4*H*-3,1-benzothiazine-4-carboxamide (11f)

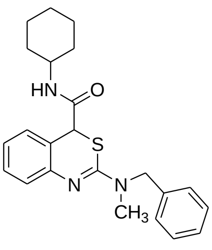

White solid (yield 0.311 g, 79%), mp 120-121 °C; ^1H NMR ($CDCl_3$, 600 MHz) δ (ppm) 7.31-7.15 (m, 8H, Ar-H), 7.06 (t, *J* = 7.2 Hz, 1H, Ar-H), 5.48 (d, *J* = 6.6 Hz, 1H, NH), 5.00 (d, *J* = 15.0 Hz, 1H, NCH_2^a), 4.73 (d, *J* = 15.6 Hz, 1H, NCH_2^b), 4.60 (s, 1H, CH), 3.69-3.64 (m, 1H, NCH), 3.16 (s, 3H, NCH_3), 1.73-0.92 (m, 10H, $5CH_2$); ^{13}C NMR ($CDCl_3$, 150 MHz) δ (ppm) 168.2, 154.4, 145.6, 137.3, 129.5, 128.6, 127.5, 127.4, 125.5, 123.4, 117.4, 54.2, 48.4, 47.8, 36.5, 32.3, 25.3, 24.3. HRMS (ESI-TOF) *m/z* $[M+H]^+$ Calcd for $C_{23}H_{28}N_3OS^+$ 394.1948; Found 394.1945.

***N*-Cyclohexyl-2-(diethylamino)-6-methyl-4*H*-3,1-benzothiazine-4-carboxamide (11g)**

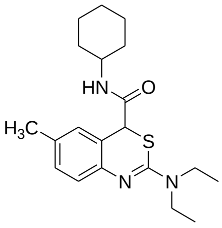

White solid (yield 0.258 g, 72%), mp 142-143 °C; ^1H NMR ($CDCl_3$, 600 MHz) δ (ppm) 7.10 (d, *J* = 7.8 Hz, 1H, Ar-H), 7.04 (d, *J* = 8.4 Hz, 1H, Ar-H), 6.94 (s, 1H, Ar-H), 5.52 (d, *J* = 6.0 Hz, 1H, NH), 4.49 (s, 1H, CH), 3.75-3.48 (m, 5H, $2\times NCH_2$ and NCH), 2.31 (s, 3H, CH_3), 1.73-1.26 (m, 7H, $7/10\times 5CH_2$), 1.20 (t, *J* = 7.2 Hz, 6H, $2CH_3$), 1.11-0.93 (m, 3H, $3/10\times 5CH_2$); ^{13}C NMR ($CDCl_3$, 150 MHz) δ (ppm) 168.6, 152.2, 143.4, 132.6, 130.2, 127.9, 125.1, 117.3, 48.2, 47.7, 43.6, 32.4, 32.3, 25.4, 24.3, 20.8, 14.1. HRMS (ESI-TOF) *m/z* $[M+H]^+$ Calcd for $C_{20}H_{29}N_3OS^+$ 360.2104; Found 360.2098.

***N*-Butyl-6-methyl-2-(piperidin-1-yl)-4*H*-3,1-benzothiazine-4-carboxamide (11h)**

White solid (yield 0.279 g, 81%), mp 135-136 °C; ^1H NMR ($CDCl_3$, 600 MHz) δ (ppm) 7.12 (d, *J* = 7.8 Hz, 1H, Ar-H), 7.05 (d, *J* = 8.4 Hz, 1H, Ar-H), 6.94 (s, 1H, Ar-H), 5.59 (s, 1H, NH), 4.55 (s, 1H, CH), 3.77-3.71 (m, 4H, $2\times NCH_2$), 3.27-2.98 (m, 2H, NCH_2), 2.32 (s, 3H, CH_3), 1.68-1.58 (m, 6H, $3CH_2$),

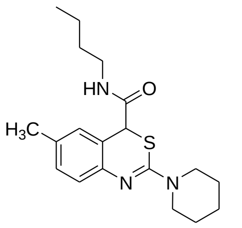

1.37-1.18 (m, 4H, $2CH_2$), 0.85 (t, *J* = 7.2 Hz, 3H, CH_3); ^{13}C NMR ($CDCl_3$, 150 MHz) δ (ppm) 169.5, 153.4, 143.1, 133.1, 130.2, 128.1, 125.3, 117.1, 47.8, 47.6, 39.5, 31.3, 25.9, 24.9, 20.8, 19.7, 13.7. HRMS (ESI-TOF) *m/z* $[M+H]^+$ Calcd for $C_{19}H_{28}N_3OS^+$ 346.1948; Found 346.1945.

***N*-Cyclohexyl-2-(dibenzylamino)-6-methyl-4*H*-3,1-benzothiazine-4-carboxamide (11i)**

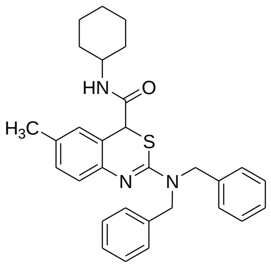

White solid (yield 0.377 g, 78%), mp 156-158 °C; ^{1}H NMR ($CDCl_3$, 600 MHz) δ (ppm) 7.32-7.25 (m, 10H, Ar-H), 7.14 (d, *J* = 7.8 Hz, 1H, Ar-H), 7.10 (d, *J* = 8.4 Hz, 1H, Ar-H), 6.98 (s, 1H, Ar-H), 5.47 (d, *J* = 7.8 Hz, 1H, NH), 5.10 (s, 2H, NCH_2), 4.57 (s, 1H, CH), 4.54 (d, *J* = 16.2 Hz, 2H, NCH_2), 3.69-3.65 (m, 1H, NCH), 2.34 (s, 3H, CH_3), 1.78-0.92 (m, 10H, $5CH_2$); ^{13}C NMR ($CDCl_3$, 150 MHz) δ (ppm) 168.4, 154.0, 143.1, 137.5, 133.3, 130.3, 128.5, 128.0, 127.7, 127.3, 125.5, 117.3, 51.1, 48.6, 48.0, 32.5, 32.4, 25.3, 24.5, 20.9. HRMS (ESI-TOF) *m/z* $[M+H]^+$ Calcd for $C_{30}H_{34}N_3OS^+$ 484.2417; Found 484.2411.

***N*-(*tert*-Butyl)-2-(dipropylamino)-6-methyl-4*H*-3,1-benzothiazine-4-carboxamide (11j)**

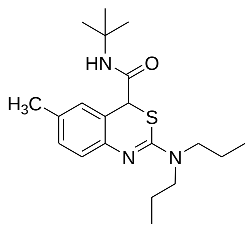

White solid (yield 0.271 g, 75%), mp 156-157 °C; ^{1}H NMR ($CDCl_3$, 600 MHz) δ (ppm) 7.09 (d, *J* = 7.8 Hz, 1H, Ar-H), 7.02 (d, *J* = 7.8 Hz, 1H, Ar-H), 6.93 (s, 1H, Ar-H), 5.50 (s, 1H, NH), 4.41 (s, 1H, CH), 3.67-3.62 (m, 2H, NCH_2), 3.40-3.36 (m, 2H, NCH_2), 2.31 (s, 3H, CH_3), 1.69-1.62 (m, 4H, $2CH_2$), 1.20 (s, 9H, $3CH_3$), 0.93 (t, *J* = 7.2 Hz, 6H, $2CH_3$); ^{13}C NMR ($CDCl_3$, 150 MHz) δ (ppm) 168.6, 152.6, 143.2, 132.5, 130.0, 127.9, 125.1, 117.6, 51.3, 51.1, 48.6, 28.3, 22.0, 20.8, 11.3. HRMS (ESI-TOF) *m/z* $[M+H]^+$ Calcd for $C_{20}H_{32}N_3OS^+$ 362.2261; Found 362.2258.

7-Chloro-*N*-cyclohexyl-2-(diethylamino)-4*H*-3,1-benzothiazine-4-carboxamide (11k)

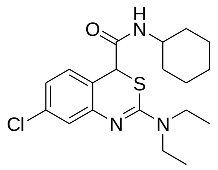

White solid (yield 0.315 g, 83%), mp 162-164 °C; ^{1}H NMR ($CDCl_3$, 600 MHz) δ (ppm) 7.15 (s, 1H, Ar-H), 7.06 (d, *J* = 8.4 Hz, 1H, Ar-H), 6.98 (d, *J* = 7.8 Hz, 1H, Ar-H), 5.49 (d, *J* = 6.0 Hz, 1H, NH), 4.53 (s, 1H, CH), 3.74-3.48 (m, 5H, $2\times NCH_2$ and NCH), 1.74-1.29 (m, 7H, $7/10\times 5CH_2$), 1.21 (t, *J* = 7.2 Hz, 6H, $2CH_3$), 1.12-0.94 (m, 3H, $3/10\times 5CH_2$); ^{13}C NMR ($CDCl_3$, 150 MHz) δ (ppm) 168.0, 153.5, 147.1, 134.7, 128.4, 125.0, 122.7, 116.0, 48.4, 47.1, 43.9, 32.4, 32.3, 25.3, 24.3, 14.0. HRMS (ESI-TOF) *m/z* $[M+H]^+$ Calcd for $C_{19}H_{27}ClN_3OS^+$ 380.1558; Found 380.1554.

***N*-(*tert*-Butyl)-7-chloro-2-(dicyclohexylamino)-4*H*-3,1-benzothiazine-4-carboxamide (11l)**

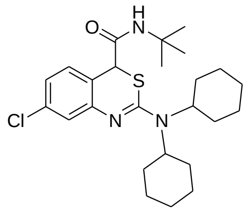

White solid (yield 0.249 g, 54%), mp 118-120 °C; ^{1}H NMR ($CDCl_3$, 600 MHz) δ (ppm) 7.12 (s, 1H, Ar-H), 7.05 (d, *J* = 7.8 Hz, 1H, Ar-H), 6.97 (d, *J* = 7.8 Hz, 1H, Ar-H), 5.58 (s, 1H, NH), 4.41 (s, 1H, CH), 3.80-3.67 (m, 2H, 2CH), 2.21-1.15 (m, 29H, $10CH_2$ and $3CH_3$); ^{13}C NMR ($CDCl_3$, 150 MHz) δ (ppm) 168.2, 152.5, 146.6, 134.4, 128.4, 124.6, 122.6, 116.6, 59.1, 51.5, 48.3, 32.0, 30.7, 28.4, 26.3, 26.2, 25.4. HRMS (ESI-TOF) *m/z* $[M+H]^+$ Calcd for $C_{25}H_{37}ClN_3OS^+$ 462.2340; Found 462.2336.

***N*-Cyclohexyl-2-(diisopropylamino)-6-methyl-4*H*-3,1-benzothiazine-4-carboxamide (11m)**

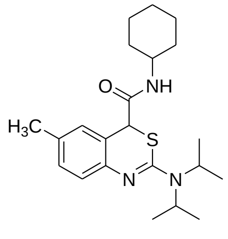

White solid (yield 0.186 g, 48%), mp 168-169 °C; ^{1}H NMR ($CDCl_3$, 600 MHz) δ (ppm) 7.09 (d, *J* = 7.8 Hz, 1H, Ar-H), 7.03 (d, *J* = 8.4 Hz, 1H, Ar-H), 6.92 (s, 1H, Ar-H), 5.61 (d, *J* = 6.6 Hz, 1H, NH), 4.48 (s, 1H, CH), 4.23-4.18 (m, 2H, 2NCH), 3.69-3.64 (m, 1H, NCH), 2.31 (s, 3H, CH_3), 1.78-0.94 (m, 22H, $5CH_2$ and $4CH_3$); ^{13}C NMR ($CDCl_3$, 150 MHz) δ (ppm) 168.8, 150.9, 143.0, 132.6, 130.0, 127.8, 125.0, 117.5, 49.0, 48.2, 48.0, 32.5, 32.3, 25.4, 24.3, 22.1, 20.8. HRMS (ESI-TOF) *m/z* $[M+H]^+$ Calcd for $C_{22}H_{34}N_3OS^+$ 388.2417; Found 388.2412.

***N*-Cyclohexyl-2-(methyl(phenyl)amino)-4*H*-3,1-benzothiazine-4-carboxamide (11n)**

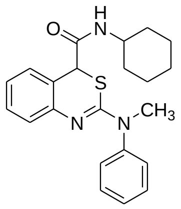

White solid (yield 0.212 g, 56%), mp 194-196 °C; ^{1}H NMR ($CDCl_3$, 600 MHz) δ (ppm) 7.41 (t, *J* = 7.8 Hz, 2H, Ar-H), 7.34 (t, *J* = 7.8 Hz, 2H, Ar-H), 7.25-7.10 (m, 3H, Ar-H), 7.17 (d, *J* = 7.2 Hz, 1H, Ar-H), 7.09 (t, *J* = 7.2 Hz, 1H, Ar-H), 5.64 (d, *J* = 6.0 Hz, 1H, NH), 4.47 (s, 1H, CH), 3.70-3.66 (m, 1H, NCH), 3.56 (s, 3H, NCH_3), 1.81-0.99 (m, 10H, $5CH_2$); ^{13}C NMR ($CDCl_3$, 150 MHz) δ (ppm) 167.9, 153.2, 144.9, 144.0, 129.3, 128.6, 127.8, 127.7, 127.6, 125.5, 123.9, 118.2, 48.3, 48.0, 39.7, 32.4, 25.4, 24.3. HRMS (ESI-TOF) *m/z* $[M+H]^+$ Calcd for $C_{22}H_{26}N_3OS^+$ 380.1791; Found 380.1785.

***N*-Cyclohexyl-6-methyl-2-(methyl(phenyl)amino)-4*H*-3,1-benzothiazine-4-carboxamide (11o)**

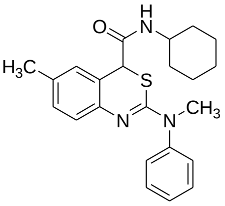

White solid (yield 0.201 g, 51%), mp 207-208 °C; ^{1}H NMR ($CDCl_3$, 600 MHz) δ (ppm) 7.40 (t, *J* = 7.2 Hz, 2H, Ar-H), 7.32 (t, *J* = 7.2 Hz, 1H, Ar-H), 7.22-7.14 (m, 4H, Ar-H), 6.97 (s, 1H, Ar-H), 5.69 (d, *J* = 5.4 Hz, 1H, NH), 4.41 (s, 1H, CH), 3.70-3.66 (m, 1H, NCH), 3.54 (s, 3H, NCH_3), 2.33 (s, 3H, CH_3), 1.81-1.01 (m, 10H, $5CH_2$); ^{13}C NMR ($CDCl_3$, 150 MHz) δ (ppm) 168.0, 152.5, 144.1, 142.5, 133.6, 130.1, 129.3, 128.3, 127.6, 127.4, 125.3, 117.9, 48.3, 48.1, 39.7, 32.4, 25.4, 24.3, 20.8. HRMS (ESI-TOF) *m/z* $[M+H]^+$ Calcd for $C_{23}H_{28}N_3OS^+$ 394.1948; Found 394.1942.

1.5 Reference

1. He, P.; Wu, J.; Nie, Y. B.; Ding, M. W. *Tetrahedron* **2009**, *65*, 8563-8570.

2. Copies of ^{1}H and ^{13}C NMR spectrum of compound 4, 8 and 11

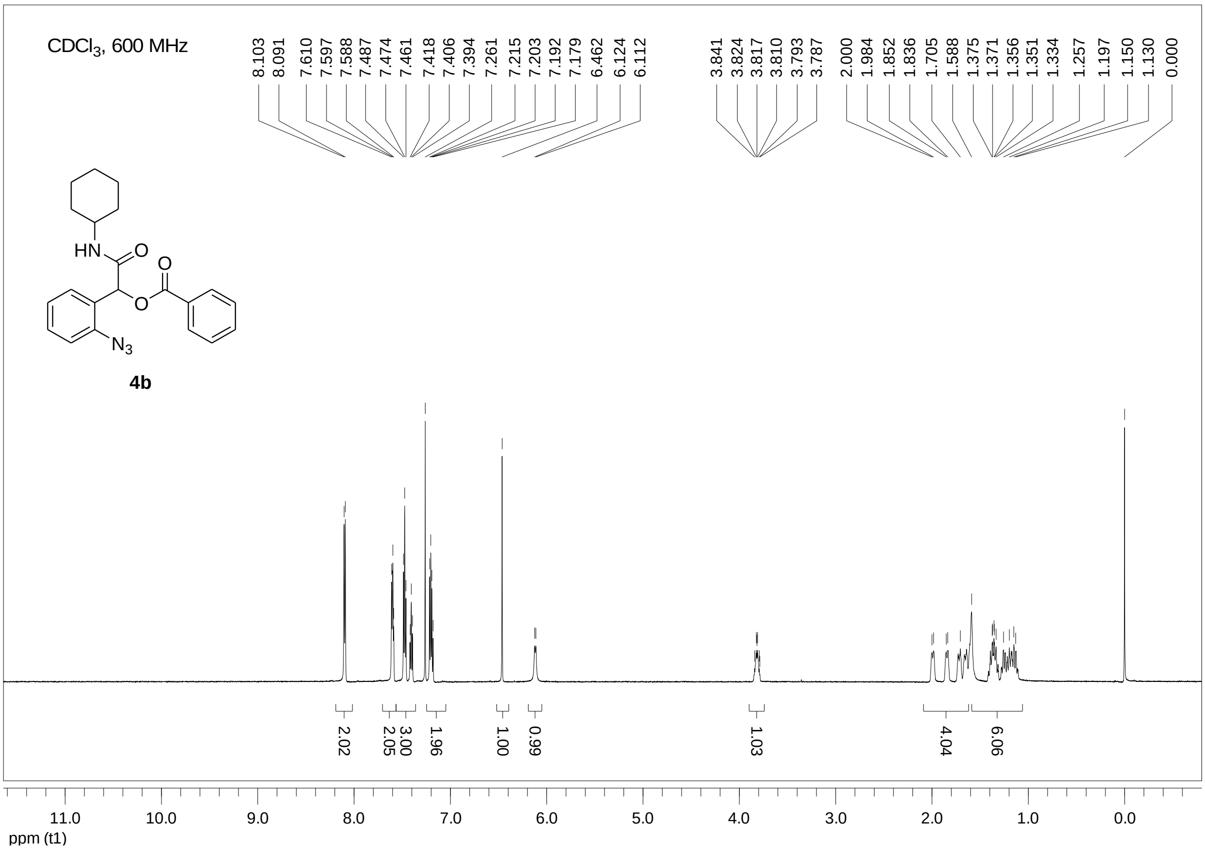

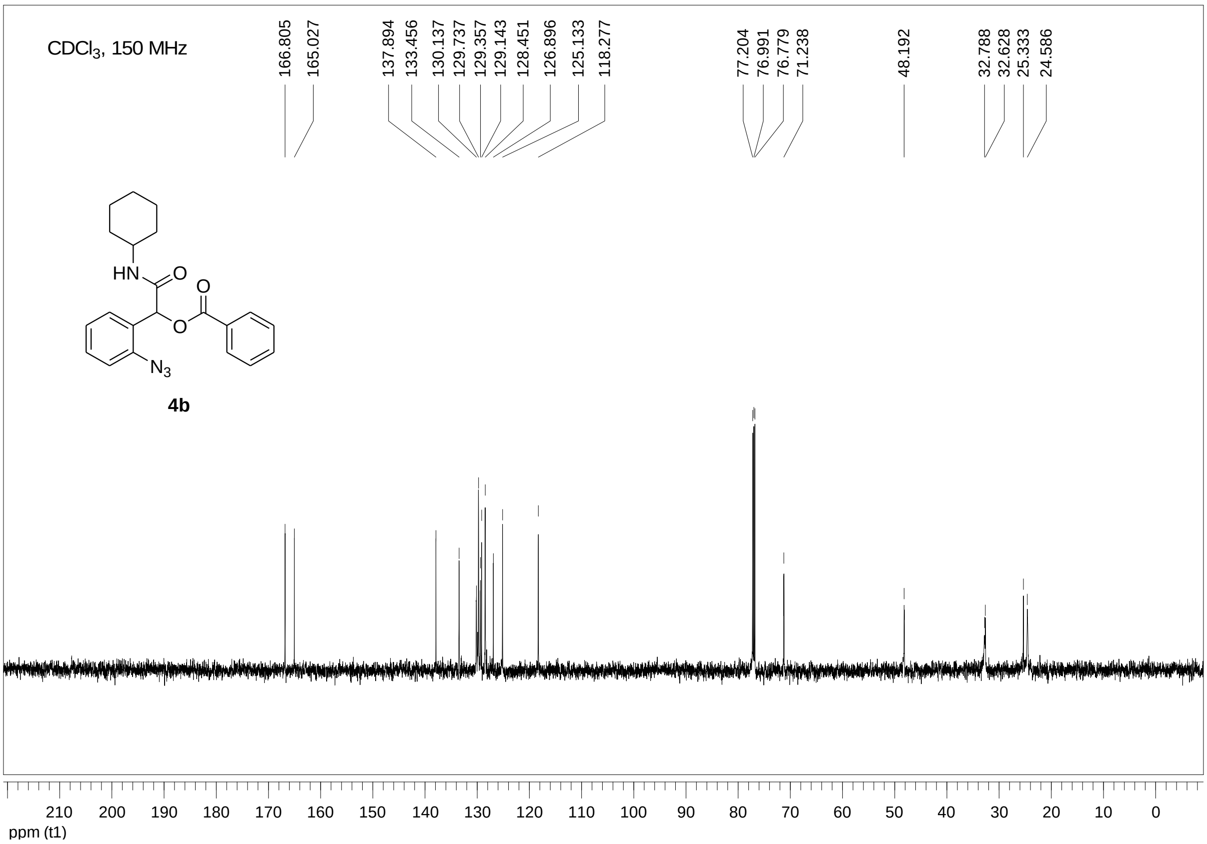

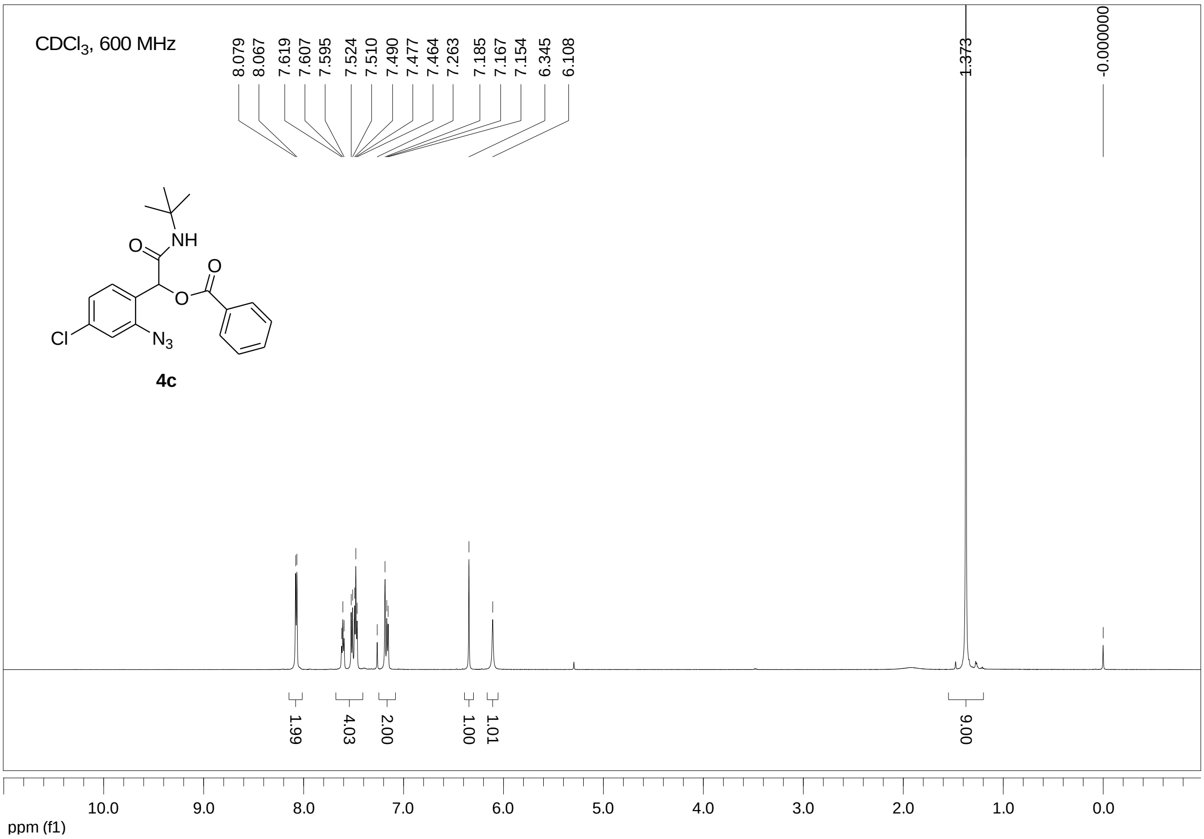
CDCl3, 600 MHz
8.079
8.067
7.619
7.607
7.595
7.524
7.510
7.490
7.477
7.464
7.263
7.185
7.167
7.154
6.345
6.108
1.373
-0.000000
NH
O
O
O
Cl
N3
4c
1.99
4.03
2.00
1.00
1.01
9.00
10.0
9.0
8.0
7.0
6.0
5.0
4.0
3.0
2.0
1.0
0.0
ppm (f1)

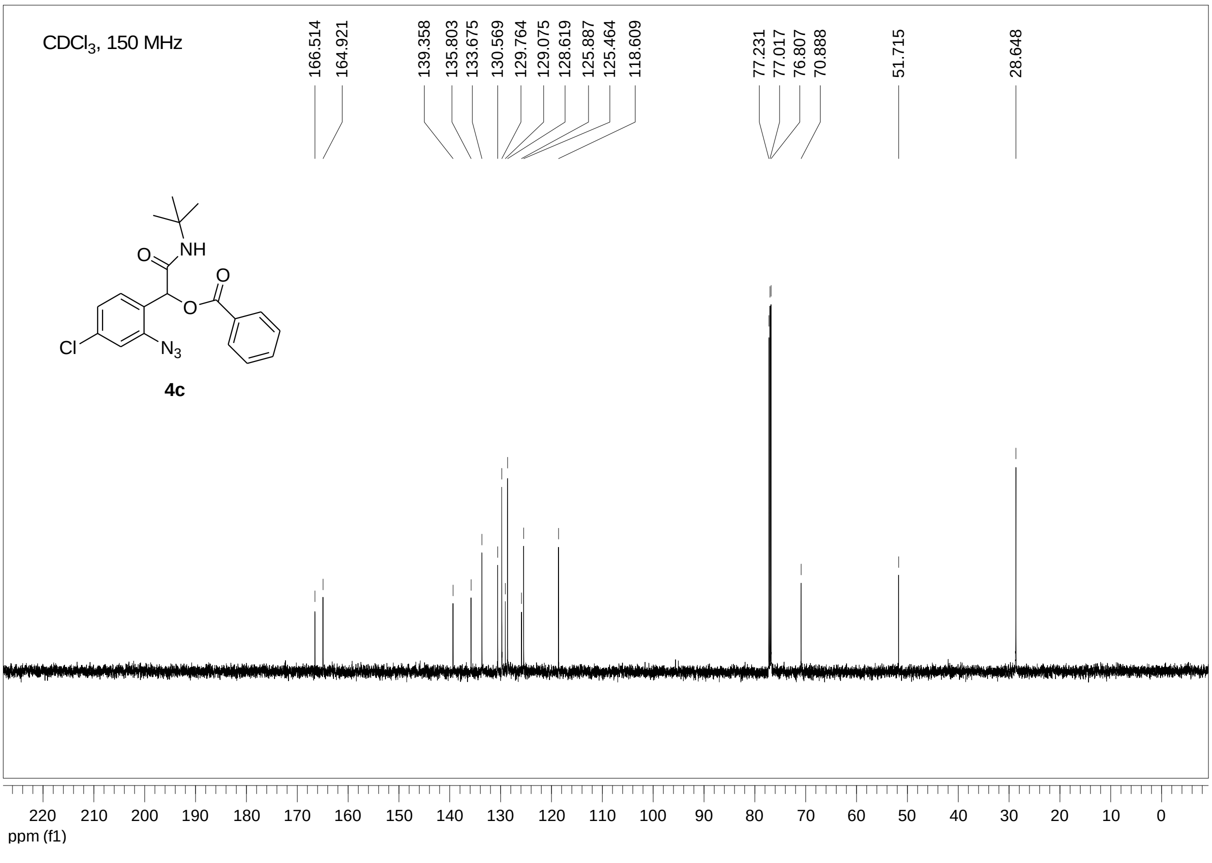
CDCl3, 150 MHz
166.514
164.921
139.358
135.803
133.675
130.569
129.764
129.075
128.619
125.887
125.464
118.609
77.231
77.017
76.807
70.888
51.715
28.648
NH
O
O
O
Cl
N3
4c
220
210
200
190
180
170
160
150
140
130
120
110
100
90
80
70
60
50
40
30
20
10
0
ppm (f1)

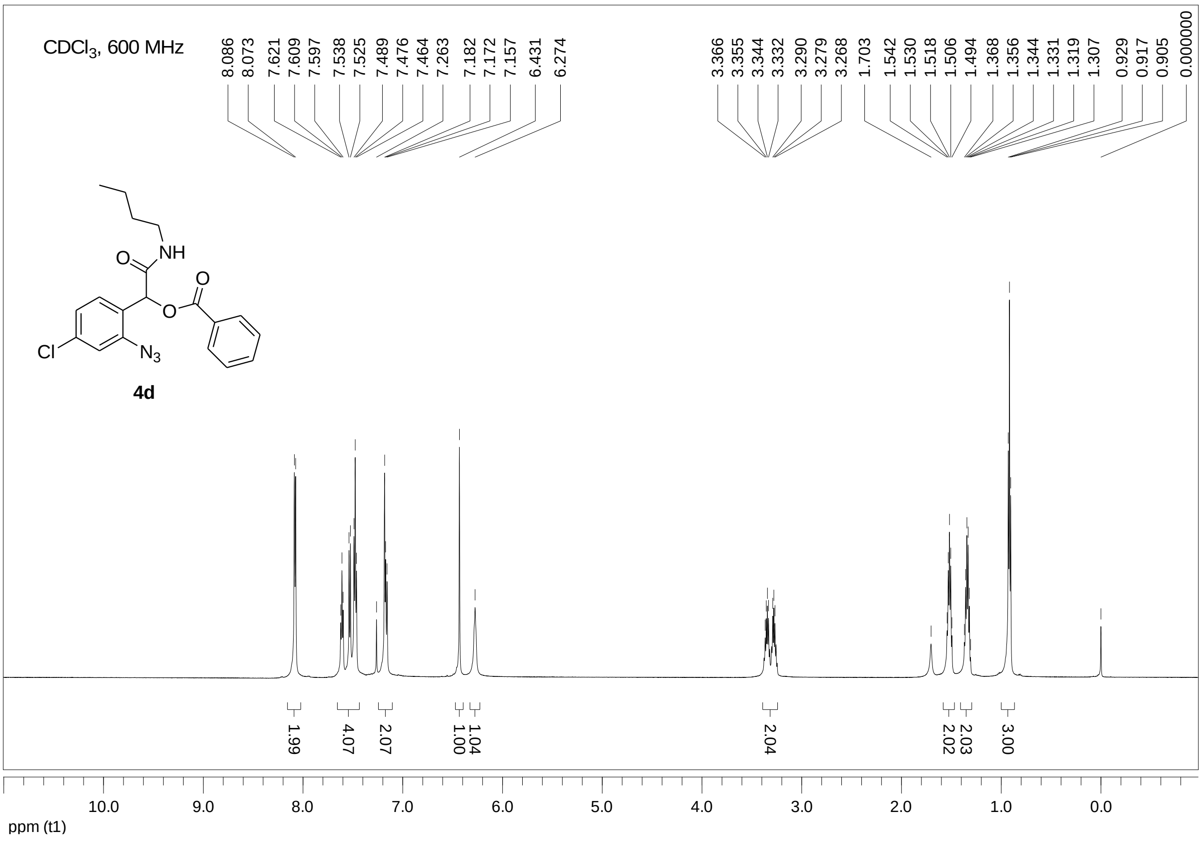
CDCl3, 600 MHz
8.086
8.073
7.621
7.609
7.597
7.538
7.525
7.489
7.476
7.464
7.263
7.182
7.172
7.157
6.431
6.274
3.366
3.355
3.344
3.332
3.290
3.279
3.268
1.703
1.542
1.530
1.518
1.506
1.494
1.368
1.356
1.344
1.331
1.319
1.307
0.929
0.917
0.905
0.000000
NH
O
O
O
Cl
N3
4d
1.99
4.07
2.07
1.00
1.04
2.04
2.02
2.03
3.00
10.0
9.0
8.0
7.0
6.0
5.0
4.0
3.0
2.0
1.0
0.0
ppm (t1)

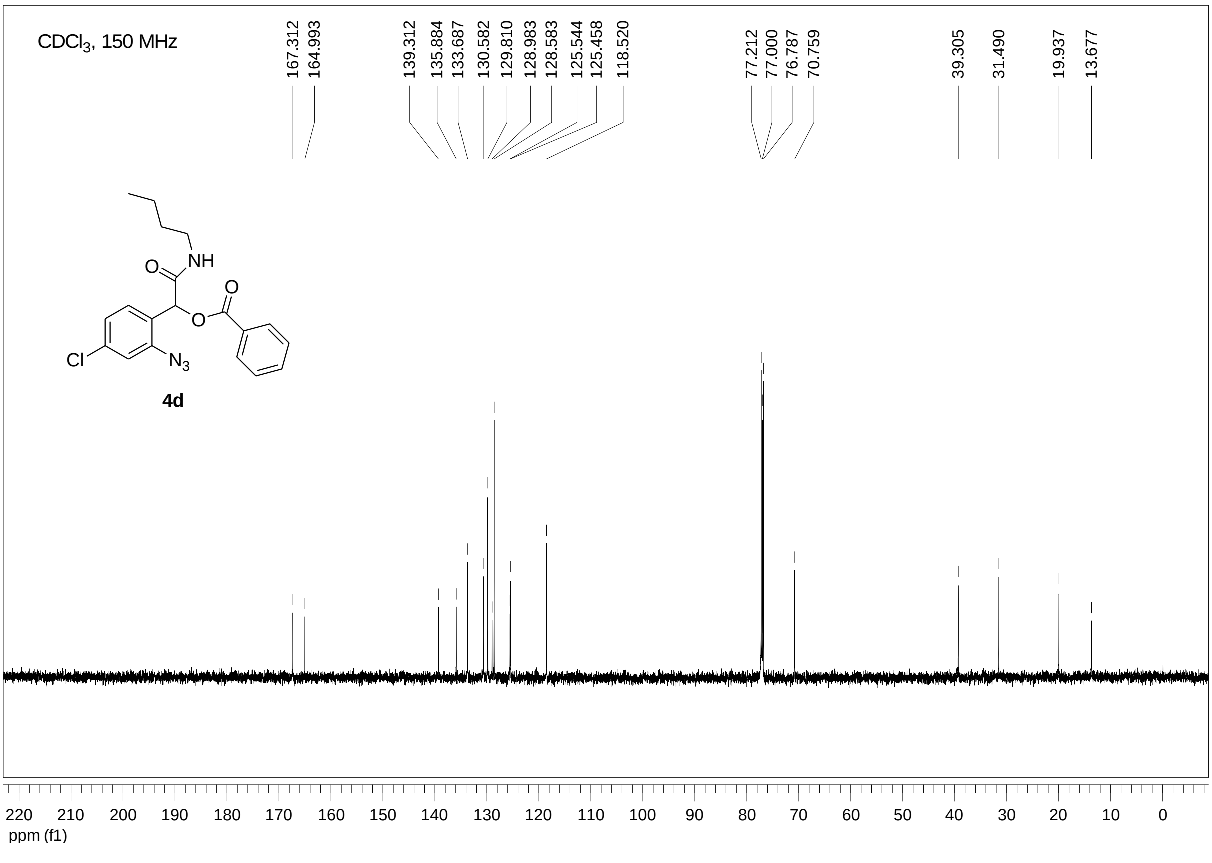
CDCl3, 150 MHz
167.312
164.993
139.312
135.884
133.687
130.582
129.810
128.983
128.583
125.544
125.458
118.520
77.212
77.000
76.787
70.759
39.305
31.490
19.937
13.677
NH
O
O
O
Cl
N3
4d
220
210
200
190
180
170
160
150
140
130
120
110
100
90
80
70
60
50
40
30
20
10
0
ppm (f1)

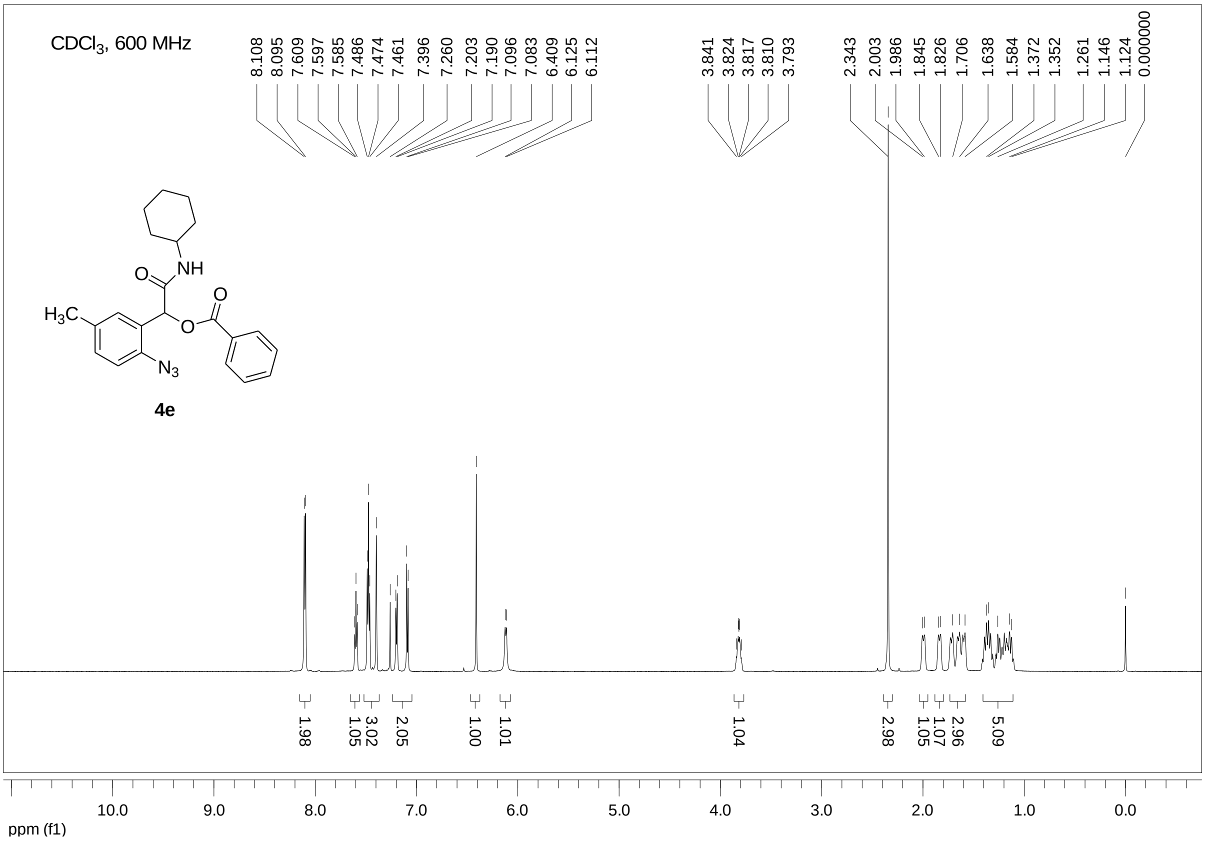
CDCl3, 600 MHz
8.108 8.095 7.609 7.597 7.585 7.486 7.474 7.461 7.396 7.260 7.203 7.190 7.096 7.083 6.409 6.125 6.112
3.841 3.824 3.817 3.810 3.793
2.343 2.003 1.986 1.845 1.826 1.706 1.638 1.584 1.372 1.352 1.261 1.146 1.124 0.000000
NH
O
H3C
N3
4e
1.98 1.05 3.02 2.05 1.00 1.01 1.04 2.98 1.05 1.07 2.96 5.09
10.0 9.0 8.0 7.0 6.0 5.0 4.0 3.0 2.0 1.0 0.0
ppm (f1)

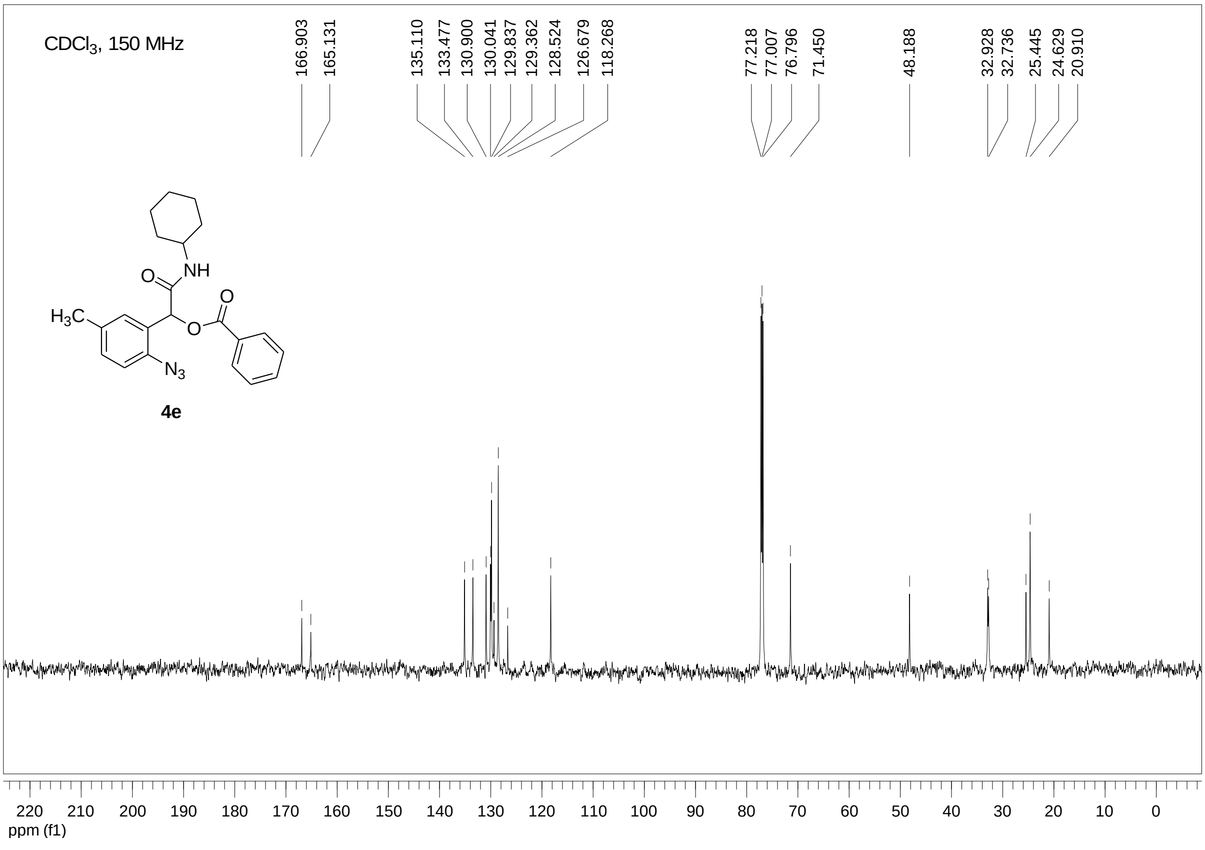
CDCl3, 150 MHz
166.903 165.131
135.110 133.477 130.900 130.041 129.837 129.362 128.524 126.679 118.268
77.218 77.007 76.796 71.450
48.188
32.928 32.736 25.445 24.629 20.910
NH
O
H3C
N3
4e
220 210 200 190 180 170 160 150 140 130 120 110 100 90 80 70 60 50 40 30 20 10 0
ppm (f1)

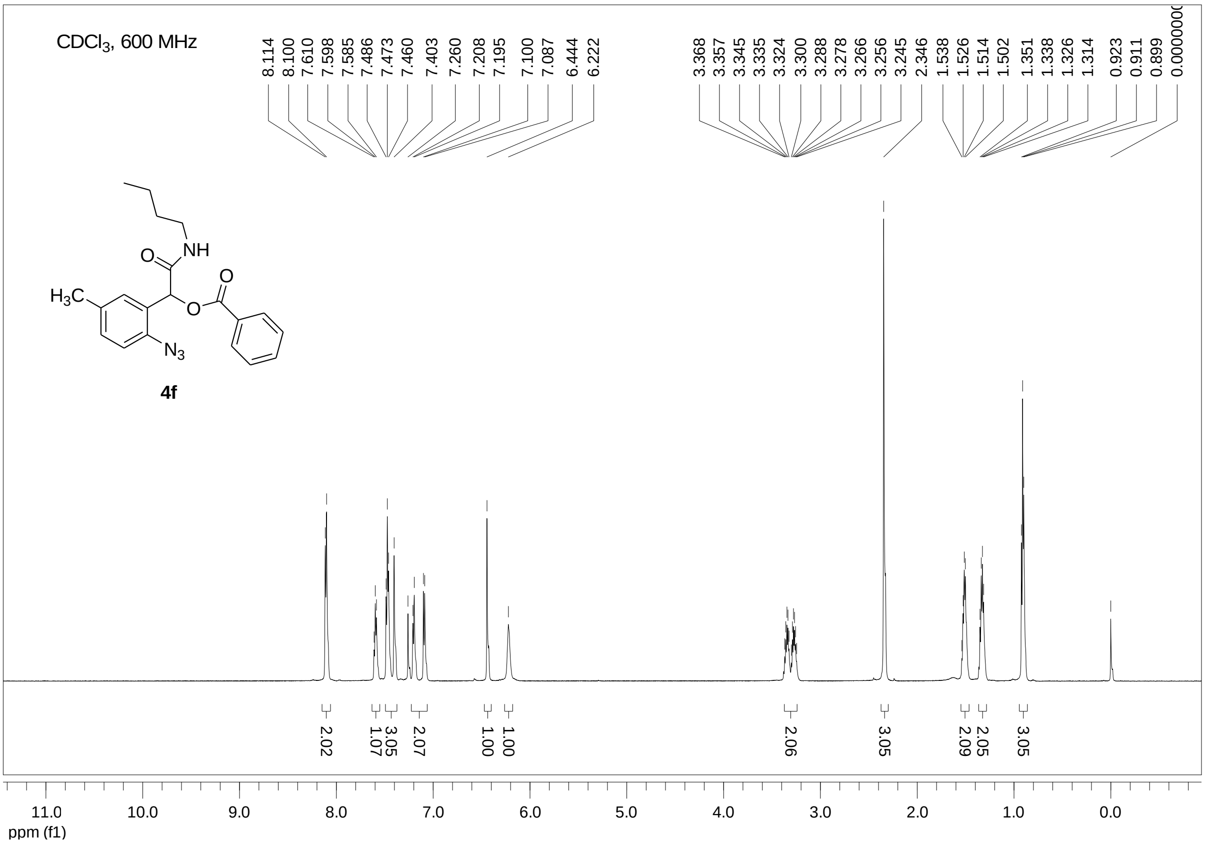
CDCl3, 600 MHz
8.114
8.100
7.610
7.598
7.585
7.486
7.473
7.460
7.403
7.260
7.208
7.195
7.100
7.087
6.444
6.222
3.368
3.357
3.345
3.335
3.324
3.300
3.288
3.278
3.266
3.256
3.245
2.346
1.538
1.526
1.514
1.502
1.351
1.338
1.326
1.314
0.923
0.911
0.899
0.0000000
O
NH
H3C
O
O
N3
4f
2.02
1.07
3.05
2.07
1.00
1.00
2.06
3.05
2.09
2.05
3.05
11.0
10.0
9.0
8.0
7.0
6.0
5.0
4.0
3.0
2.0
1.0
0.0
ppm (f1)

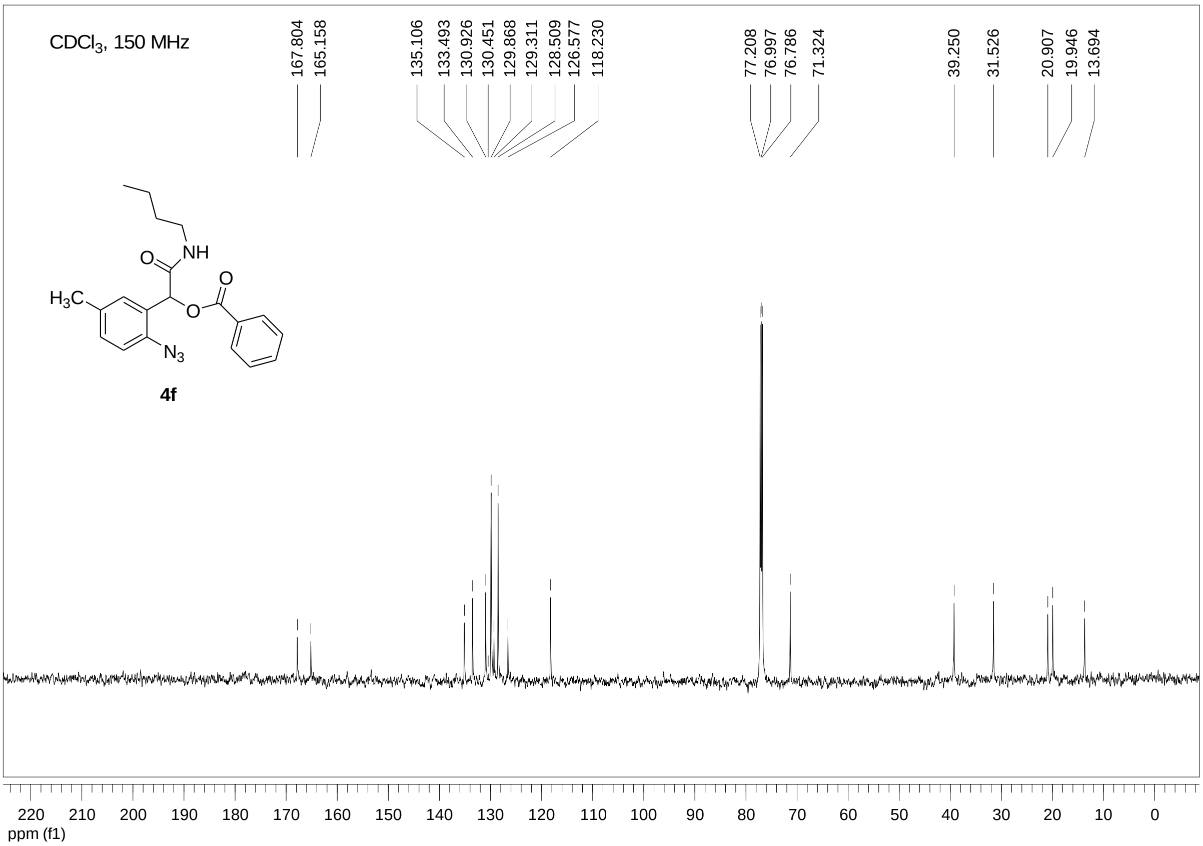
CDCl3, 150 MHz
167.804
165.158
135.106
133.493
130.926
130.451
129.868
129.311
128.509
126.577
118.230
77.208
76.997
76.786
71.324
39.250
31.526
20.907
19.946
13.694
O
NH
H3C
O
O
N3
4f
220
210
200
190
180
170
160
150
140
130
120
110
100
90
80
70
60
50
40
30
20
10
0
ppm (f1)

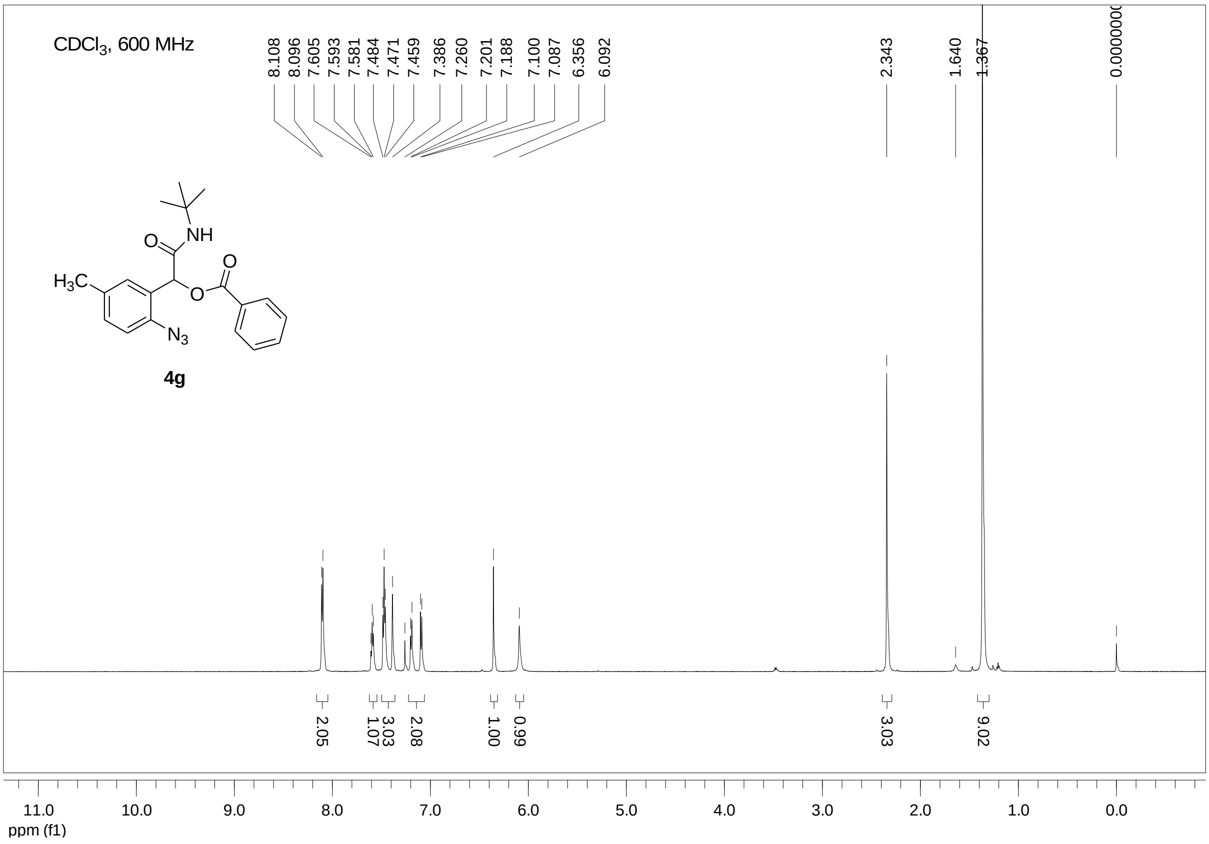
CDCl$_3$, 600 MHz
8.108
8.096
7.605
7.593
7.581
7.484
7.471
7.459
7.386
7.260
7.201
7.188
7.100
7.087
6.356
6.092
2.343
1.640
1.367
0.000000
2.05
1.07
3.03
2.08
1.00
0.99
3.03
9.02
ppm (f1)
4g

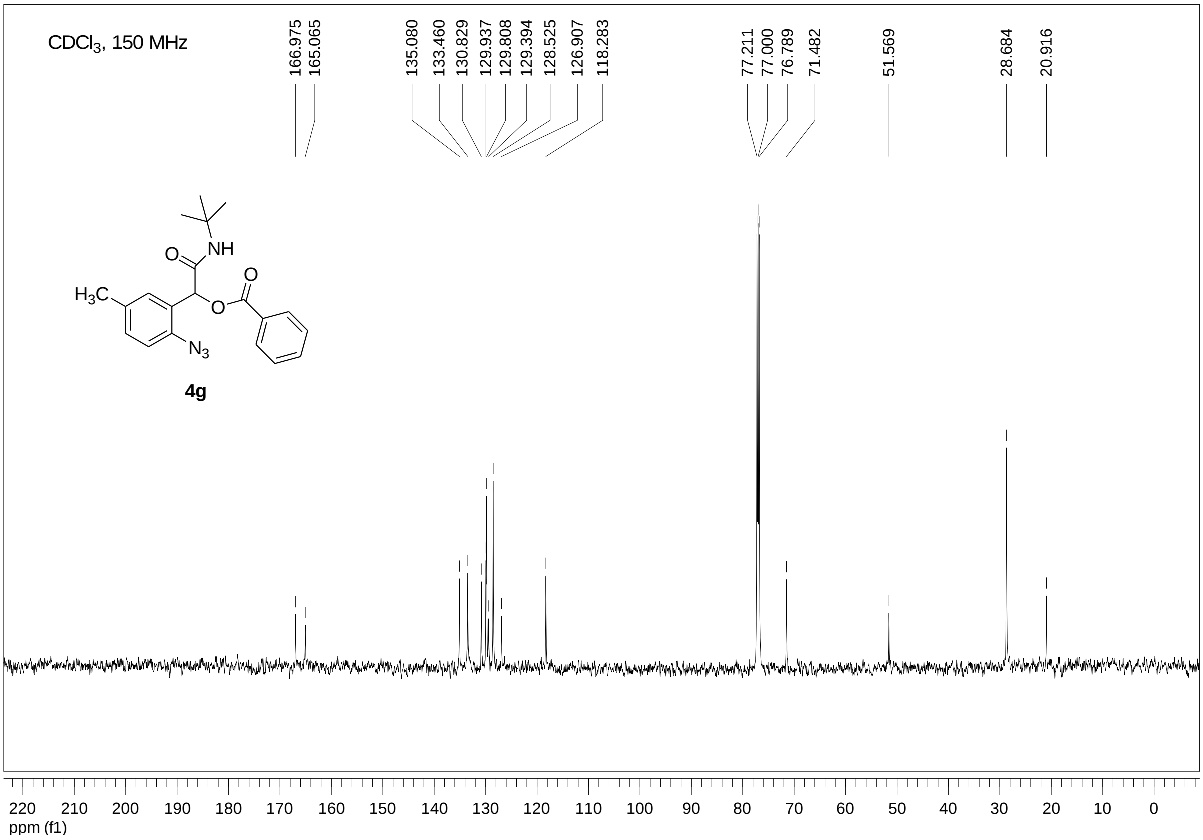
CDCl$_3$, 150 MHz
166.975
165.065
135.080
133.460
130.829
129.937
129.808
129.394
128.525
126.907
118.283
77.211
77.000
76.789
71.482
51.569
28.684
20.916
ppm (f1)
4g

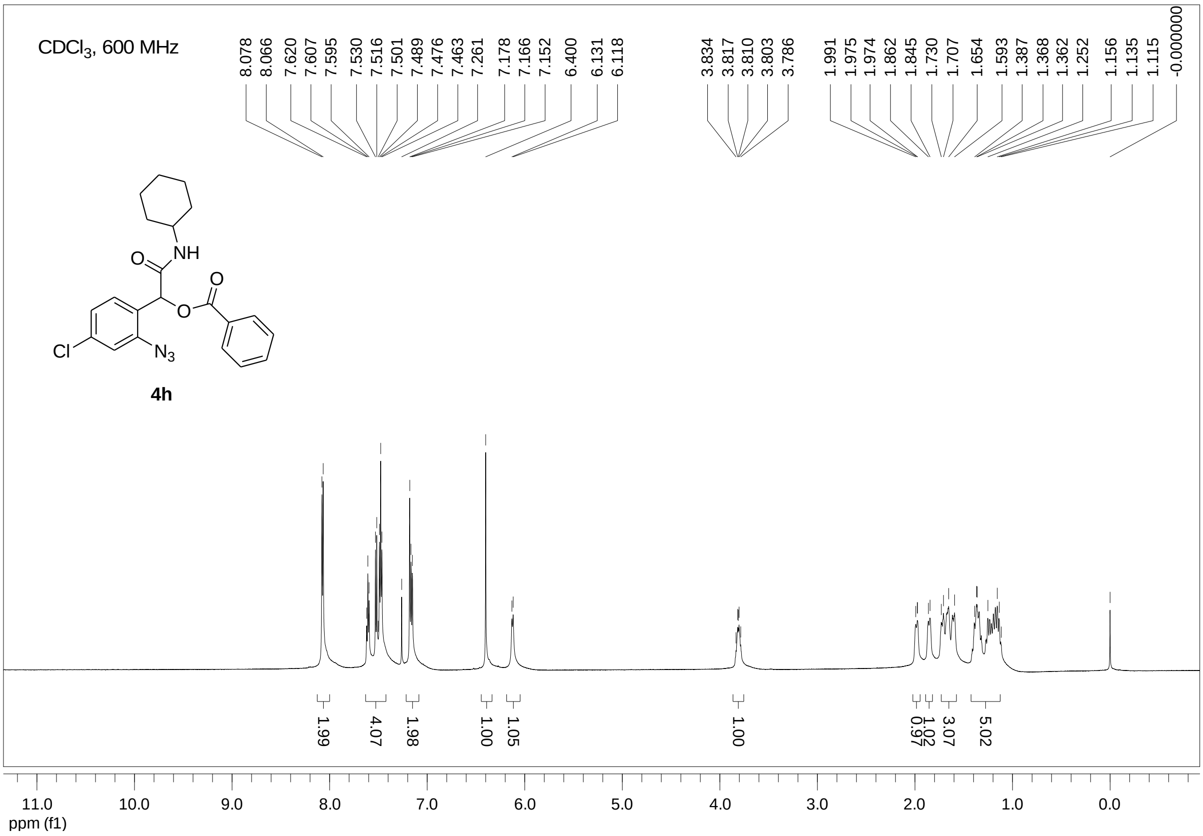
CDCl3, 600 MHz
8.078 8.066 7.620 7.607 7.595 7.530 7.516 7.501 7.489 7.476 7.463 7.261 7.178 7.166 7.152 6.400 6.131 6.118
3.834 3.817 3.810 3.803 3.786
1.991 1.975 1.974 1.862 1.845 1.730 1.707 1.654 1.593 1.387 1.368 1.362 1.252 1.156 1.135 1.115 -0.000000
NH
O
Cl
N3
4h
1.99 4.07 1.98 1.00 1.05 1.00 0.97 1.02 3.07 5.02
11.0 10.0 9.0 8.0 7.0 6.0 5.0 4.0 3.0 2.0 1.0 0.0
ppm (f1)

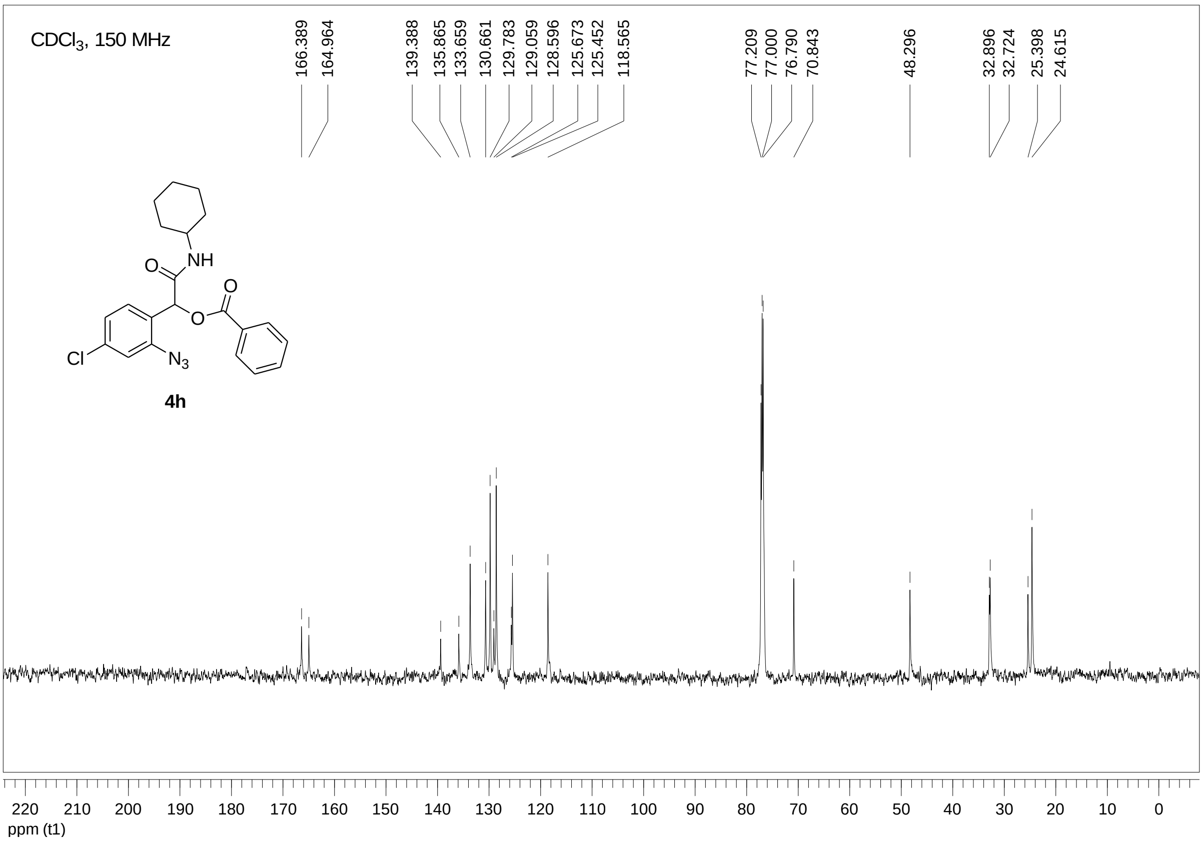
CDCl3, 150 MHz
166.389 164.964
139.388 135.865 133.659 130.661 129.783 129.059 128.596 125.673 125.452 118.565
77.209 77.000 76.790 70.843
48.296
32.896 32.724 25.398 24.615
NH
O
Cl
N3
4h
220 210 200 190 180 170 160 150 140 130 120 110 100 90 80 70 60 50 40 30 20 10 0
ppm (t1)

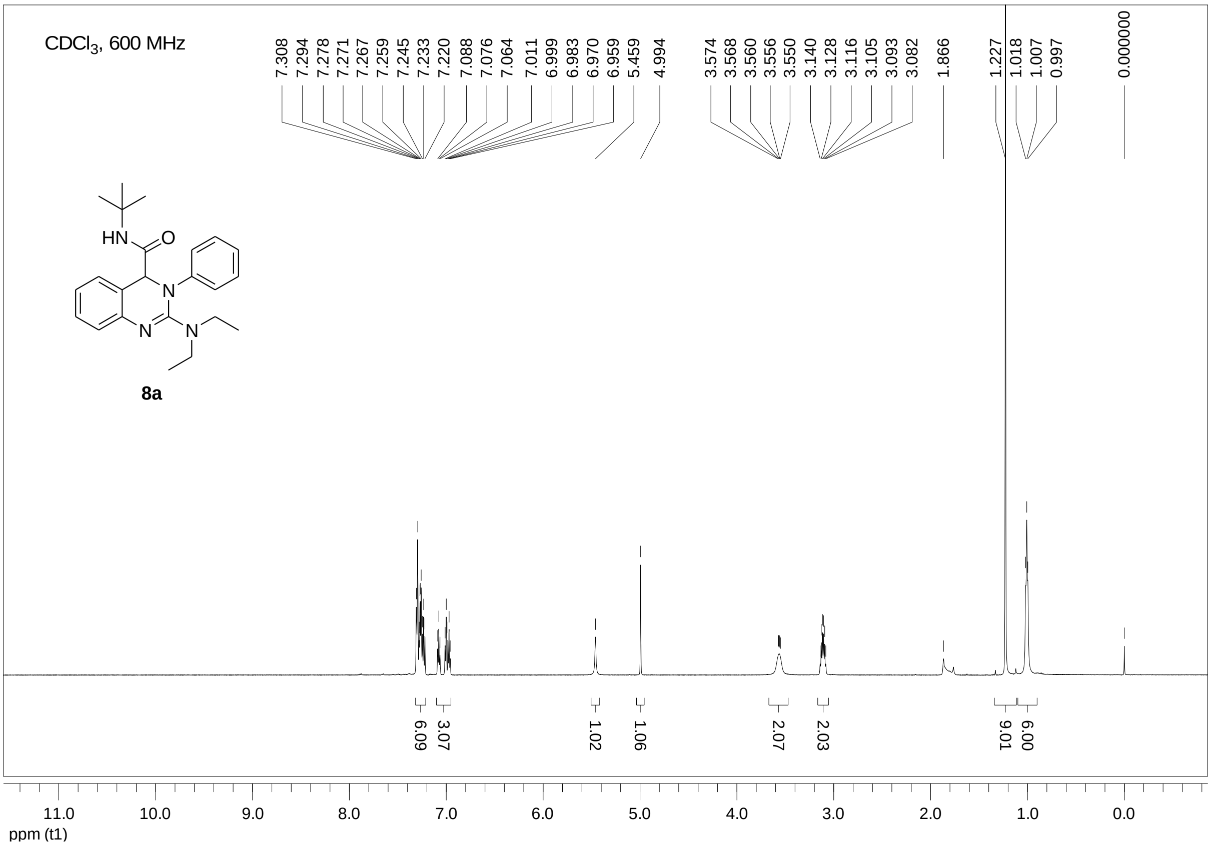
CDCl3, 600 MHz
7.308
7.294
7.278
7.271
7.267
7.259
7.245
7.233
7.220
7.088
7.076
7.064
7.011
6.999
6.983
6.970
6.959
5.459
4.994
3.574
3.568
3.560
3.556
3.550
3.140
3.128
3.116
3.105
3.093
3.082
1.866
1.227
1.018
1.007
0.997
0.000000
HN
O
N
N
N
8a
6.09
3.07
1.02
1.06
2.07
2.03
9.01
6.00
11.0
10.0
9.0
8.0
7.0
6.0
5.0
4.0
3.0
2.0
1.0
0.0
ppm (t1)

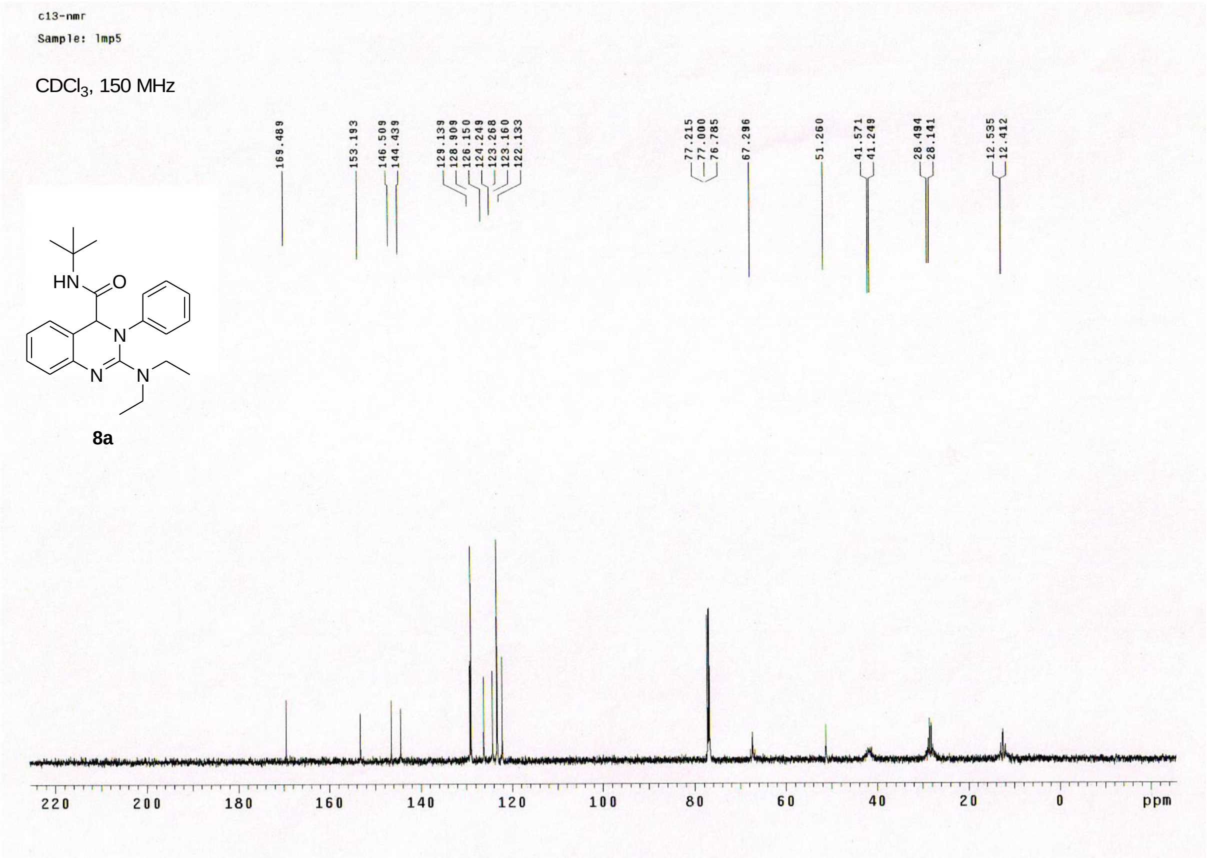
c13-nmr
Sample: lmp5
CDCl3, 150 MHz
169.489
153.193
146.509
144.439
129.139
128.909
126.150
124.249
123.268
123.160
122.133
77.215
77.000
76.785
67.296
51.260
41.571
41.249
28.494
28.141
12.535
12.412
HN
O
N
N
N
8a
220
200
180
160
140
120
100
80
60
40
20
0
ppm

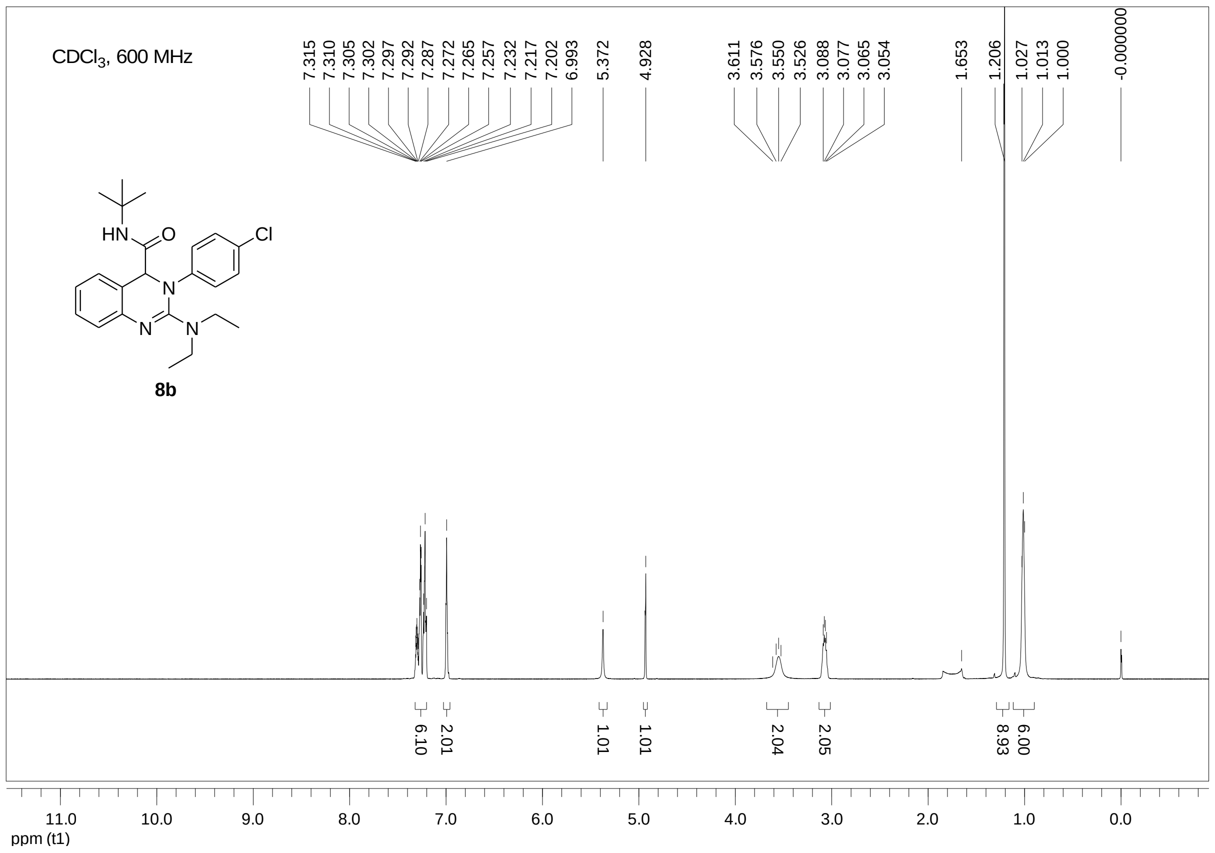
CDCl3, 600 MHz
7.315
7.310
7.305
7.302
7.297
7.292
7.287
7.272
7.265
7.257
7.232
7.217
7.202
6.993
5.372
4.928
3.611
3.576
3.550
3.526
3.088
3.077
3.065
3.054
1.653
1.206
1.027
1.013
1.000
-0.000000
HN
O
Cl
N
N
N
8b
6.10
2.01
1.01
1.01
2.04
2.05
8.93
6.00
11.0
10.0
9.0
8.0
7.0
6.0
5.0
4.0
3.0
2.0
1.0
0.0
ppm (t1)

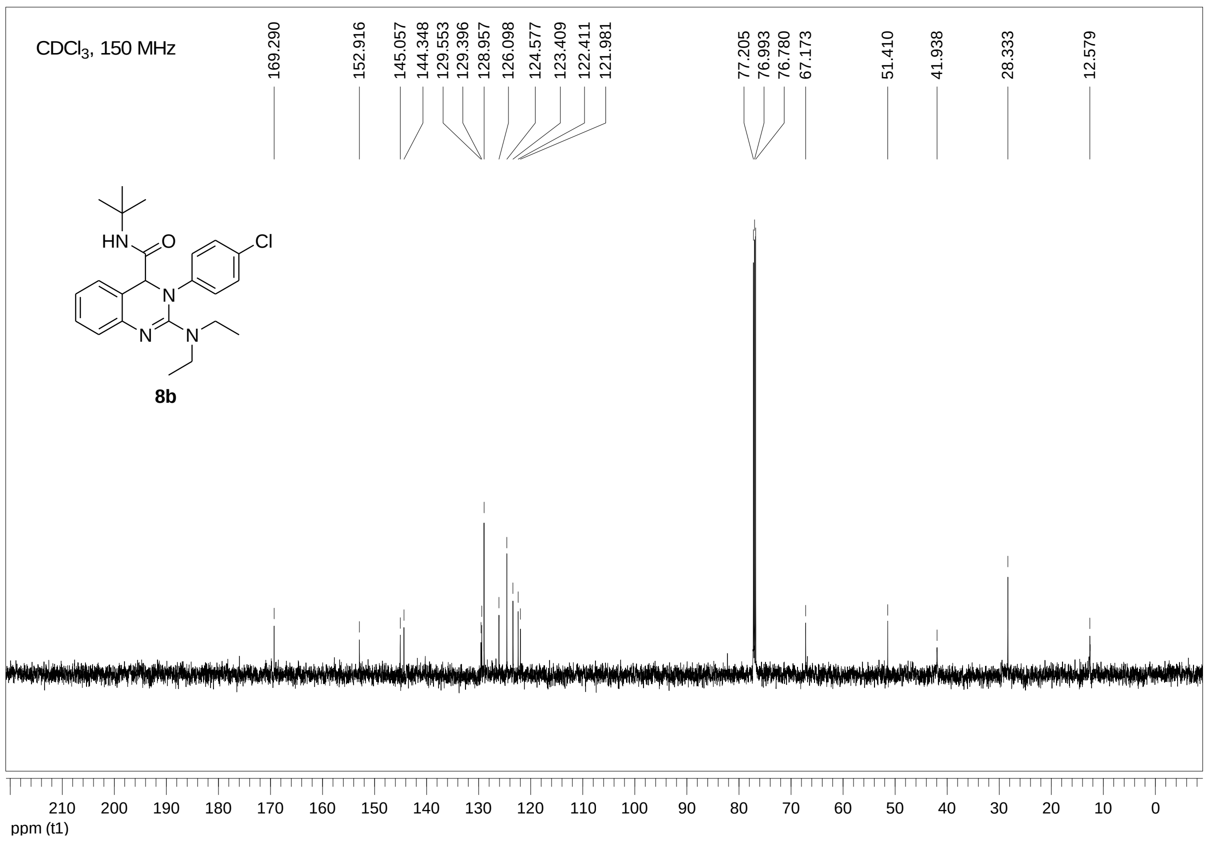
CDCl3, 150 MHz
169.290
152.916
145.057
144.348
129.553
129.396
128.957
126.098
124.577
123.409
122.411
121.981
77.205
76.993
76.780
67.173
51.410
41.938
28.333
12.579
HN
O
Cl
N
N
N
8b
210
200
190
180
170
160
150
140
130
120
110
100
90
80
70
60
50
40
30
20
10
0
ppm (t1)

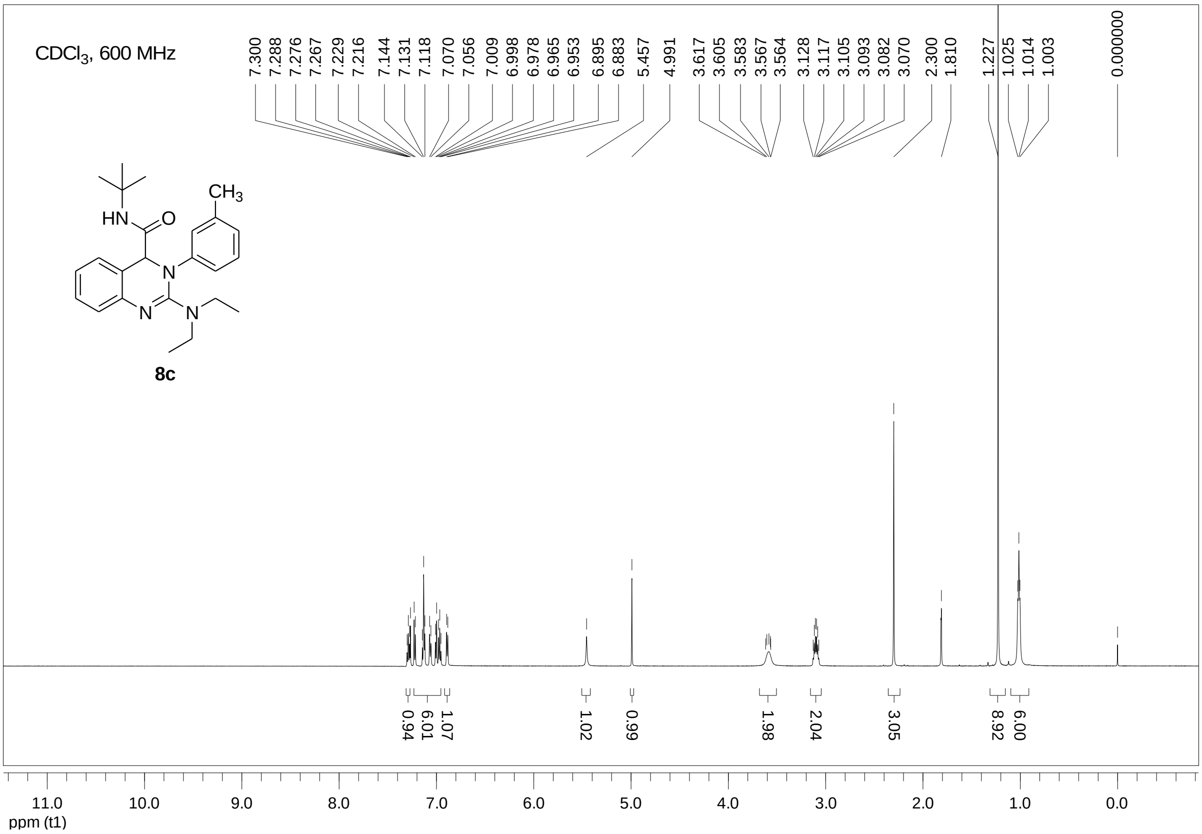
CDCl$_3$, 600 MHz
7.300 7.288 7.276 7.267 7.229 7.216 7.144 7.131 7.118 7.070 7.056 7.009 6.998 6.978 6.965 6.953 6.895 6.883 5.457 4.991 3.617 3.605 3.583 3.567 3.564 3.128 3.117 3.105 3.093 3.082 3.070 2.300 1.810 1.227 1.025 1.014 1.003 0.000000
CH$_3$
HN
O
N
N
N
8c
0.94 6.01 1.07 1.02 0.99 1.98 2.04 3.05 8.92 6.00
11.0 10.0 9.0 8.0 7.0 6.0 5.0 4.0 3.0 2.0 1.0 0.0
ppm (t1)

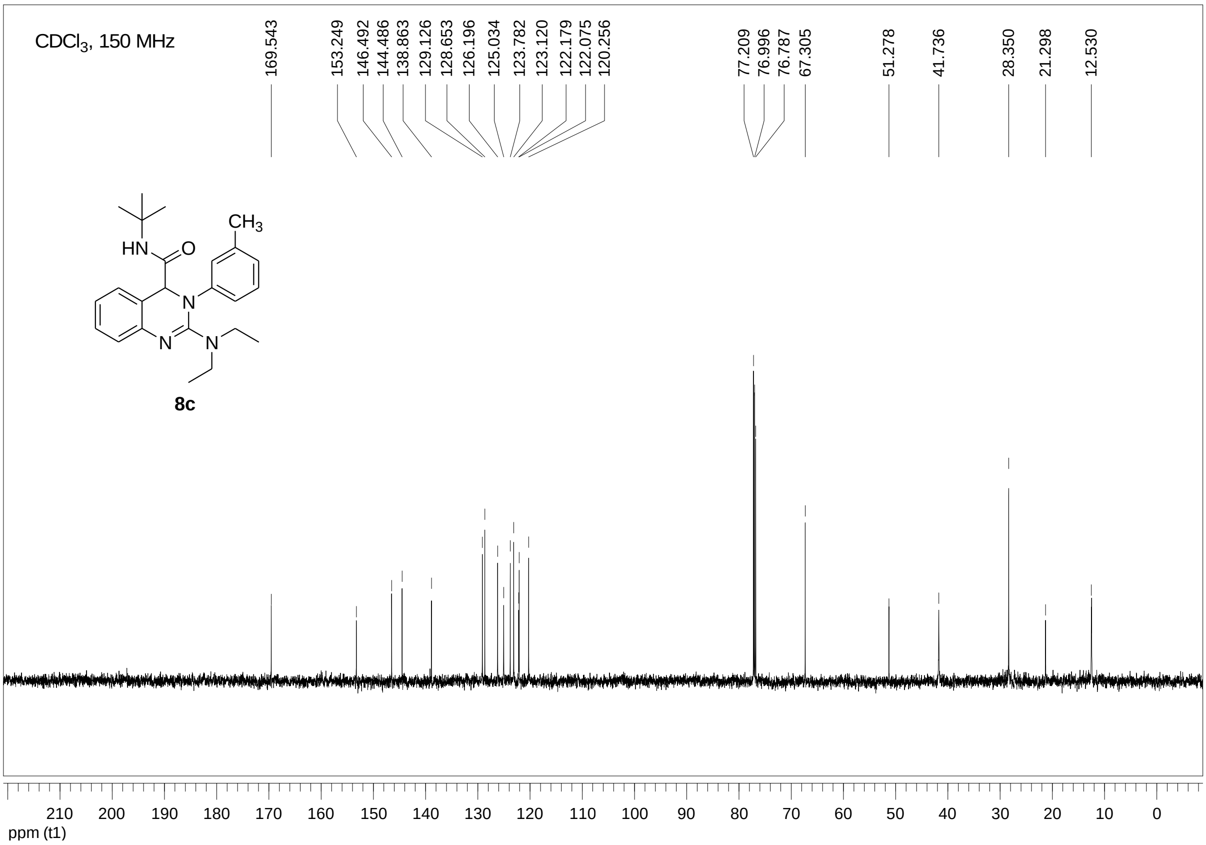
CDCl$_3$, 150 MHz
169.543 153.249 146.492 144.486 138.863 129.126 128.653 126.196 125.034 123.782 123.120 122.179 122.075 120.256 77.209 76.996 76.787 67.305 51.278 41.736 28.350 21.298 12.530
CH$_3$
HN
O
N
N
N
8c
210 200 190 180 170 160 150 140 130 120 110 100 90 80 70 60 50 40 30 20 10 0
ppm (t1)

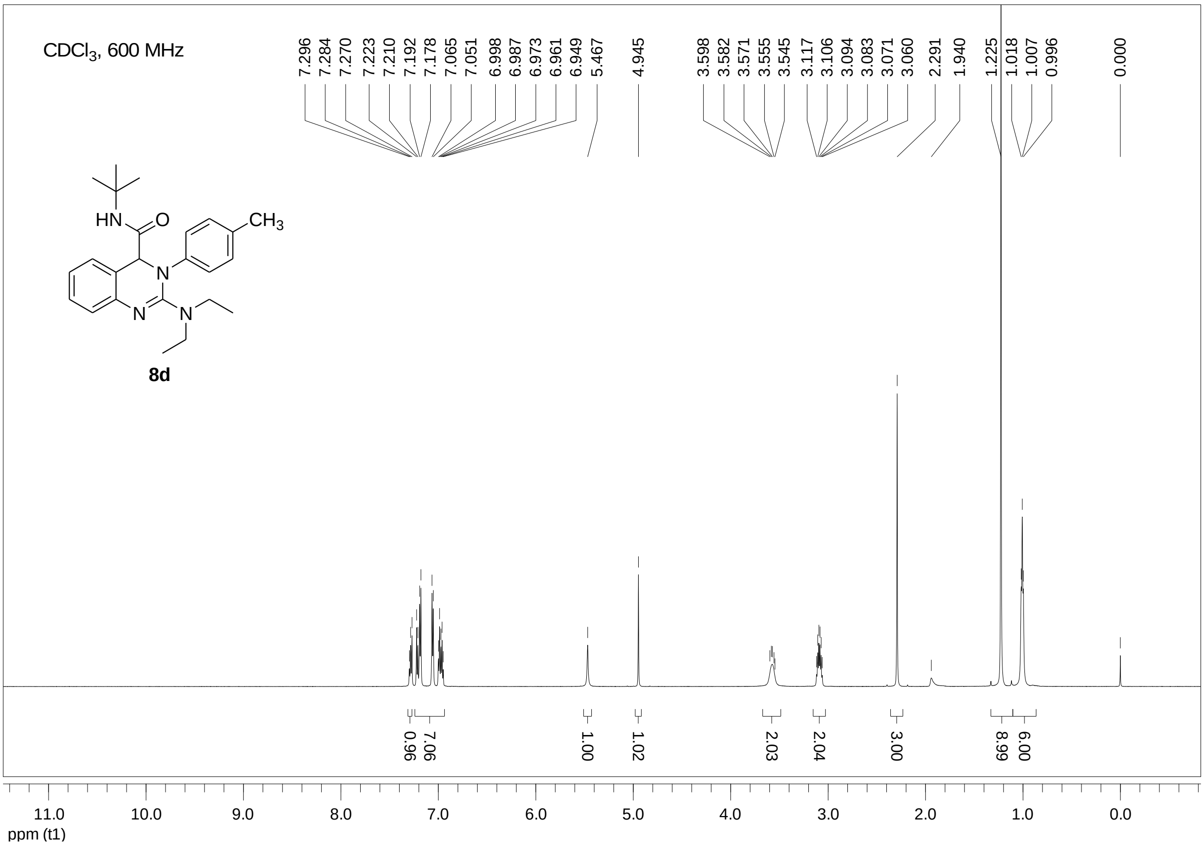
CDCl$_3$, 600 MHz
7.296 7.284 7.270 7.223 7.210 7.192 7.178 7.065 7.051 6.998 6.987 6.973 6.961 6.949 5.467 4.945 3.598 3.582 3.571 3.555 3.545 3.117 3.106 3.094 3.083 3.071 3.060 2.291 1.940 1.225 1.018 1.007 0.996 0.000
HN O CH3 N N N
8d
0.96 7.06 1.00 1.02 2.03 2.04 3.00 8.99 6.00
11.0 10.0 9.0 8.0 7.0 6.0 5.0 4.0 3.0 2.0 1.0 0.0
ppm (t1)

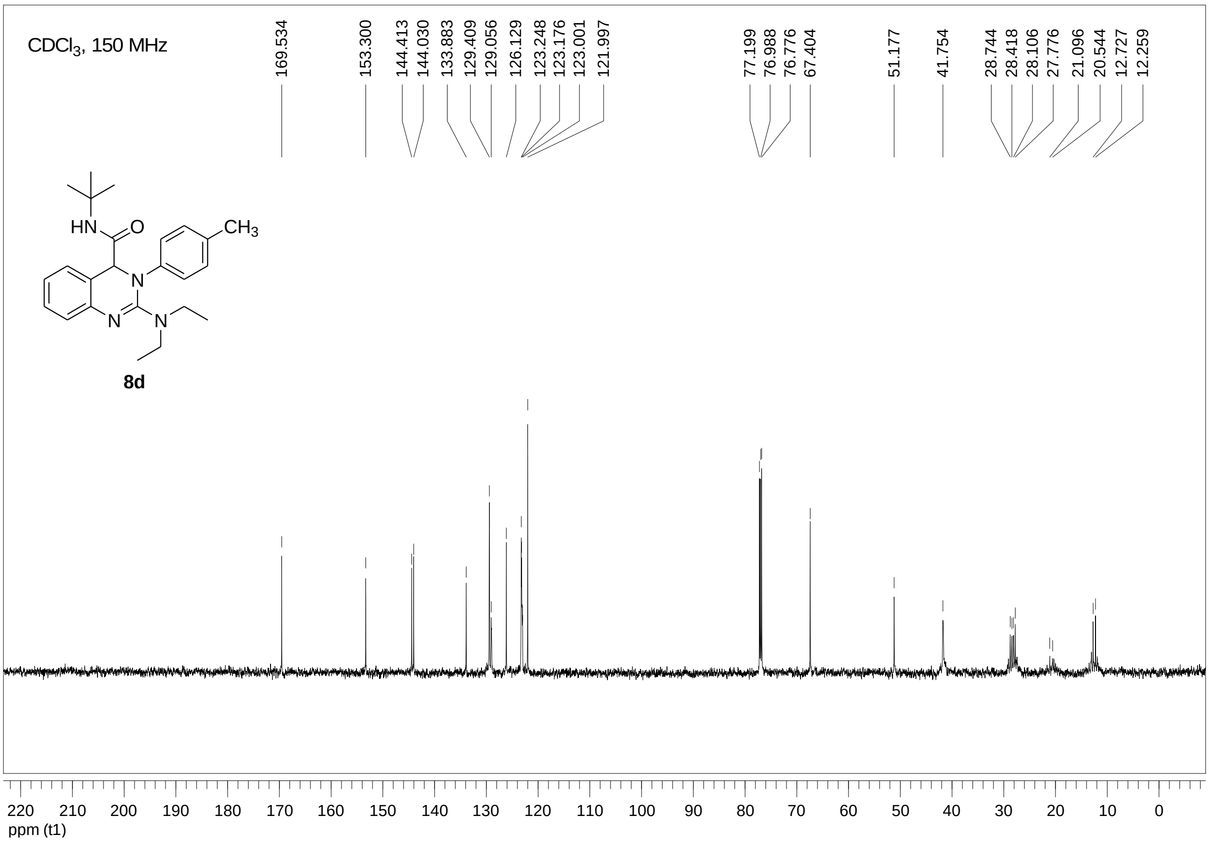
CDCl$_3$, 150 MHz
169.534 153.300 144.413 144.030 133.883 129.409 129.056 126.129 123.248 123.176 123.001 121.997 77.199 76.988 76.776 67.404 51.177 41.754 28.744 28.418 28.106 27.776 21.096 20.544 12.727 12.259
HN O CH3 N N N
8d
220 210 200 190 180 170 160 150 140 130 120 110 100 90 80 70 60 50 40 30 20 10 0
ppm (t1)

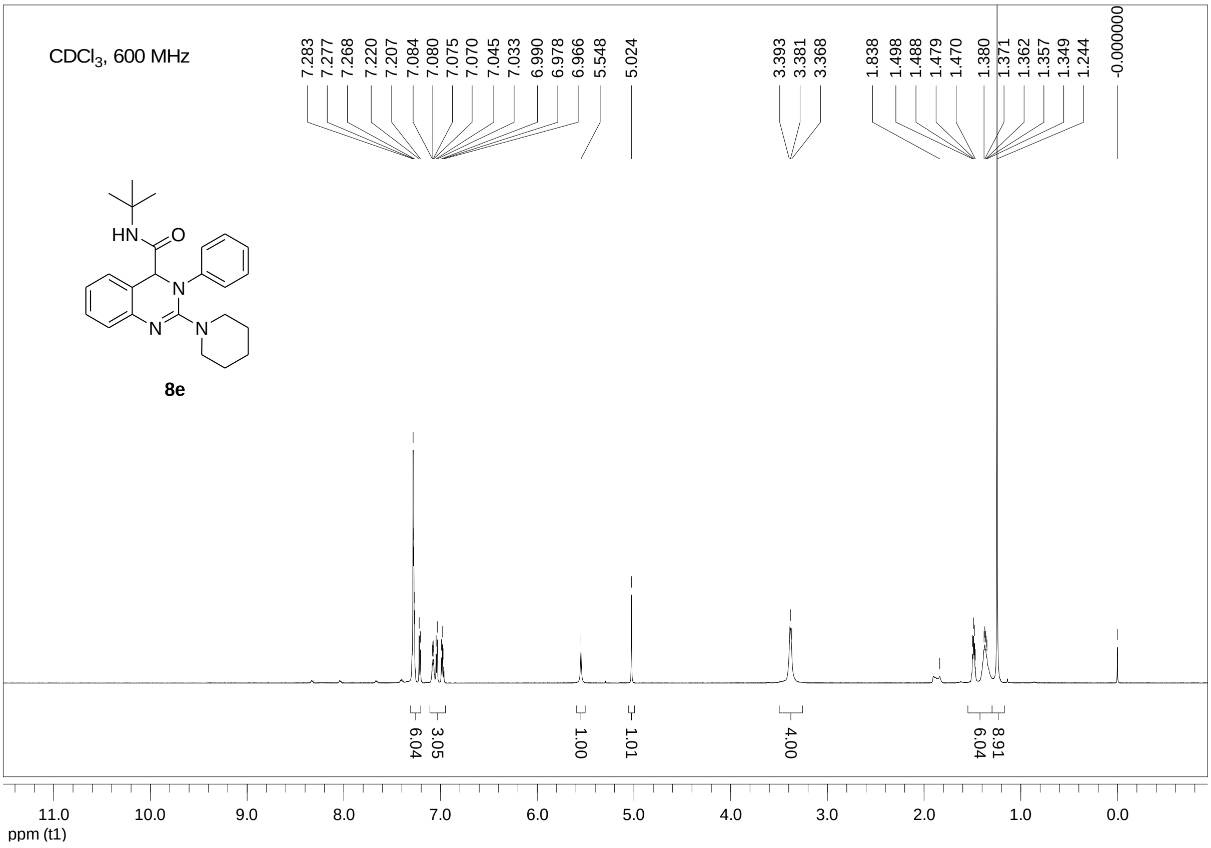
CDCl3, 600 MHz
8e

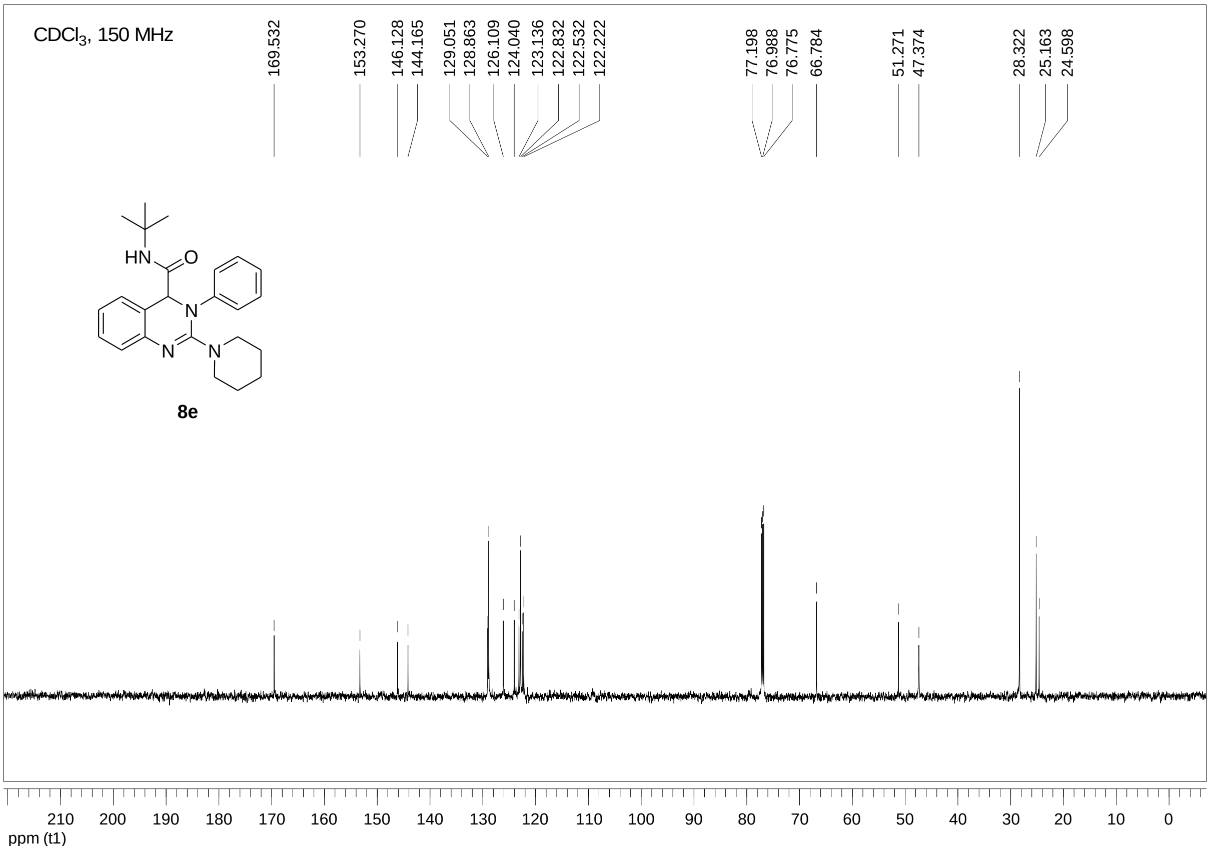
CDCl3, 150 MHz
8e

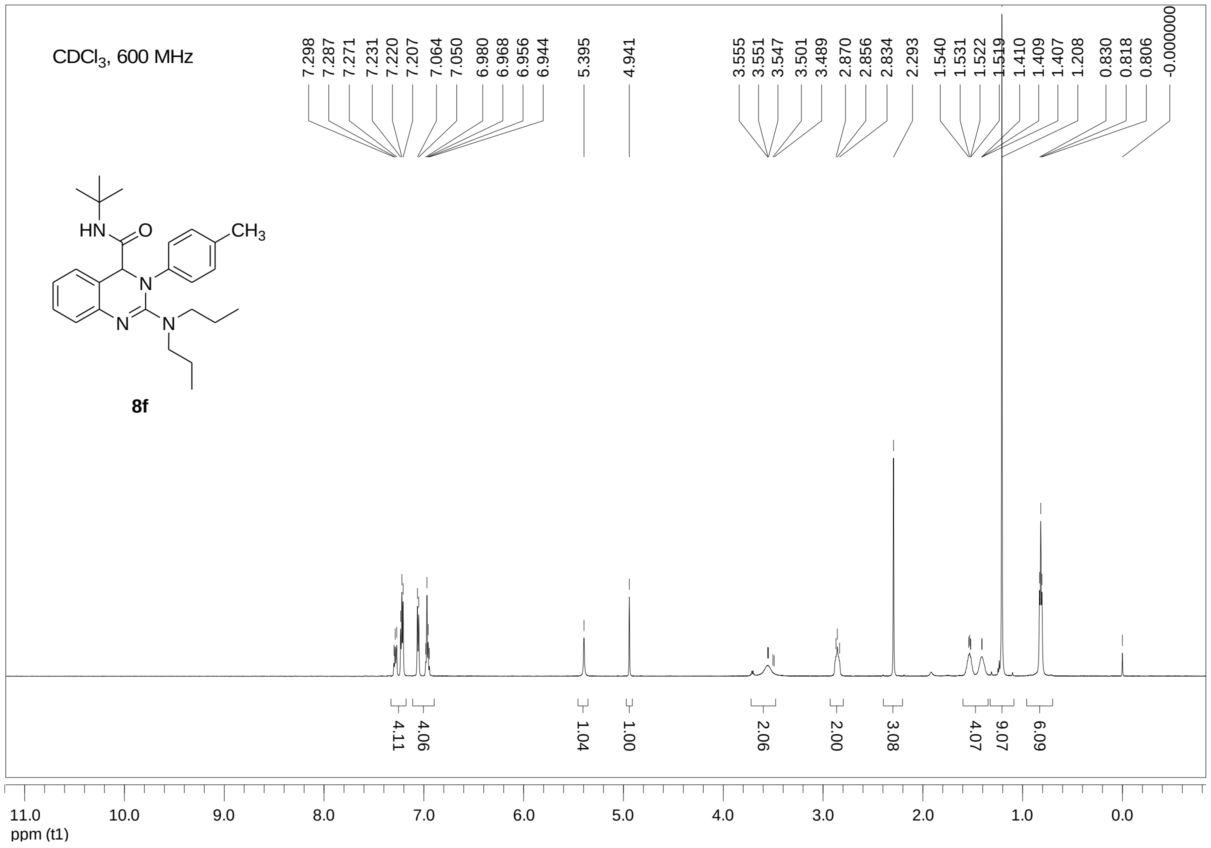
CDCl3, 600 MHz
7.298
7.287
7.271
7.231
7.220
7.207
7.064
7.050
6.980
6.968
6.956
6.944
5.395
4.941
3.555
3.551
3.547
3.501
3.489
2.870
2.856
2.834
2.293
1.540
1.531
1.522
1.519
1.410
1.409
1.407
1.208
0.830
0.818
0.806
-0.000000
HN
O
CH3
N
N
N
8f
4.11
4.06
1.04
1.00
2.06
2.00
3.08
4.07
9.07
6.09
11.0
10.0
9.0
8.0
7.0
6.0
5.0
4.0
3.0
2.0
1.0
0.0
ppm (t1)

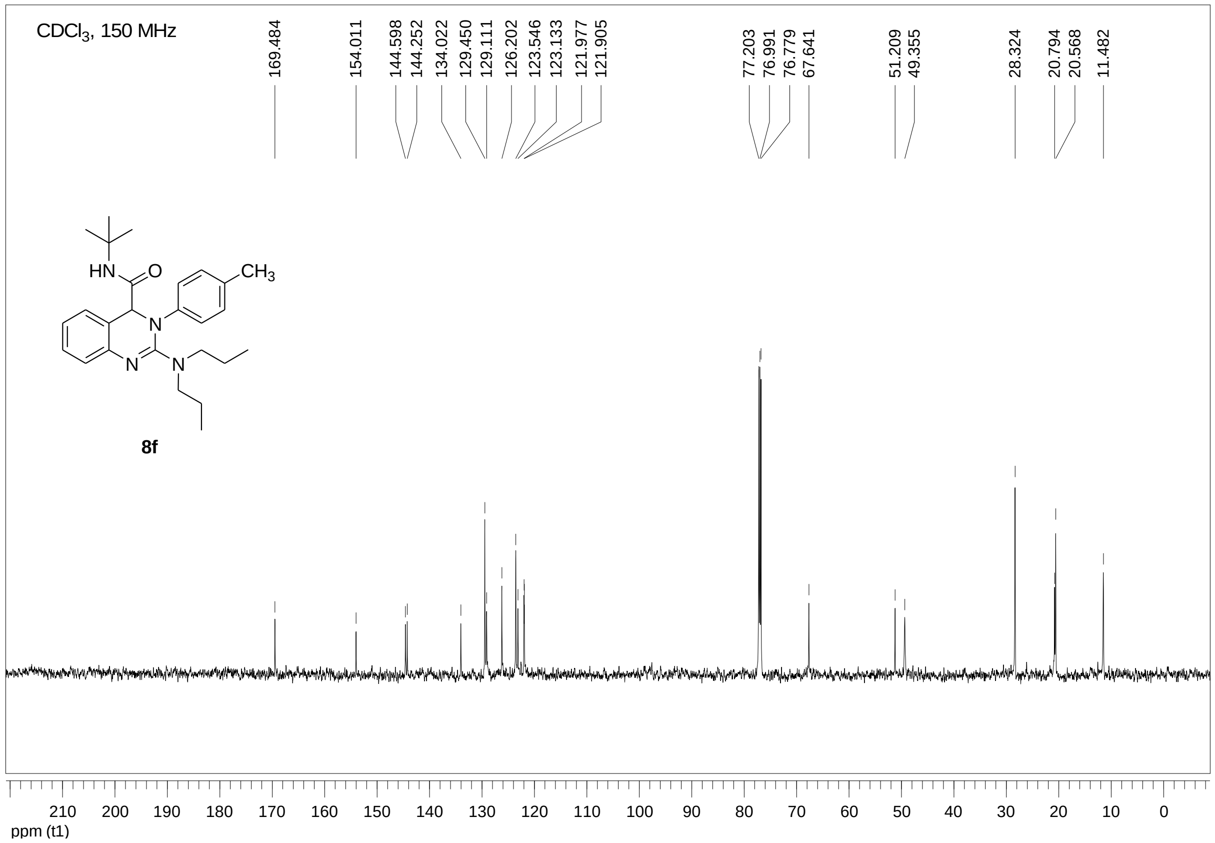
CDCl3, 150 MHz
169.484
154.011
144.598
144.252
134.022
129.450
129.111
126.202
123.546
123.133
121.977
121.905
77.203
76.991
76.779
67.641
51.209
49.355
28.324
20.794
20.568
11.482
HN
O
CH3
N
N
N
8f
210
200
190
180
170
160
150
140
130
120
110
100
90
80
70
60
50
40
30
20
10
0
ppm (t1)

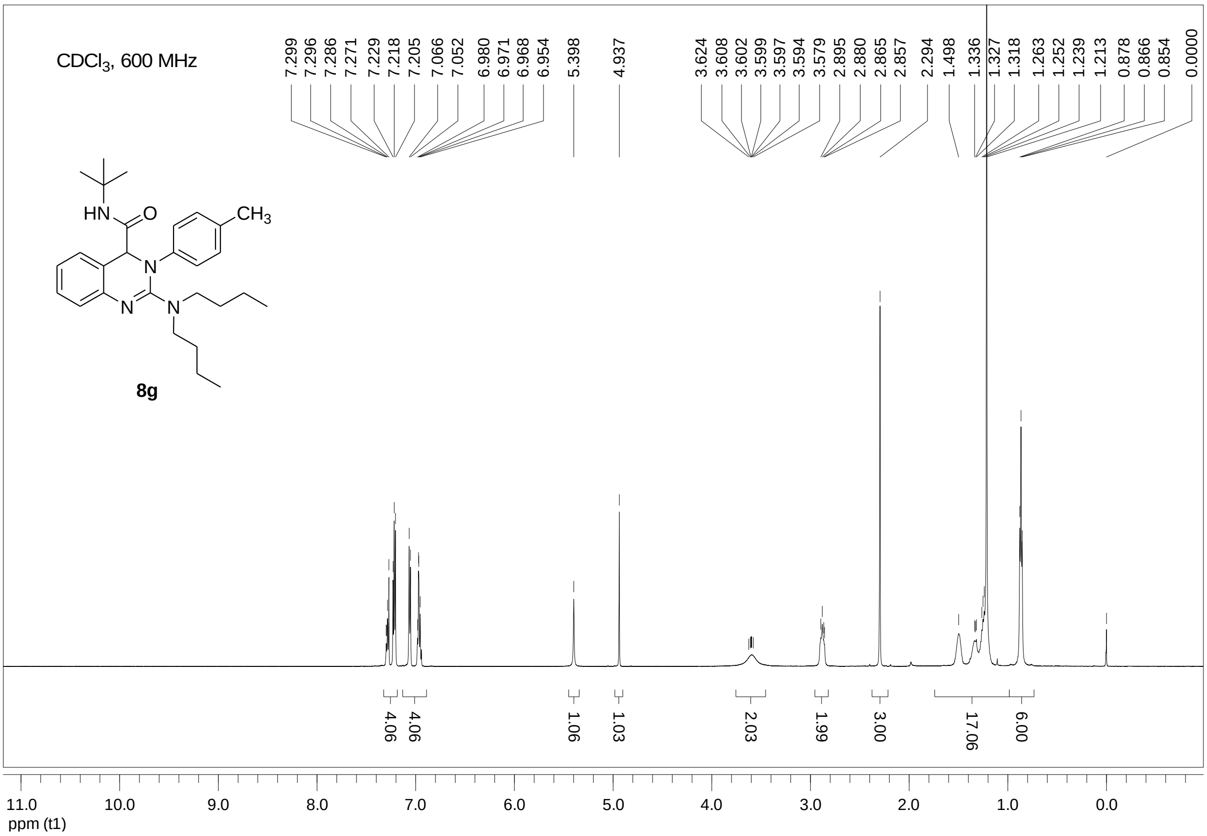
CDCl3, 600 MHz
7.299 7.296 7.286 7.271 7.229 7.218 7.205 7.066 7.052 6.980 6.971 6.968 6.954 5.398 4.937 3.624 3.608 3.602 3.599 3.597 3.594 3.579 2.895 2.880 2.865 2.857 2.294 1.498 1.336 1.327 1.318 1.263 1.252 1.239 1.213 0.878 0.866 0.854 0.0000
HN O CH3 N N N
8g
4.06 4.06 1.06 1.03 2.03 1.99 3.00 17.06 6.00
11.0 10.0 9.0 8.0 7.0 6.0 5.0 4.0 3.0 2.0 1.0 0.0
ppm (t1)

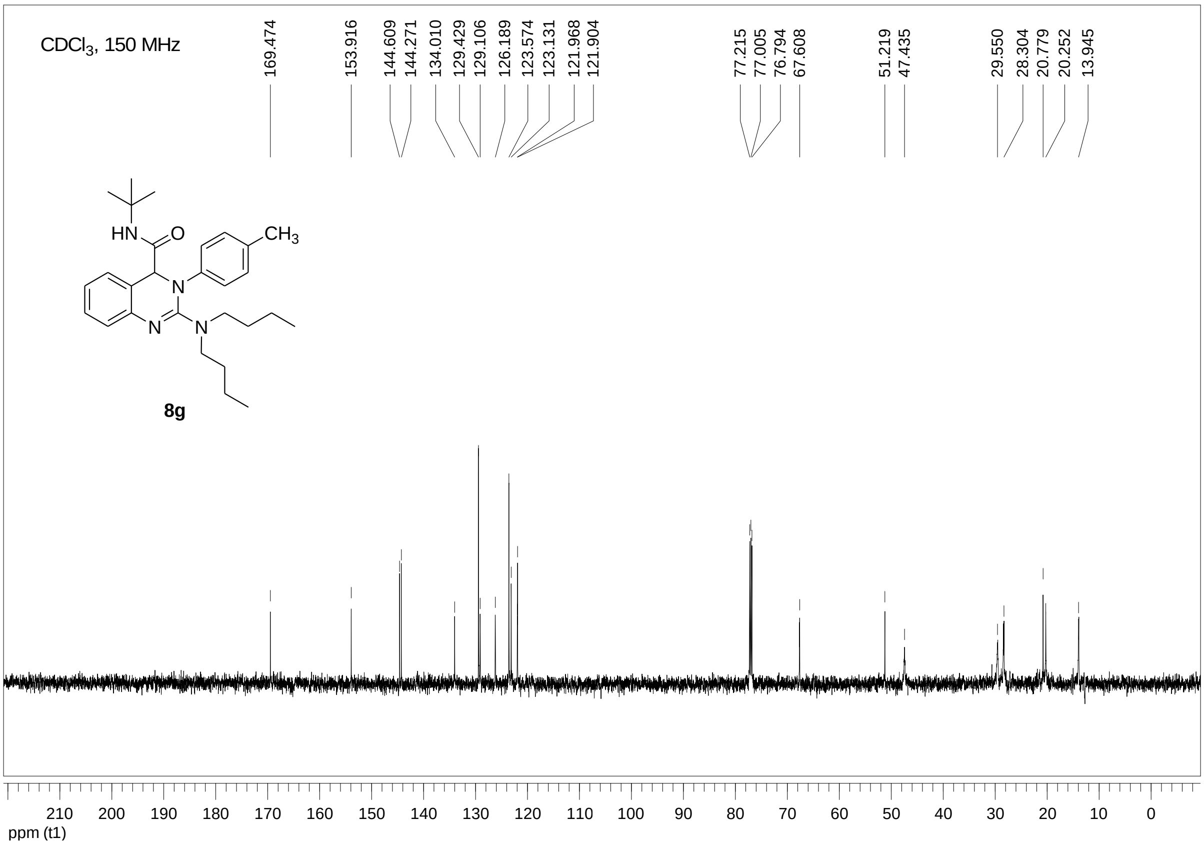
CDCl3, 150 MHz
169.474 153.916 144.609 144.271 134.010 129.429 129.106 126.189 123.574 123.131 121.968 121.904 77.215 77.005 76.794 67.608 51.219 47.435 29.550 28.304 20.779 20.252 13.945
HN O CH3 N N N
8g
210 200 190 180 170 160 150 140 130 120 110 100 90 80 70 60 50 40 30 20 10 0
ppm (t1)

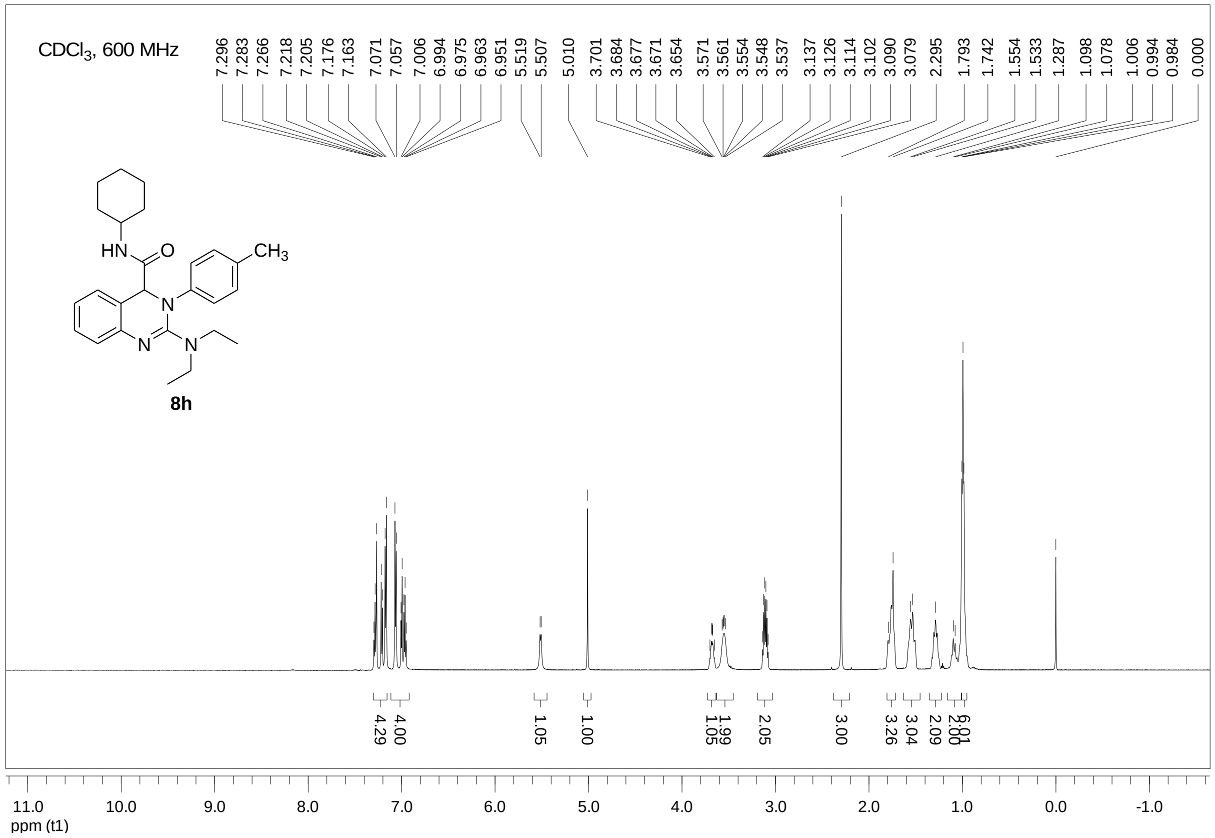

CDCl3, 600 MHz
7.296 7.283 7.266 7.218 7.205 7.176 7.163 7.071 7.057 7.006 6.994 6.975 6.963 6.951 5.519 5.507 5.010 3.701 3.684 3.677 3.671 3.654 3.571 3.561 3.554 3.548 3.537 3.137 3.126 3.114 3.102 3.090 3.079 2.295 1.793 1.742 1.554 1.533 1.287 1.098 1.078 1.006 0.994 0.984 0.000
HN
O
N
N
N
CH3
8h
4.29 4.00 1.05 1.00 1.05 1.99 2.05 3.00 3.26 3.04 2.09 2.00 6.01
11.0 10.0 9.0 8.0 7.0 6.0 5.0 4.0 3.0 2.0 1.0 0.0 -1.0
ppm (t1)

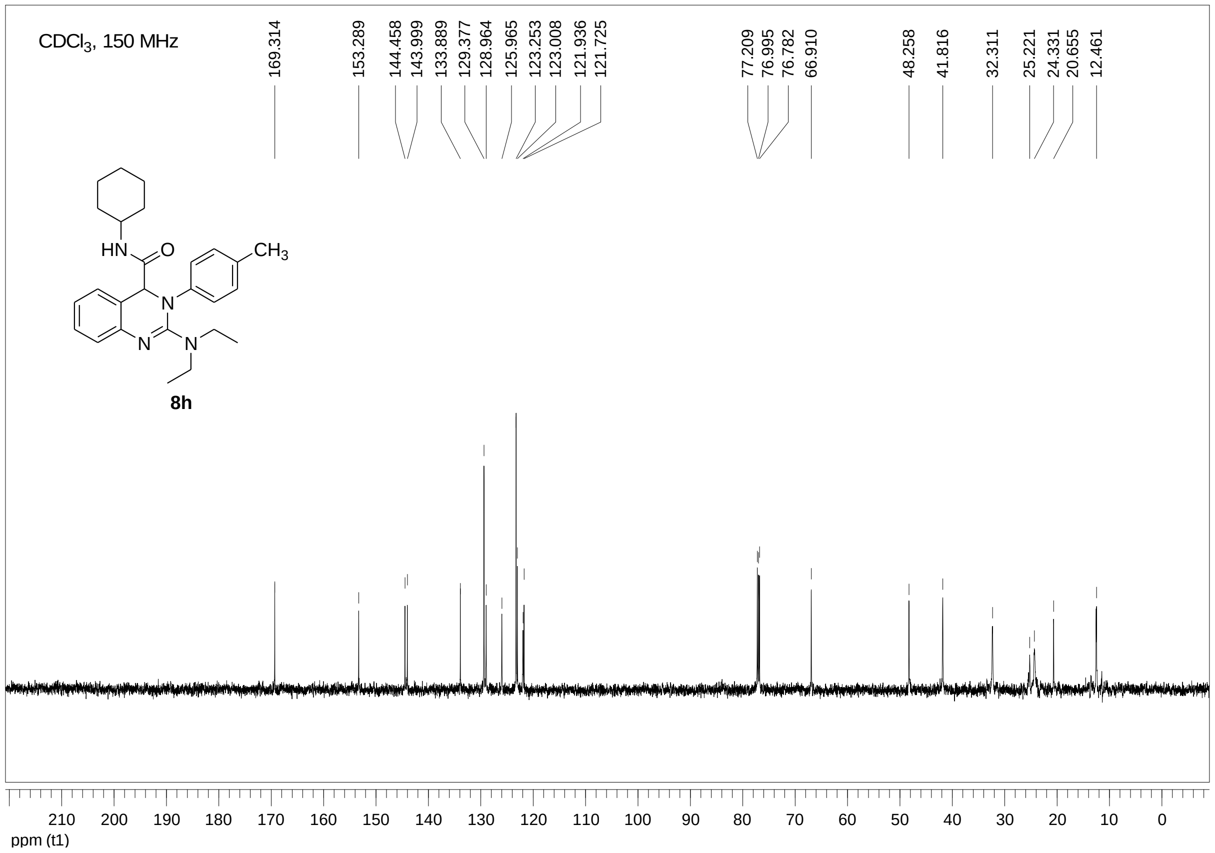

CDCl3, 150 MHz
169.314 153.289 144.458 143.999 133.889 129.377 128.964 125.965 123.253 123.008 121.936 121.725 77.209 76.995 76.782 66.910 48.258 41.816 32.311 25.221 24.331 20.655 12.461
HN
O
N
N
N
CH3
8h
210 200 190 180 170 160 150 140 130 120 110 100 90 80 70 60 50 40 30 20 10 0
ppm (t1)

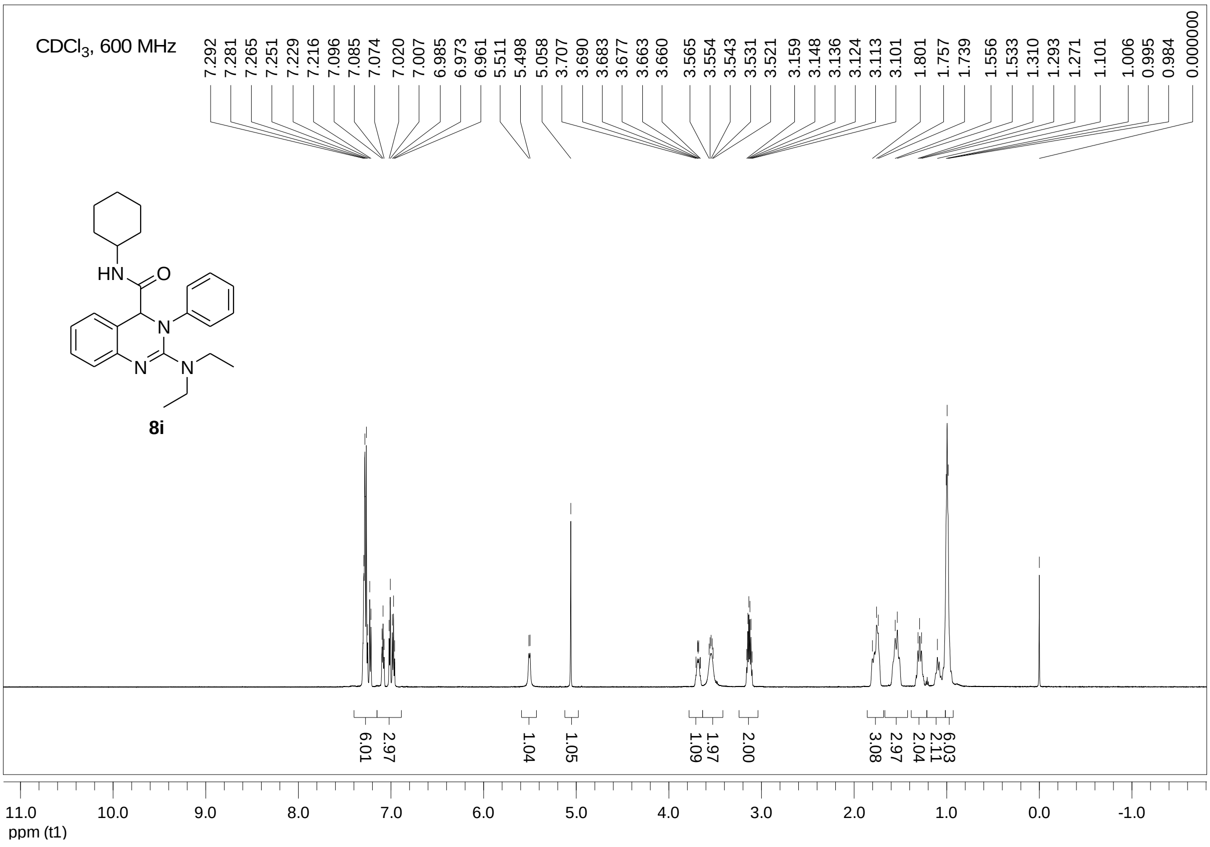
CDCl3, 600 MHz
7.292
7.281
7.265
7.251
7.229
7.216
7.096
7.085
7.074
7.020
7.007
6.985
6.973
6.961
5.511
5.498
5.058
3.707
3.690
3.683
3.677
3.663
3.660
3.565
3.554
3.543
3.531
3.521
3.159
3.148
3.136
3.124
3.113
3.101
1.801
1.757
1.739
1.556
1.533
1.310
1.293
1.271
1.101
1.006
0.995
0.984
0.000000
HN
O
N
N
N
8i
6.01
2.97
1.04
1.05
1.09
1.97
2.00
3.08
2.97
2.04
2.11
6.03
11.0
10.0
9.0
8.0
7.0
6.0
5.0
4.0
3.0
2.0
1.0
0.0
-1.0
ppm (t1)

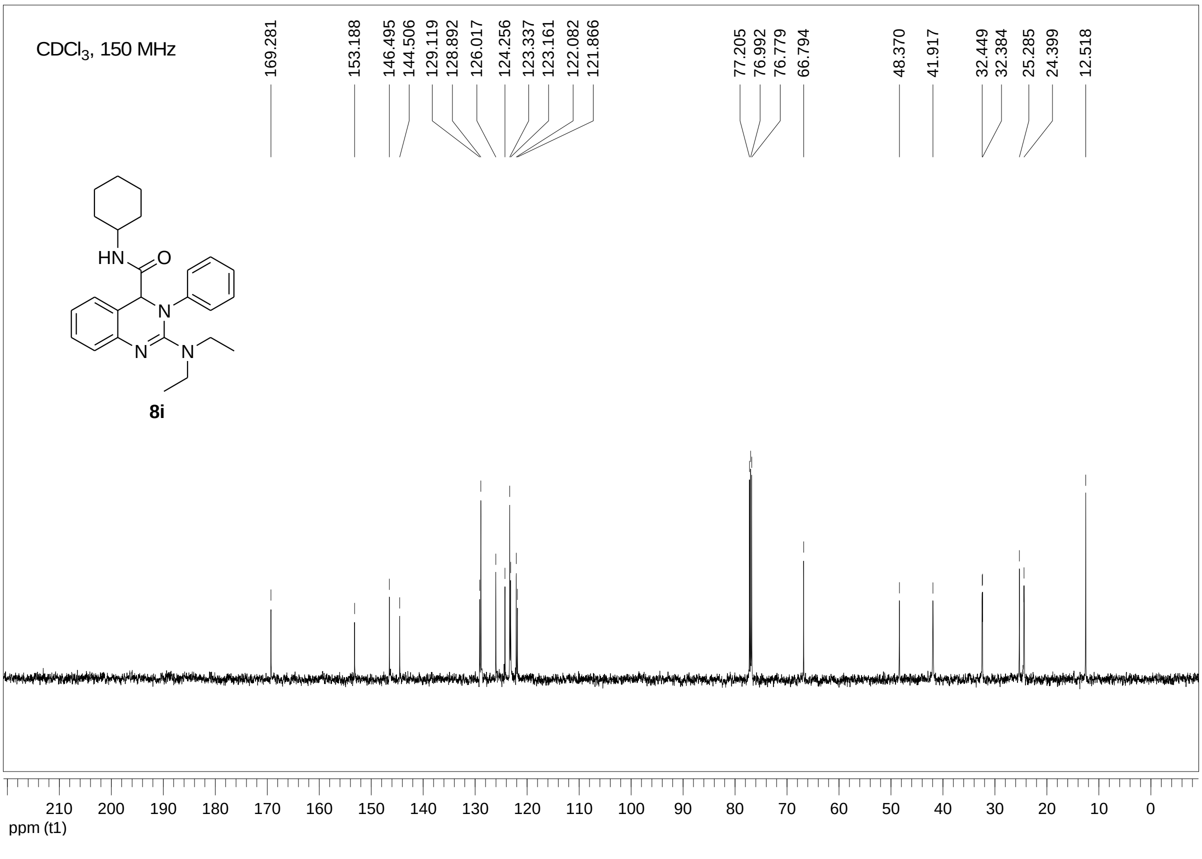
CDCl3, 150 MHz
169.281
153.188
146.495
144.506
129.119
128.892
126.017
124.256
123.337
123.161
122.082
121.866
77.205
76.992
76.779
66.794
48.370
41.917
32.449
32.384
25.285
24.399
12.518
HN
O
N
N
N
8i
210
200
190
180
170
160
150
140
130
120
110
100
90
80
70
60
50
40
30
20
10
0
ppm (t1)

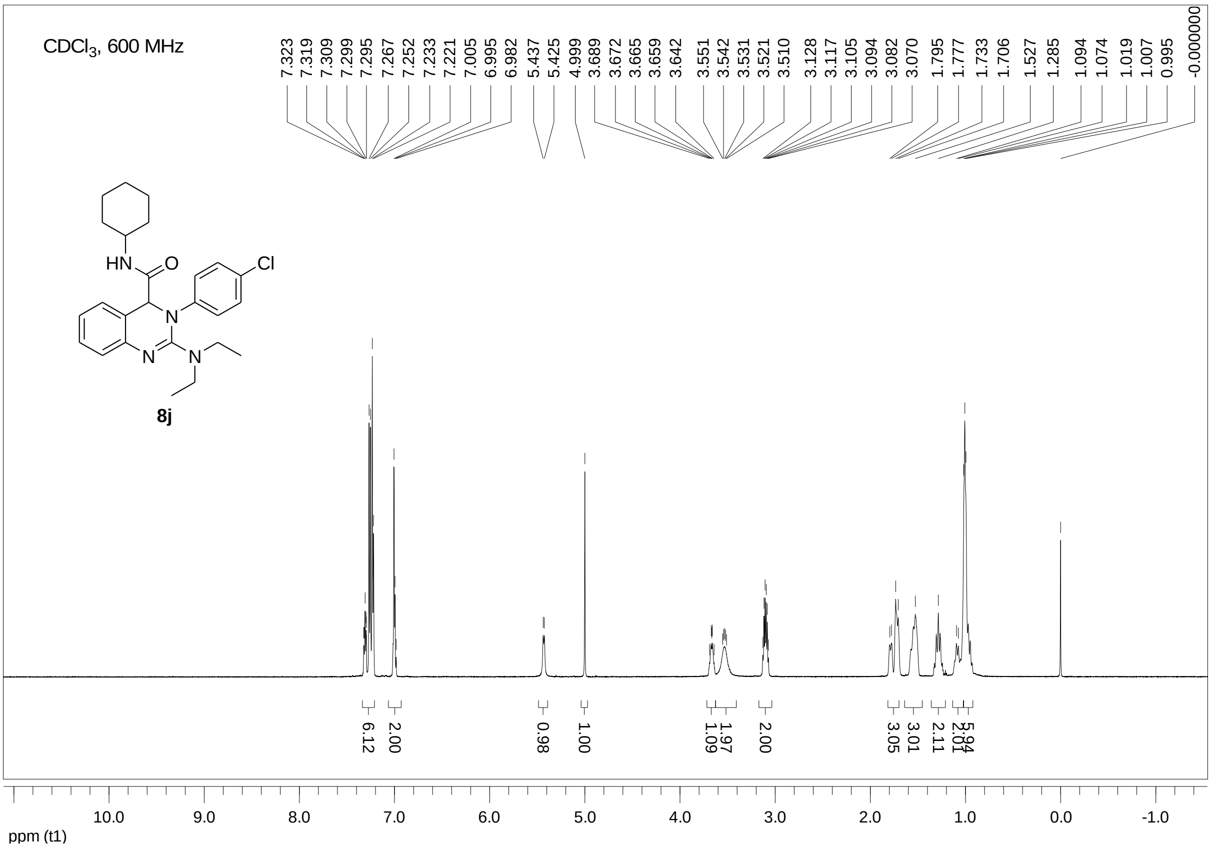
CDCl$_3$, 600 MHz
7.323 7.319 7.309 7.299 7.295 7.267 7.252 7.233 7.221 7.005 6.995 6.982 5.437 5.425 4.999 3.689 3.672 3.665 3.659 3.642 3.551 3.542 3.531 3.521 3.510 3.128 3.117 3.105 3.094 3.082 3.070 1.795 1.777 1.733 1.706 1.527 1.285 1.094 1.074 1.019 1.007 0.995 -0.000000
HN
O
N
Cl
N
N
8j
6.12 2.00 0.98 1.00 1.09 1.97 2.00 3.05 3.01 2.11 2.01 5.94
10.0 9.0 8.0 7.0 6.0 5.0 4.0 3.0 2.0 1.0 0.0 -1.0
ppm (t1)

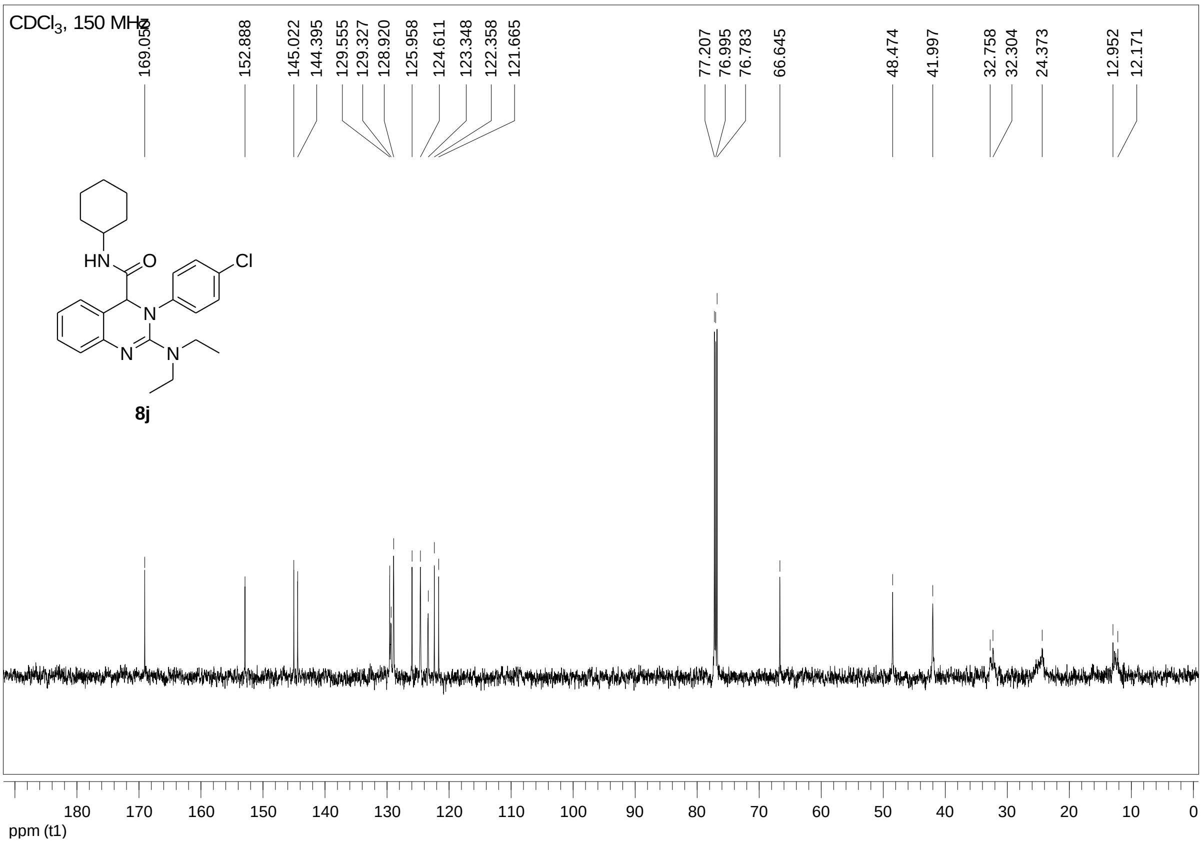
CDCl$_3$, 150 MHz
169.052 152.888 145.022 144.395 129.555 129.327 128.920 125.958 124.611 123.348 122.358 121.665 77.207 76.995 76.783 66.645 48.474 41.997 32.758 32.304 24.373 12.952 12.171
HN
O
N
Cl
N
N
8j
180 170 160 150 140 130 120 110 100 90 80 70 60 50 40 30 20 10 0
ppm (t1)

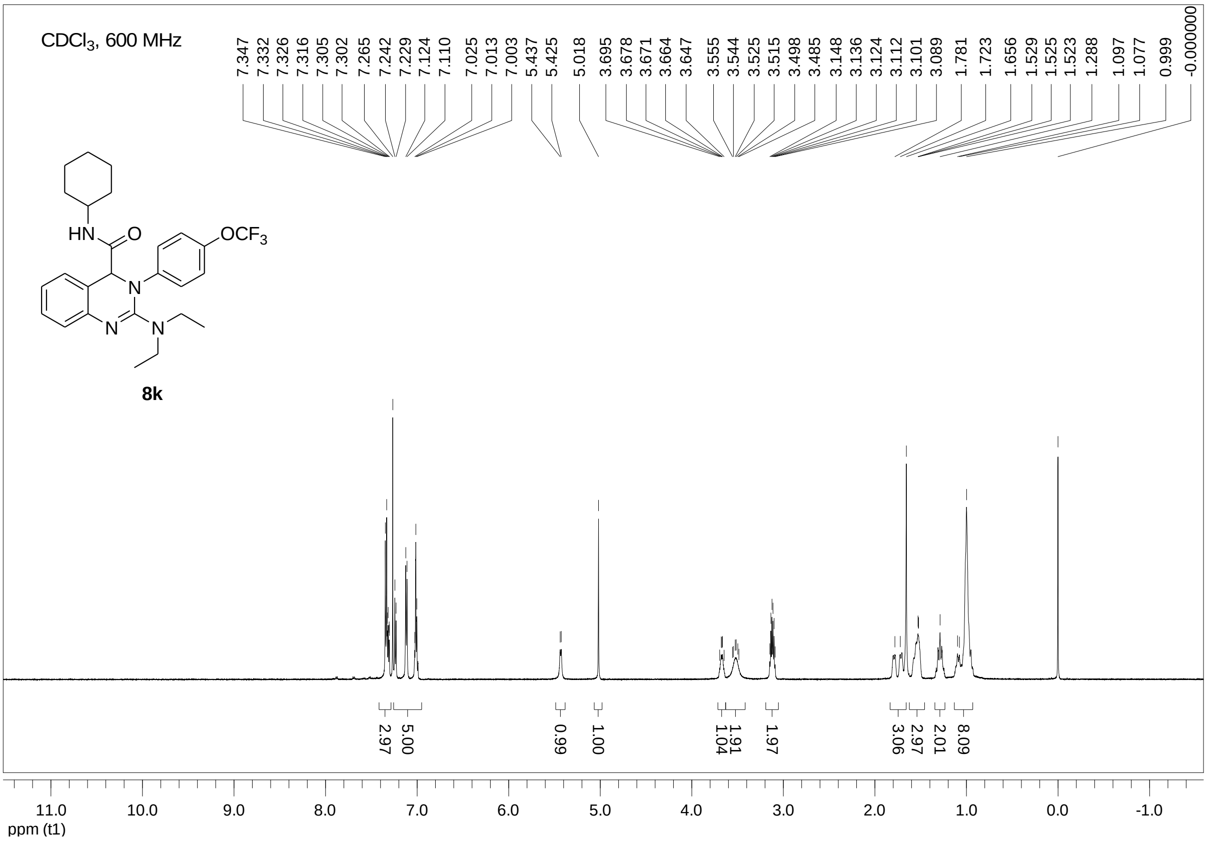
CDCl3, 600 MHz
7.347 7.332 7.326 7.316 7.305 7.302 7.265 7.242 7.229 7.124 7.110 7.025 7.013 7.003 5.437 5.425 5.018 3.695 3.678 3.671 3.664 3.647 3.555 3.544 3.525 3.515 3.498 3.485 3.148 3.136 3.124 3.112 3.101 3.089 1.781 1.723 1.656 1.529 1.525 1.523 1.288 1.097 1.077 0.999 -0.000000
HN
O
OCF3
N
N
N
8k
2.97 5.00 0.99 1.00 1.04 1.91 1.97 3.06 2.97 2.01 8.09
11.0 10.0 9.0 8.0 7.0 6.0 5.0 4.0 3.0 2.0 1.0 0.0 -1.0
ppm (t1)

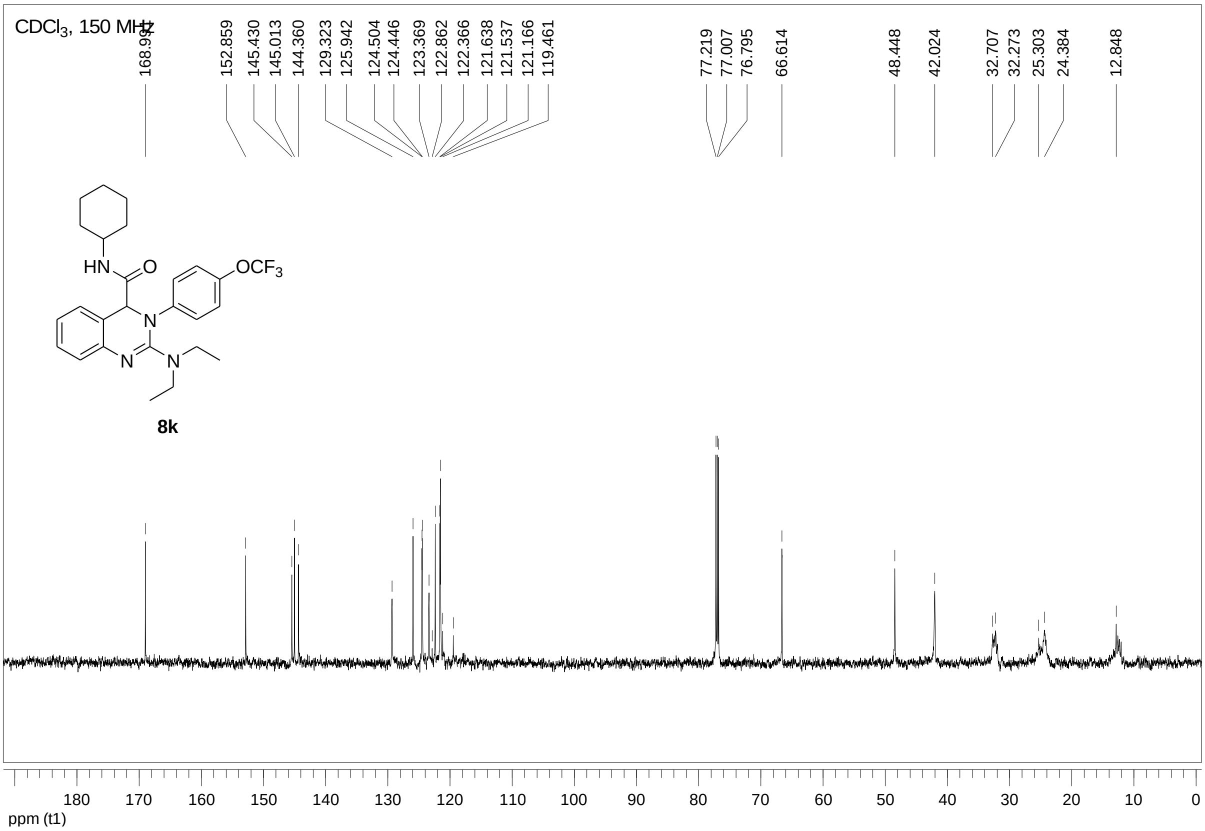
CDCl3, 150 MHz
152.859 145.430 145.013 144.360 129.323 125.942 124.504 124.446 123.369 122.862 122.366 121.638 121.537 121.166 119.461 77.219 77.007 76.795 66.614 48.448 42.024 32.707 32.273 25.303 24.384 12.848
HN
O
OCF3
N
N
N
8k
180 170 160 150 140 130 120 110 100 90 80 70 60 50 40 30 20 10 0
ppm (t1)

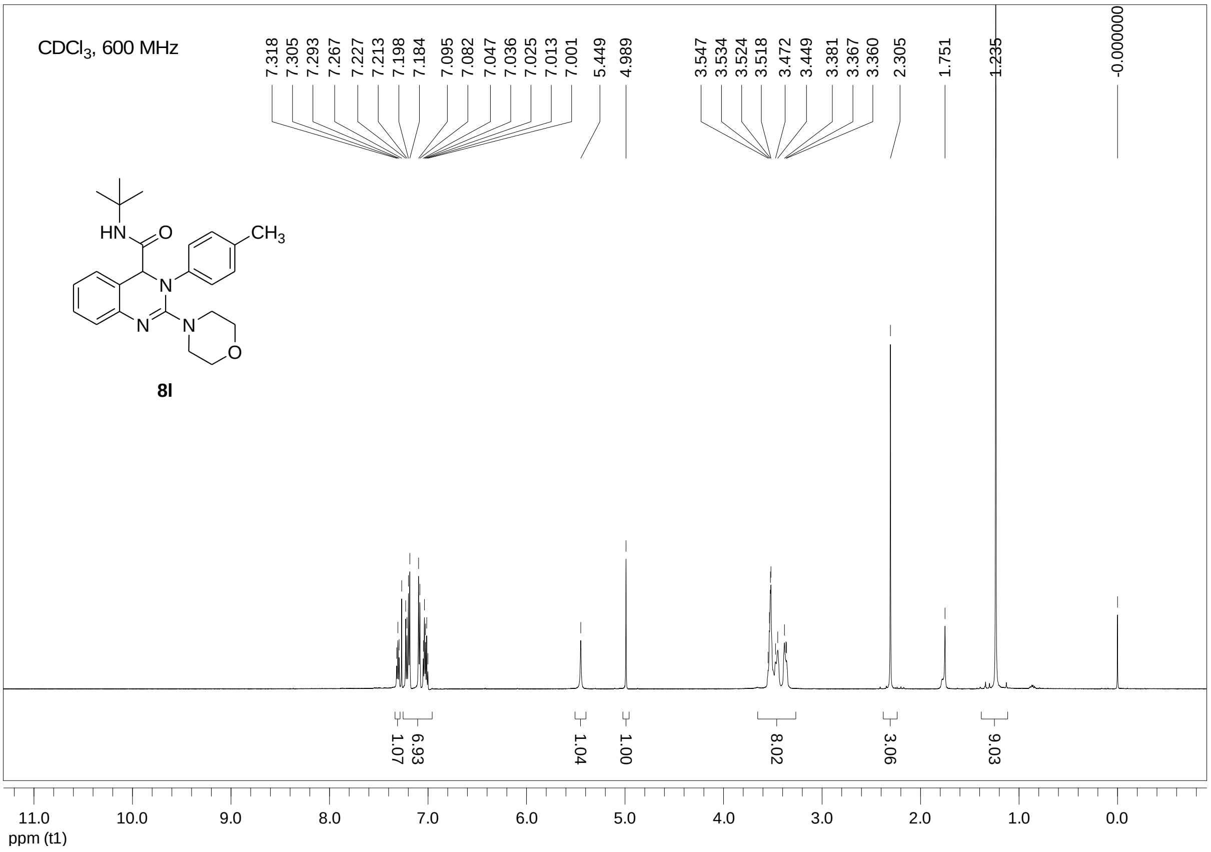
CDCl3, 600 MHz
7.318 7.305 7.293 7.267 7.227 7.213 7.198 7.184 7.095 7.082 7.047 7.036 7.025 7.013 7.001 5.449 4.989
3.547 3.534 3.524 3.518 3.472 3.449 3.381 3.367 3.360 2.305 1.751 1.235 -0.000000
HN
O
CH3
N
N
N
O
8l
1.07 6.93 1.04 1.00 8.02 3.06 9.03
11.0 10.0 9.0 8.0 7.0 6.0 5.0 4.0 3.0 2.0 1.0 0.0
ppm (t1)

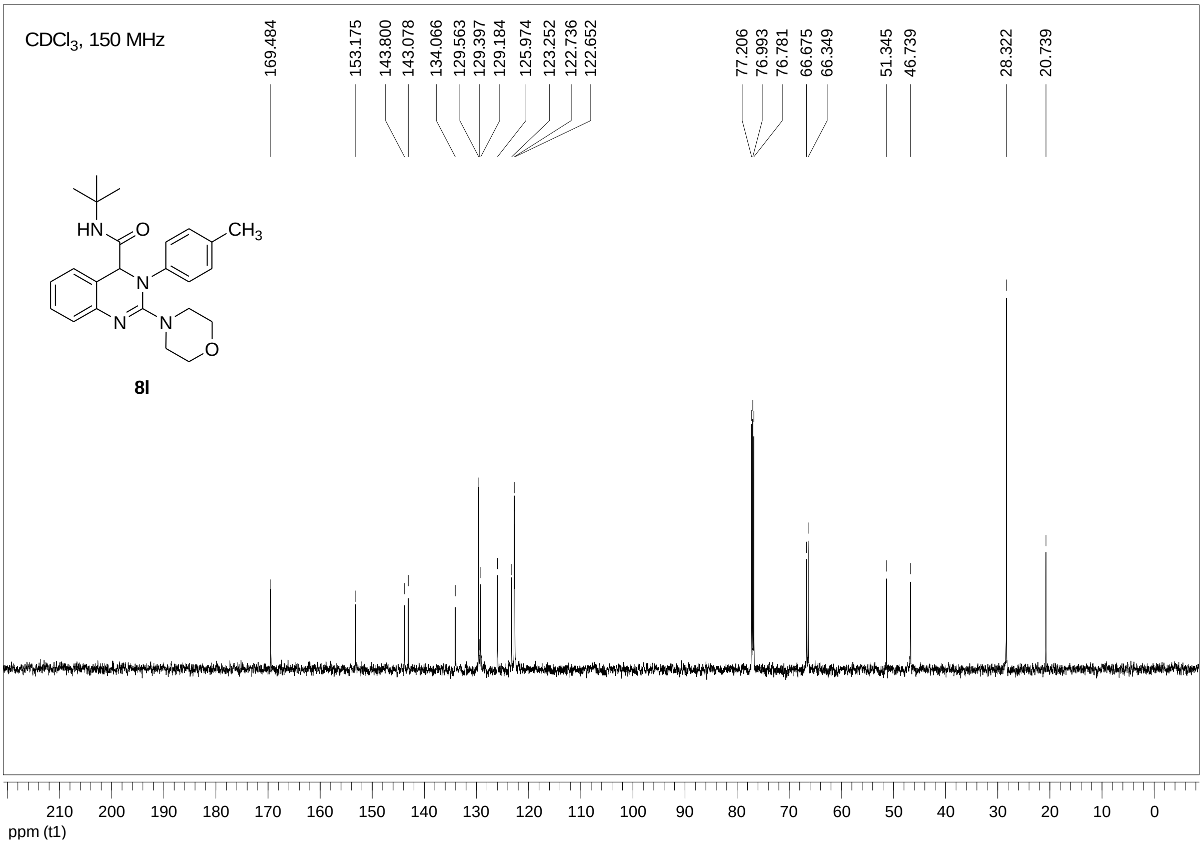
CDCl3, 150 MHz
169.484
153.175 143.800 143.078 134.066 129.563 129.397 129.184 125.974 123.252 122.736 122.652
77.206 76.993 76.781 66.675 66.349 51.345 46.739 28.322 20.739
HN
O
CH3
N
N
N
O
8l
210 200 190 180 170 160 150 140 130 120 110 100 90 80 70 60 50 40 30 20 10 0
ppm (t1)

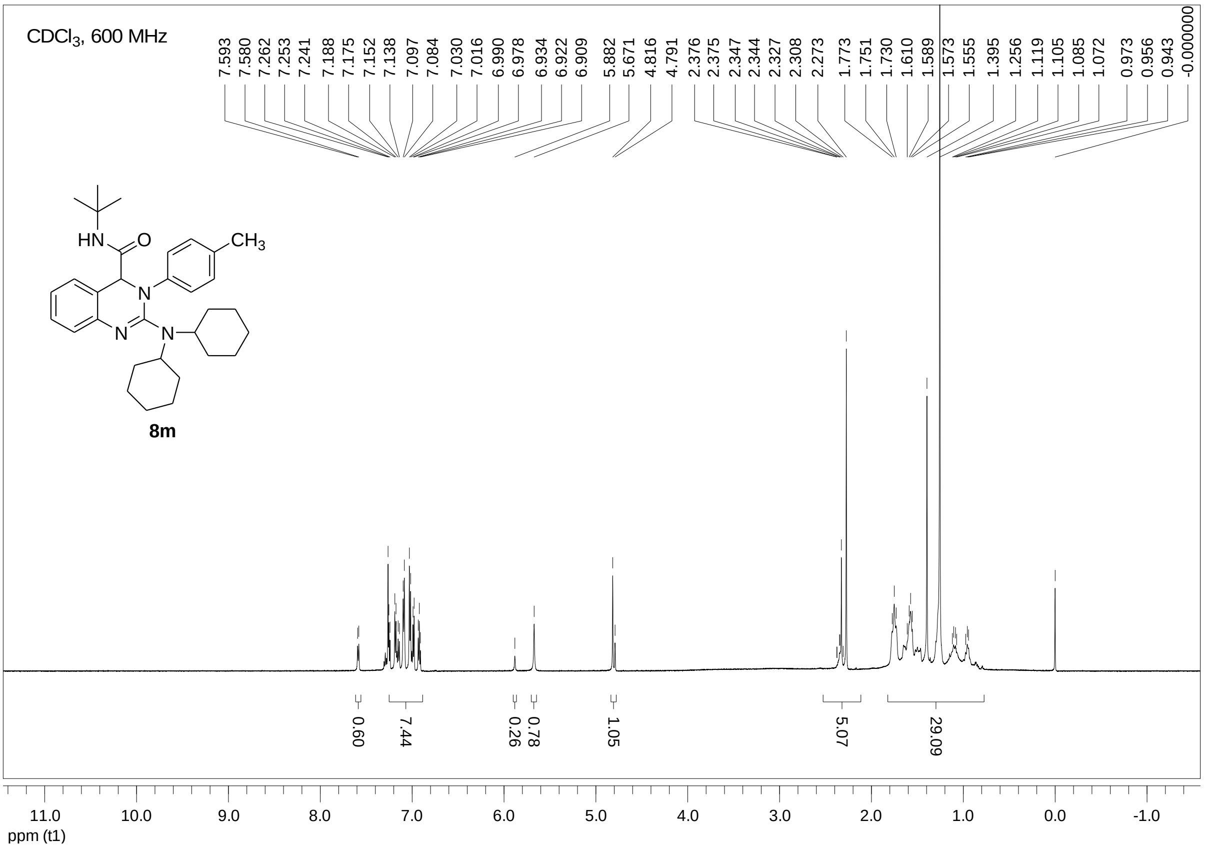
CDCl3, 600 MHz
7.593 7.580 7.262 7.253 7.241 7.188 7.175 7.152 7.138 7.097 7.084 7.030 7.016 6.990 6.978 6.934 6.922 6.909 5.882 5.671 4.816 4.791 2.376 2.375 2.347 2.344 2.327 2.308 2.273 1.773 1.751 1.730 1.610 1.589 1.573 1.555 1.395 1.256 1.119 1.105 1.085 1.072 0.973 0.956 0.943 -0.000000
HN
O
CH3
N
N
N
8m
0.60
7.44
0.26
0.78
1.05
5.07
29.09
11.0 10.0 9.0 8.0 7.0 6.0 5.0 4.0 3.0 2.0 1.0 0.0 -1.0
ppm (t1)

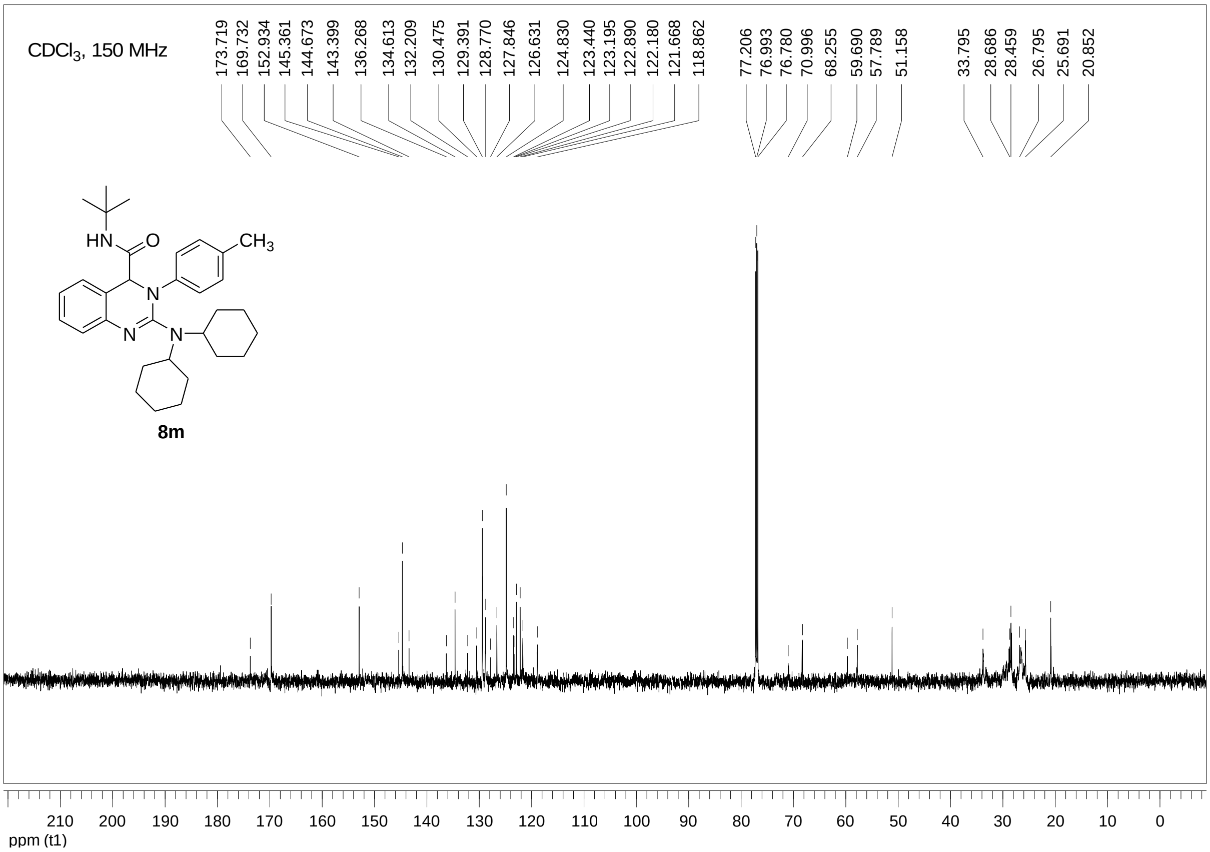
CDCl3, 150 MHz
173.719 169.732 152.934 145.361 144.673 143.399 136.268 134.613 132.209 130.475 129.391 128.770 127.846 126.631 124.830 123.440 123.195 122.890 122.180 121.668 118.862
77.206 76.993 76.780 70.996 68.255 59.690 57.789 51.158
33.795 28.686 28.459 26.795 25.691 20.852
HN
O
CH3
N
N
N
8m
210 200 190 180 170 160 150 140 130 120 110 100 90 80 70 60 50 40 30 20 10 0
ppm (t1)

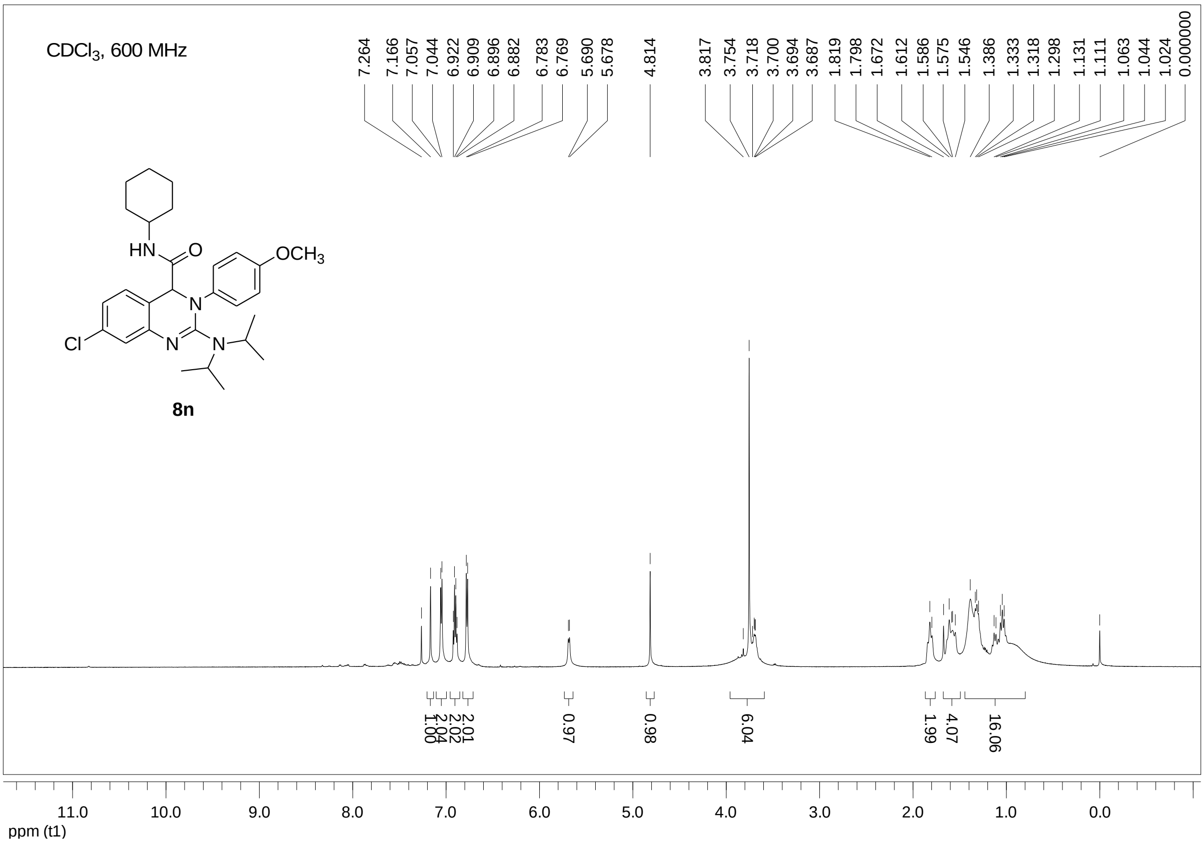
CDCl3, 600 MHz
7.264
7.166
7.057
7.044
6.922
6.909
6.896
6.882
6.783
6.769
5.690
5.678
4.814
3.817
3.754
3.718
3.700
3.694
3.687
1.819
1.798
1.672
1.612
1.586
1.575
1.546
1.386
1.333
1.318
1.298
1.131
1.111
1.063
1.044
1.024
0.000000
HN
O
OCH3
N
Cl
N
N
8n
1.00
1.04
2.02
2.01
0.97
0.98
6.04
1.99
4.07
16.06
11.0
10.0
9.0
8.0
7.0
6.0
5.0
4.0
3.0
2.0
1.0
0.0
ppm (t1)

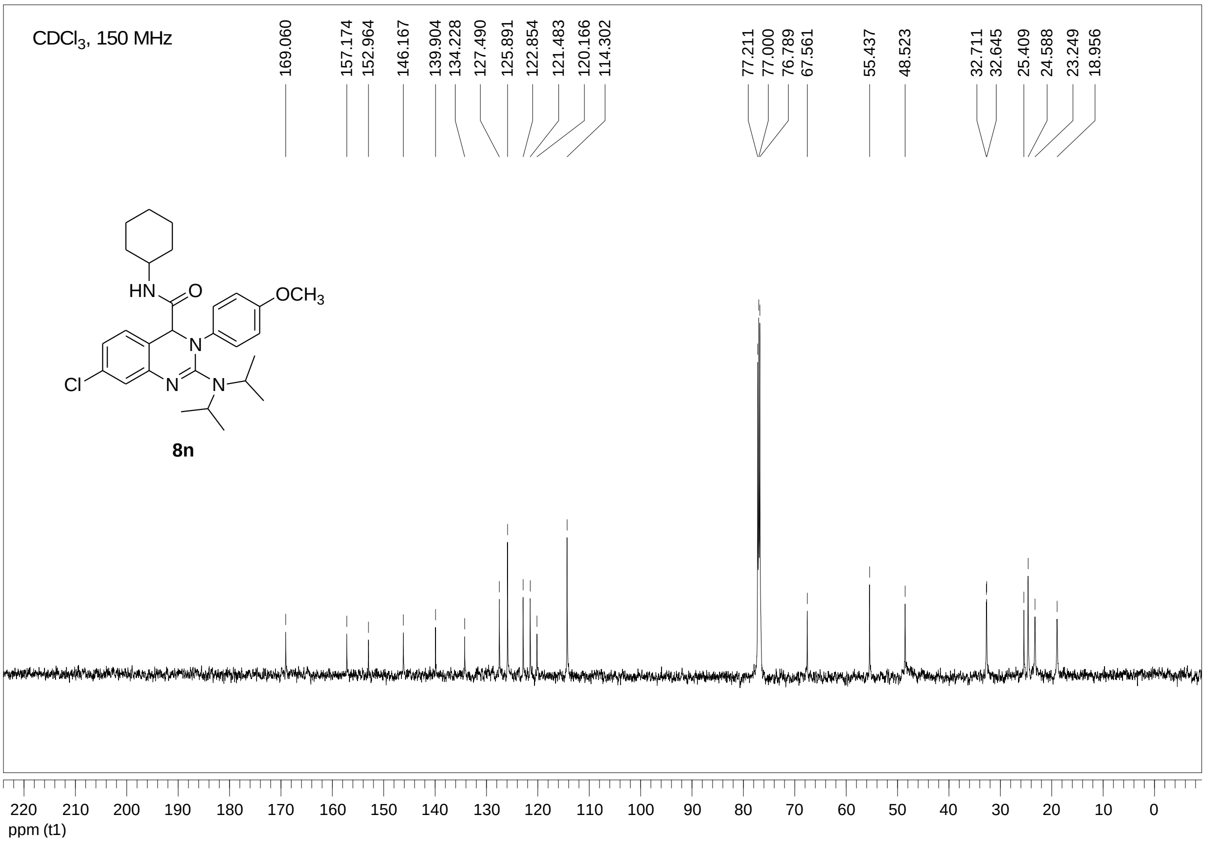
CDCl3, 150 MHz
169.060
157.174
152.964
146.167
139.904
134.228
127.490
125.891
122.854
121.483
120.166
114.302
77.211
77.000
76.789
67.561
55.437
48.523
32.711
32.645
25.409
24.588
23.249
18.956
HN
O
OCH3
N
Cl
N
N
8n
220
210
200
190
180
170
160
150
140
130
120
110
100
90
80
70
60
50
40
30
20
10
0
ppm (t1)

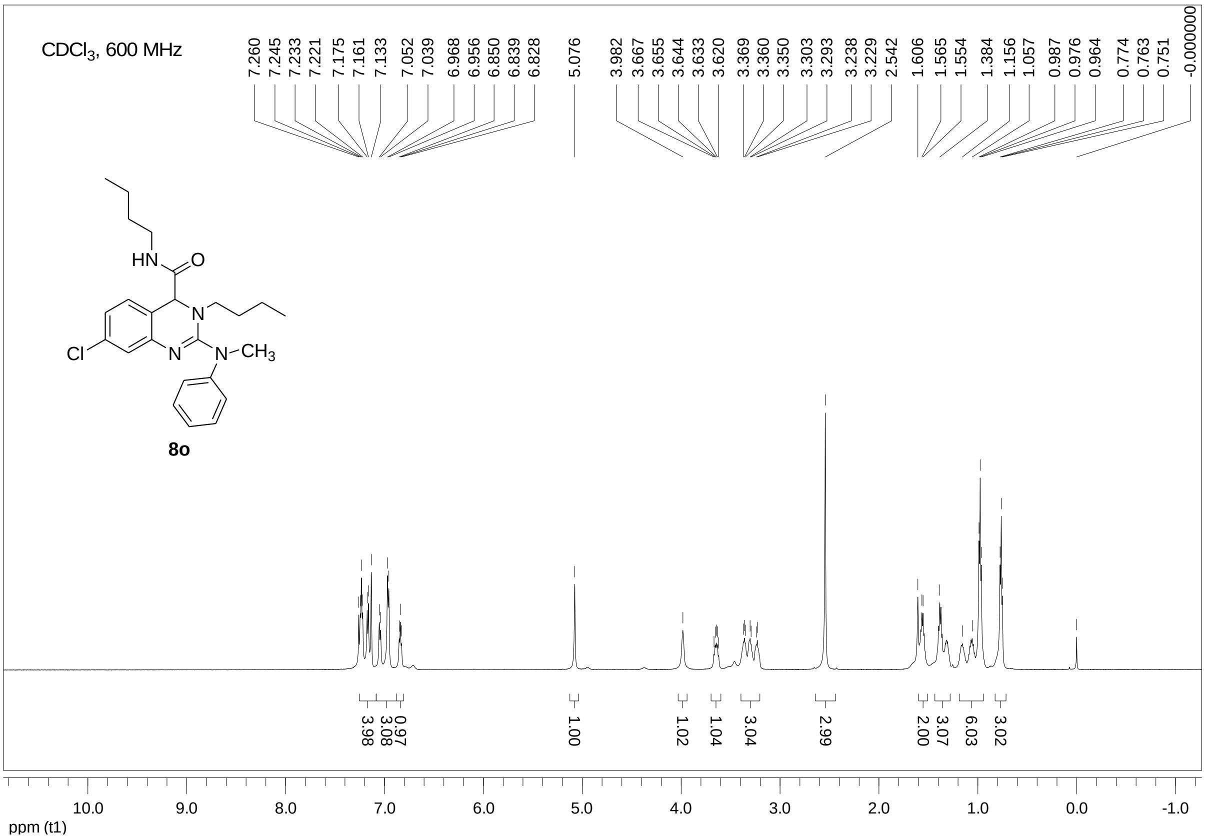
CDCl$_3$, 600 MHz
7.260
7.245
7.233
7.221
7.175
7.161
7.133
7.052
7.039
6.968
6.956
6.850
6.839
6.828
5.076
3.982
3.667
3.655
3.644
3.633
3.620
3.369
3.360
3.350
3.303
3.293
3.238
3.229
2.542
1.606
1.565
1.554
1.384
1.156
1.057
0.987
0.976
0.964
0.774
0.763
0.751
-0.000000
HN
O
N
Cl
N
N
CH$_3$
8o
3.98
3.08
0.97
1.00
1.02
1.04
3.04
2.99
2.00
3.07
6.03
3.02
10.0
9.0
8.0
7.0
6.0
5.0
4.0
3.0
2.0
1.0
0.0
-1.0
ppm (t1)

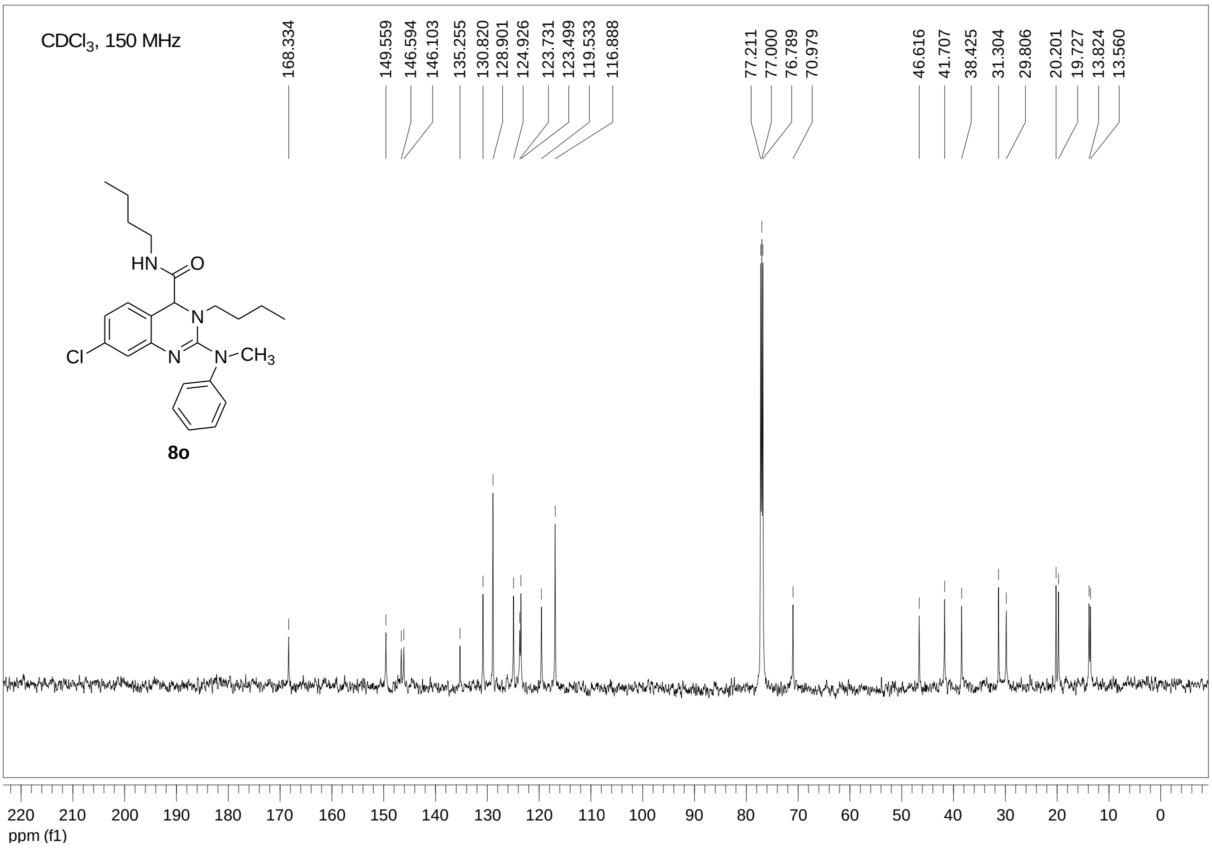
CDCl$_3$, 150 MHz
168.334
149.559
146.594
146.103
135.255
130.820
128.901
124.926
123.731
123.499
119.533
116.888
77.211
77.000
76.789
70.979
46.616
41.707
38.425
31.304
29.806
20.201
19.727
13.824
13.560
HN
O
N
Cl
N
N
CH$_3$
8o
220
210
200
190
180
170
160
150
140
130
120
110
100
90
80
70
60
50
40
30
20
10
0
ppm (f1)

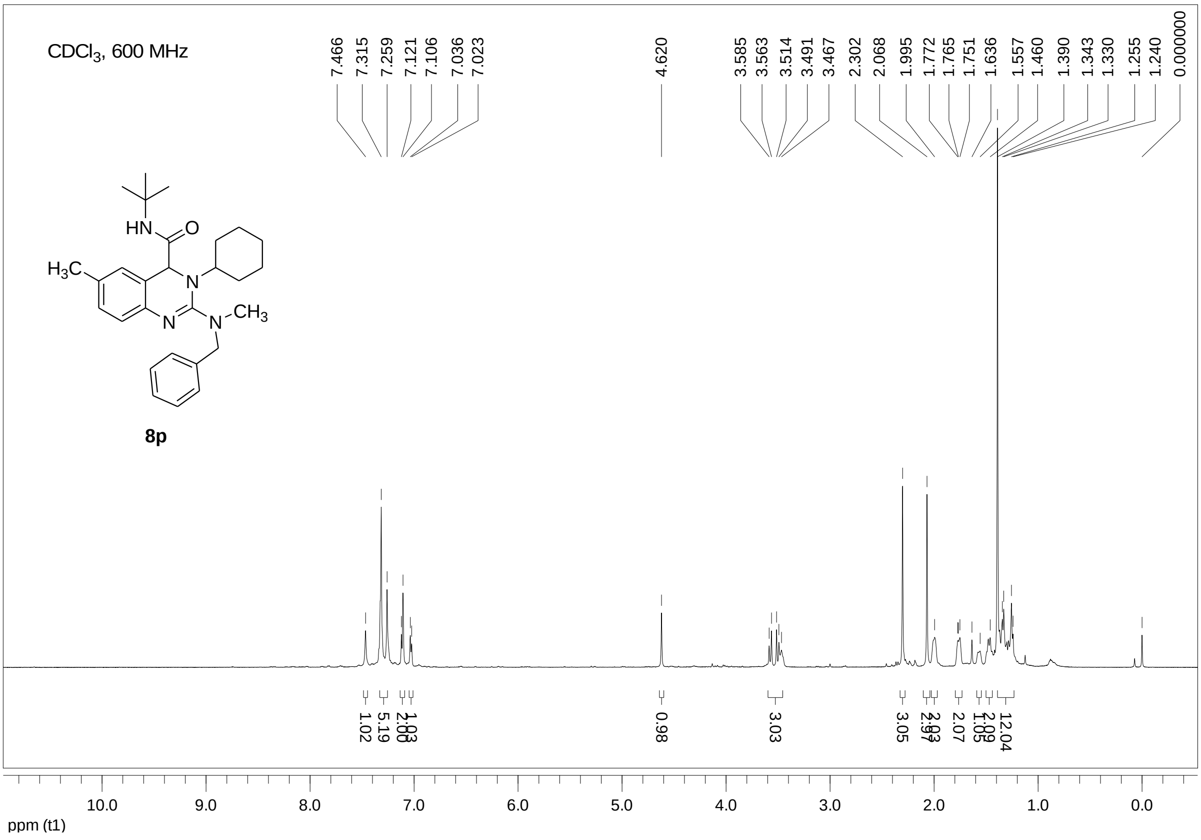
CDCl3, 600 MHz
7.466
7.315
7.259
7.121
7.106
7.036
7.023
4.620
3.585
3.563
3.514
3.491
3.467
2.302
2.068
1.995
1.772
1.765
1.751
1.636
1.557
1.460
1.390
1.343
1.330
1.255
1.240
0.000000
HN
O
H3C
N
N
N
CH3
8p
1.02
5.19
2.00
1.03
0.98
3.03
3.05
2.97
2.93
2.07
1.05
2.09
12.04
10.0
9.0
8.0
7.0
6.0
5.0
4.0
3.0
2.0
1.0
0.0
ppm (t1)

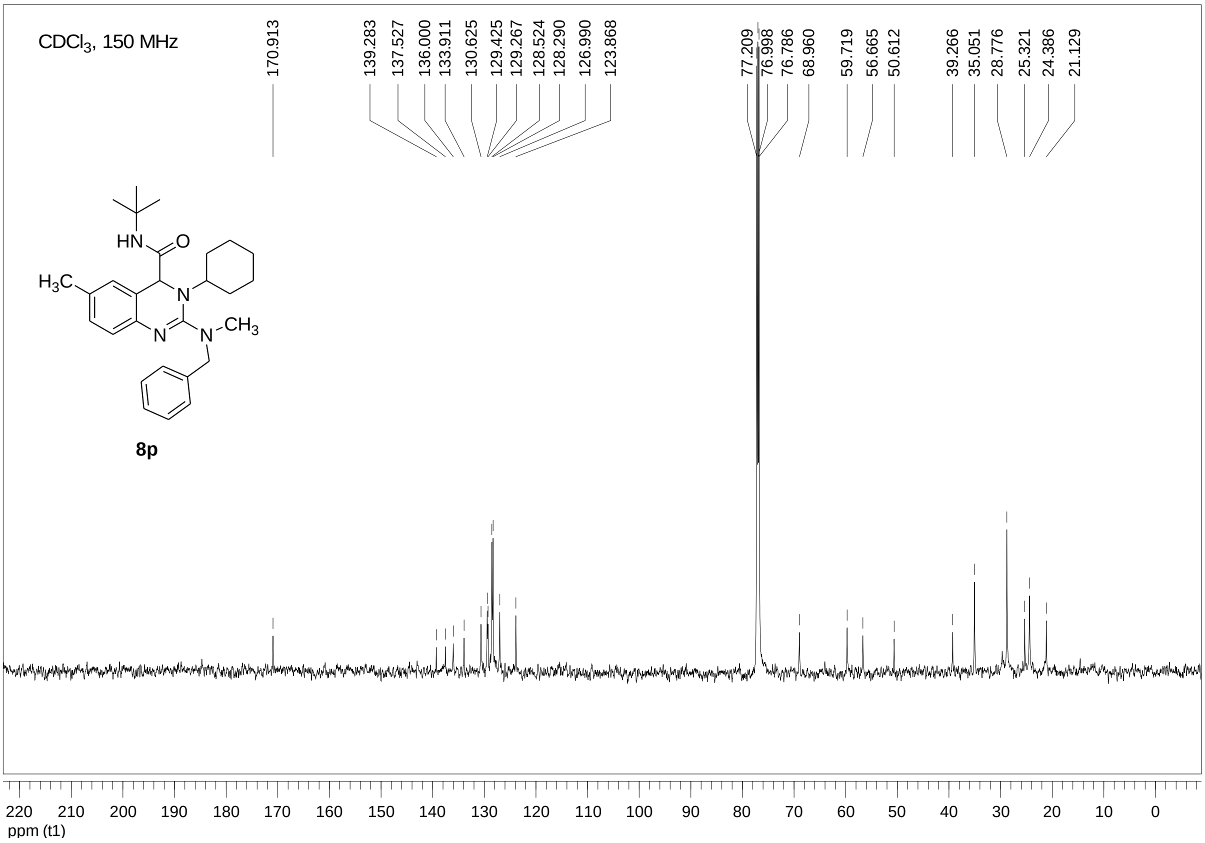
CDCl3, 150 MHz
170.913
139.283
137.527
136.000
133.911
130.625
129.425
129.267
128.524
128.290
126.990
123.868
77.209
76.998
76.786
68.960
59.719
56.665
50.612
39.266
35.051
28.776
25.321
24.386
21.129
HN
O
H3C
N
N
N
CH3
8p
220
210
200
190
180
170
160
150
140
130
120
110
100
90
80
70
60
50
40
30
20
10
0
ppm (t1)

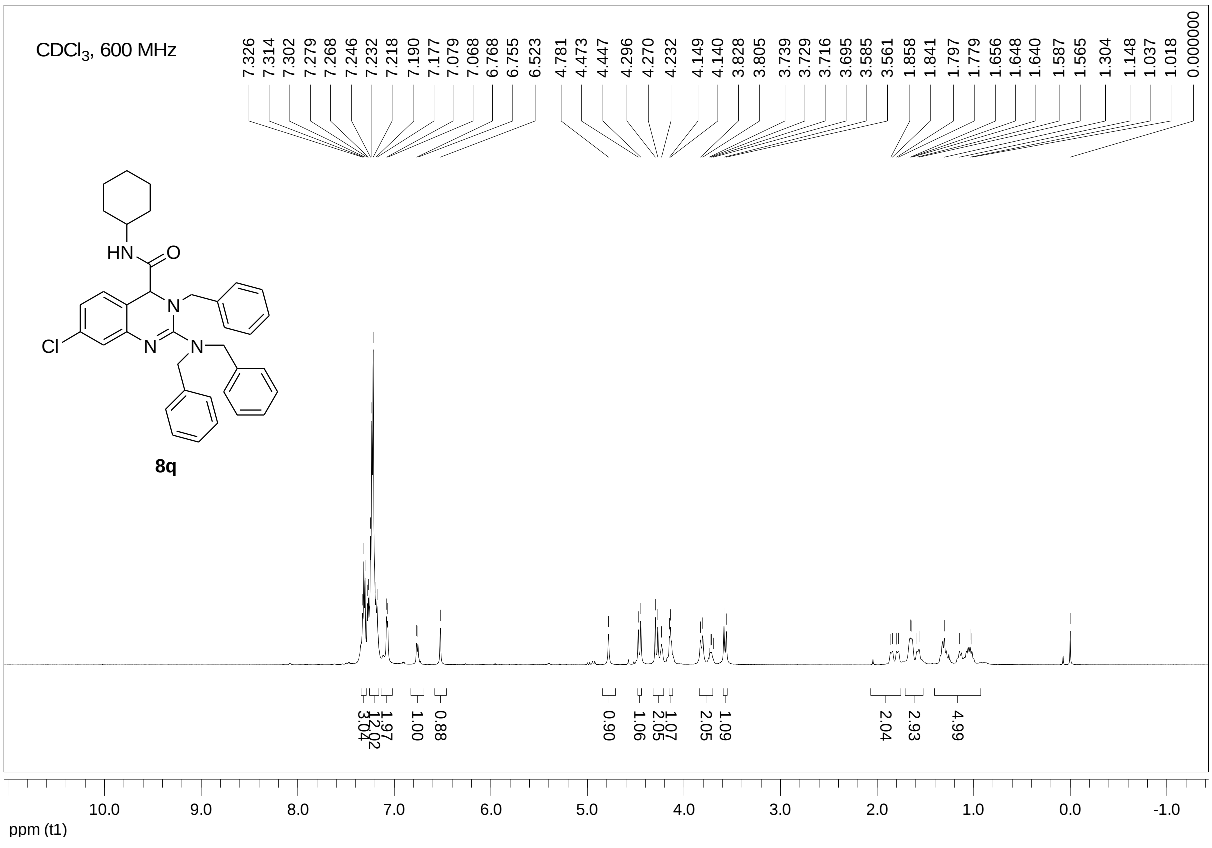
CDCl3, 600 MHz
7.326 7.314 7.302 7.279 7.268 7.246 7.232 7.218 7.190 7.177 7.079 7.068 6.768 6.755 6.523 4.781 4.473 4.447 4.296 4.270 4.232 4.149 4.140 3.828 3.805 3.739 3.729 3.716 3.695 3.585 3.561 1.858 1.841 1.797 1.779 1.656 1.648 1.640 1.587 1.565 1.304 1.148 1.037 1.018 0.000000
HN
O
N
Cl
N
N
8q
1.97 1.00 0.88 0.90 1.06 2.05 1.07 2.05 1.09 2.04 2.93 4.99
10.0 9.0 8.0 7.0 6.0 5.0 4.0 3.0 2.0 1.0 0.0 -1.0
ppm (t1)

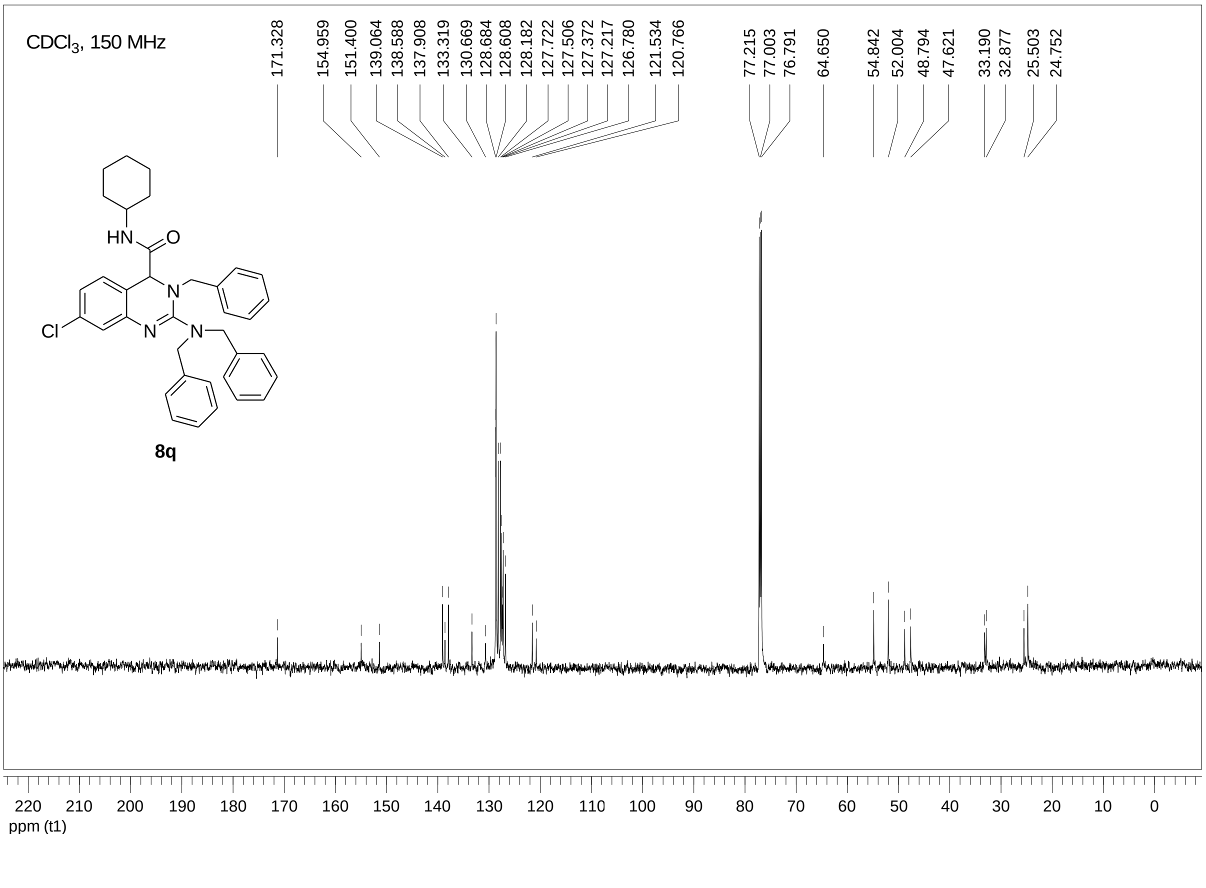
CDCl3, 150 MHz
171.328 154.959 151.400 139.064 138.588 137.908 133.319 130.669 128.684 128.608 128.182 127.722 127.506 127.372 127.217 126.780 121.534 120.766 77.215 77.003 76.791 64.650 54.842 52.004 48.794 47.621 33.190 32.877 25.503 24.752
HN
O
N
Cl
N
N
8q
220 210 200 190 180 170 160 150 140 130 120 110 100 90 80 70 60 50 40 30 20 10 0
ppm (t1)

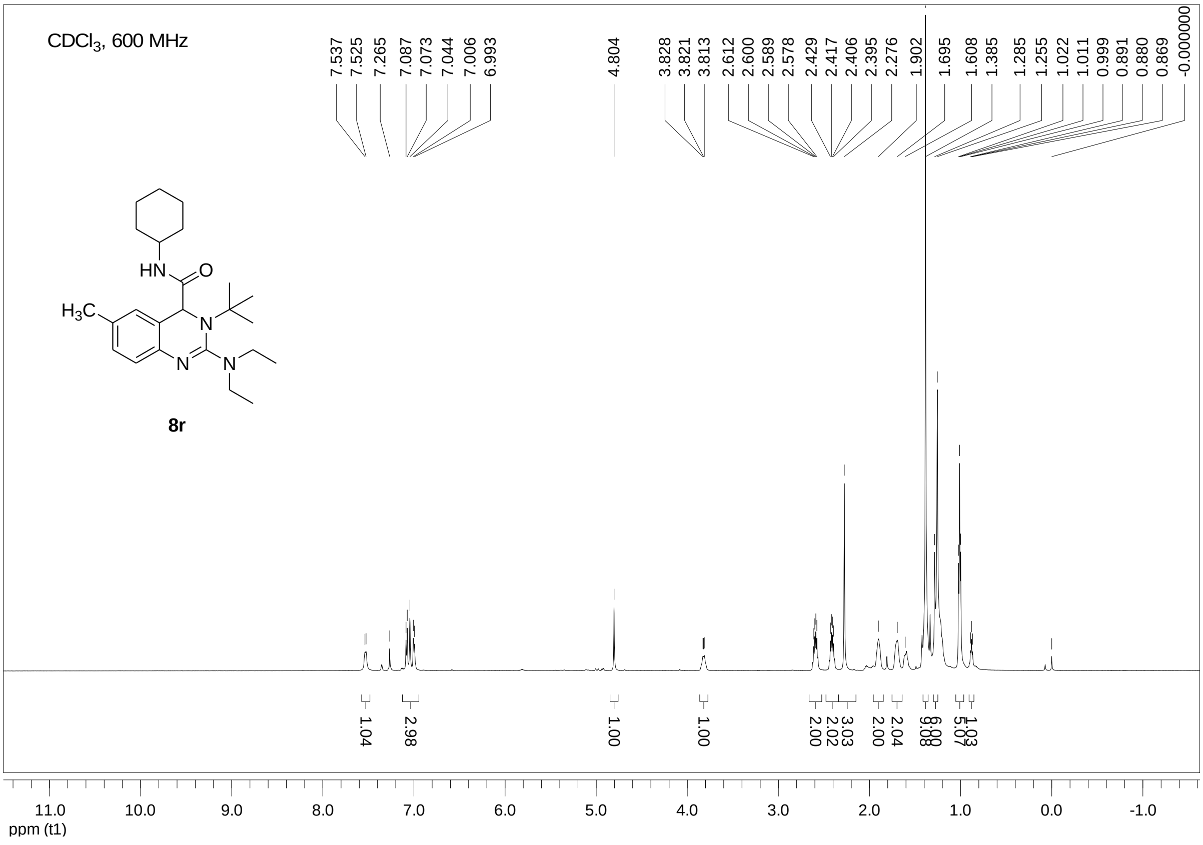
CDCl3, 600 MHz
8r
7.537 7.525 7.265 7.087 7.073 7.044 7.006 6.993 4.804 3.828 3.821 3.813 2.612 2.600 2.589 2.578 2.429 2.417 2.406 2.395 2.276 1.902 1.695 1.608 1.385 1.285 1.255 1.022 1.011 0.999 0.891 0.880 0.869 -0.000000
1.04 2.98 1.00 1.00 2.00 2.02 3.03 2.00 2.04
ppm (t1)

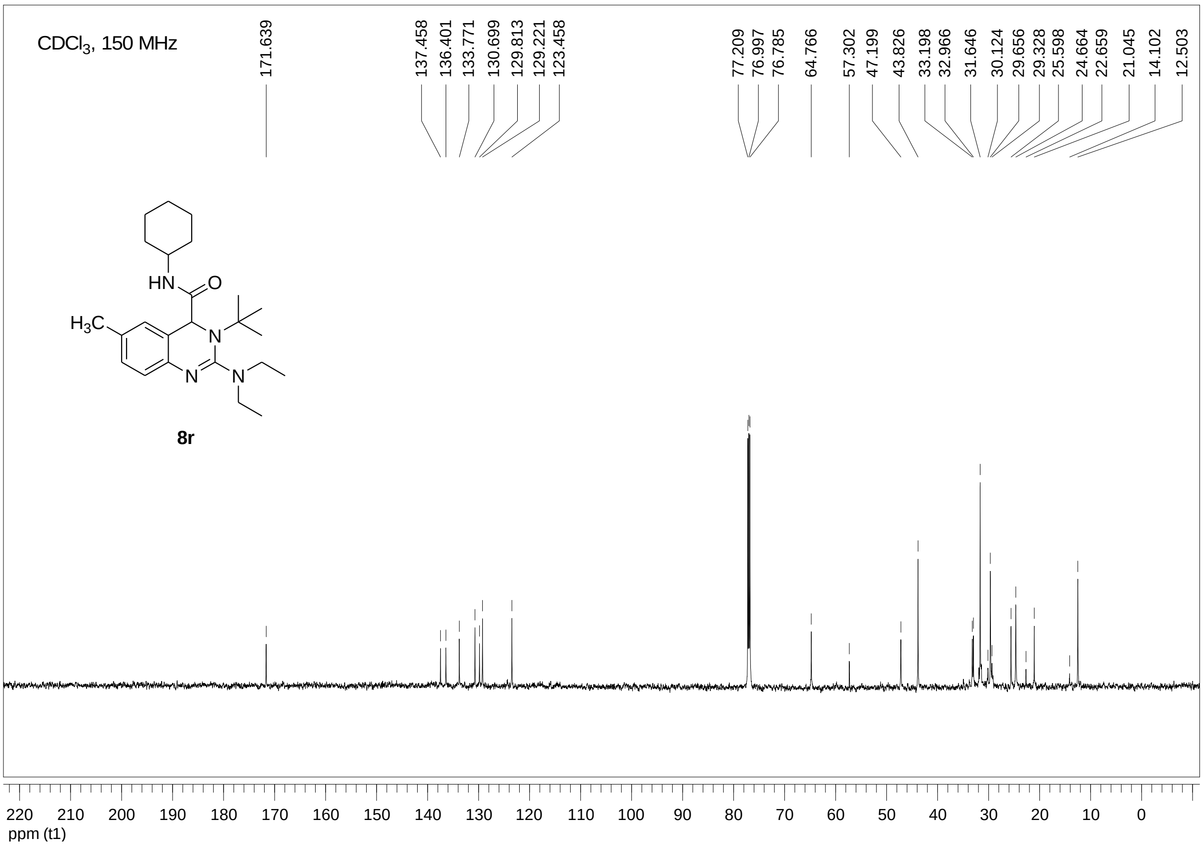
CDCl3, 150 MHz
8r
171.639 137.458 136.401 133.771 130.699 129.813 129.221 123.458 77.209 76.997 76.785 64.766 57.302 47.199 43.826 33.198 32.966 31.646 30.124 29.656 29.328 25.598 24.664 22.659 21.045 14.102 12.503
ppm (t1)

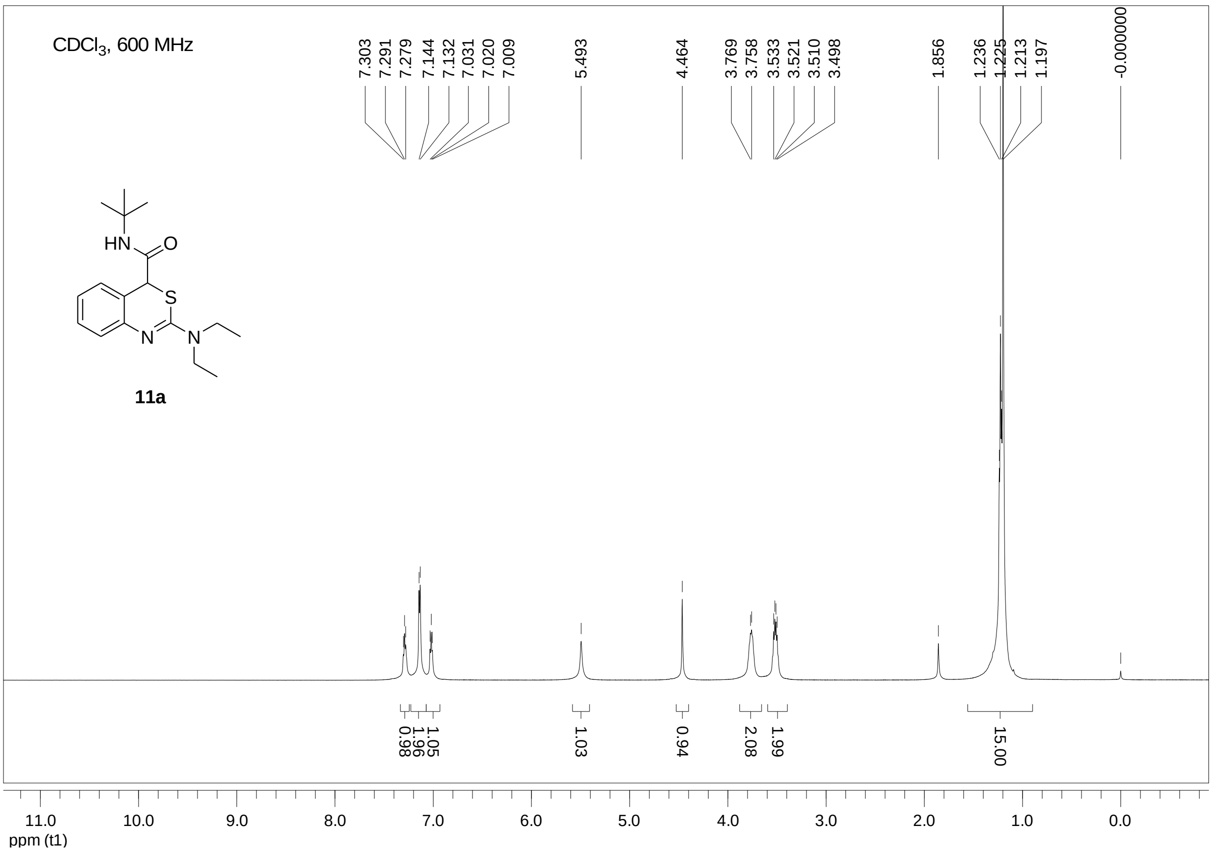
CDCl3, 600 MHz
7.303 7.291 7.279 7.144 7.132 7.031 7.020 7.009
5.493
4.464
3.769 3.758 3.533 3.521 3.510 3.498
1.856
1.236 1.225 1.213 1.197
-0.000000
HN
O
S
N
N
11a
0.98 1.96 1.05
1.03
0.94
2.08
1.99
15.00
11.0 10.0 9.0 8.0 7.0 6.0 5.0 4.0 3.0 2.0 1.0 0.0
ppm (t1)

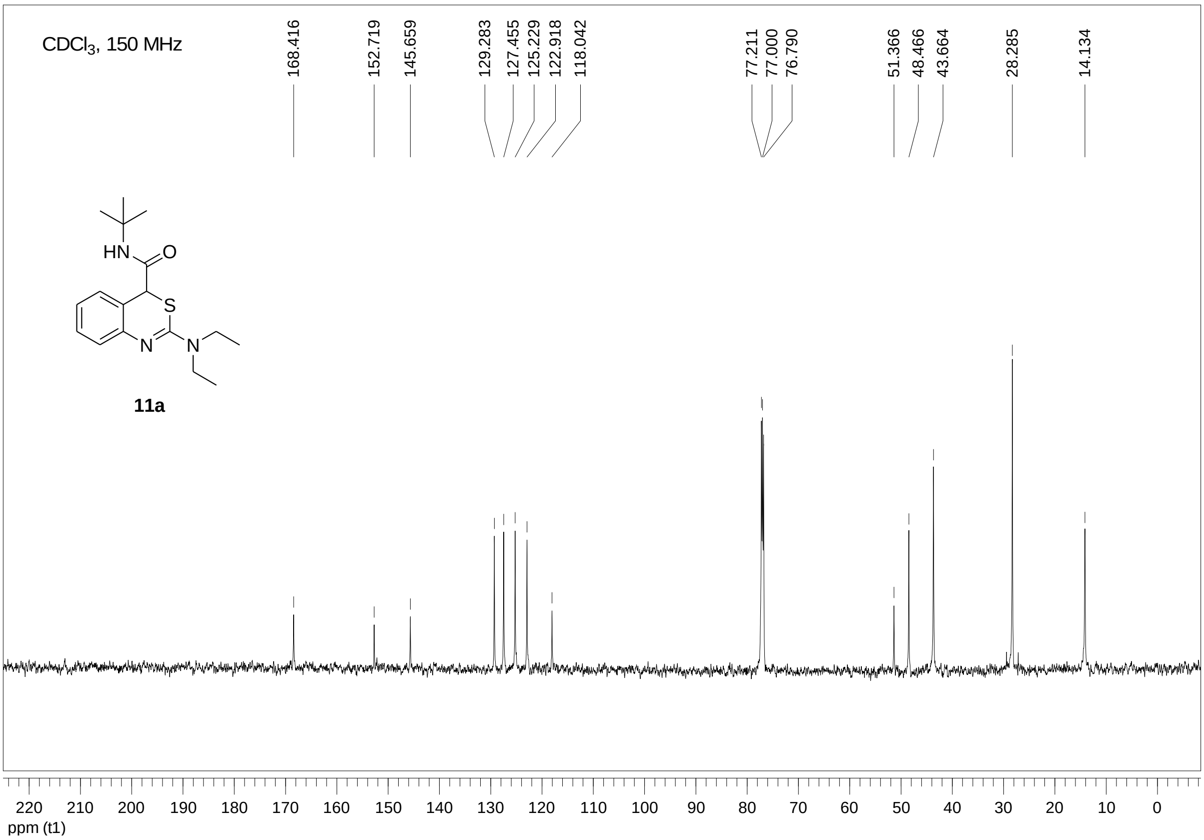
CDCl3, 150 MHz
168.416
152.719
145.659
129.283 127.455 125.229 122.918 118.042
77.211 77.000 76.790
51.366 48.466 43.664
28.285
14.134
HN
O
S
N
N
11a
220 210 200 190 180 170 160 150 140 130 120 110 100 90 80 70 60 50 40 30 20 10 0
ppm (t1)

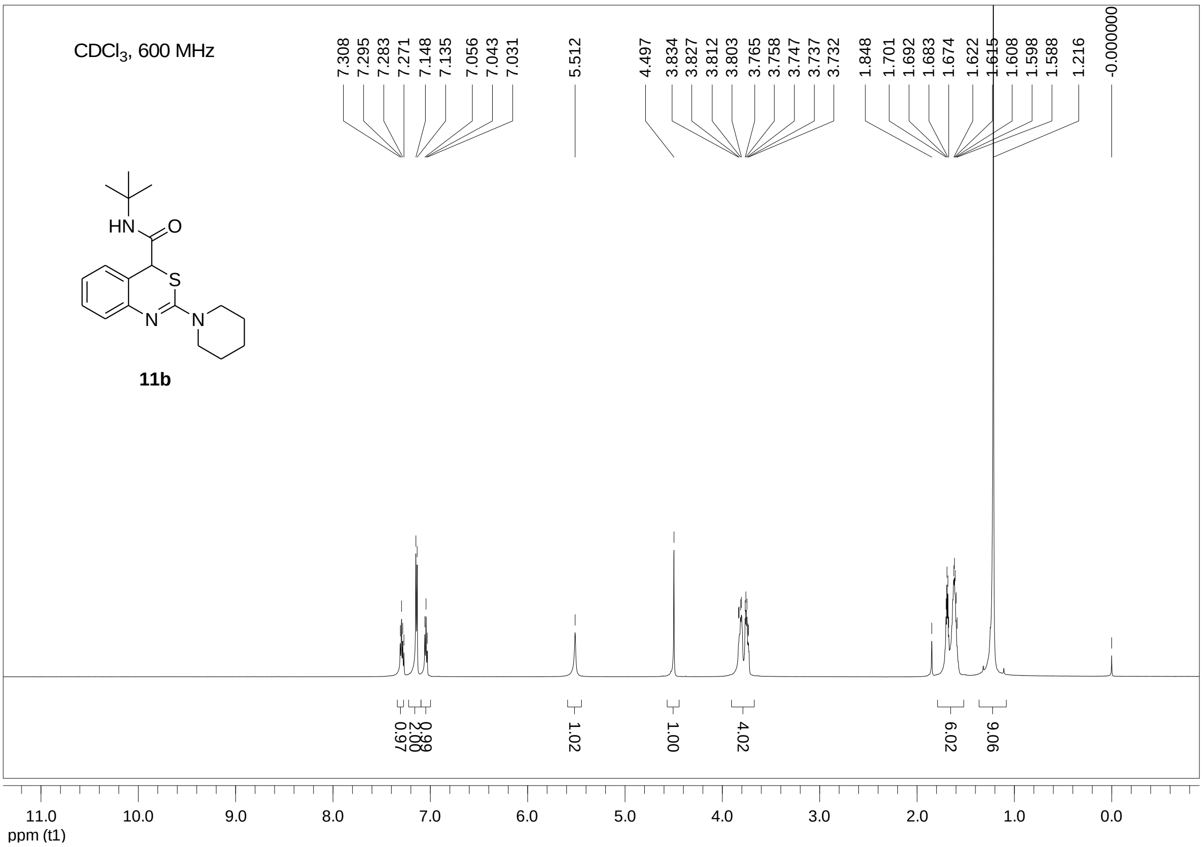
CDCl3, 600 MHz
7.308
7.295
7.283
7.271
7.148
7.135
7.056
7.043
7.031
5.512
4.497
3.834
3.827
3.812
3.803
3.765
3.758
3.747
3.737
3.732
1.848
1.701
1.692
1.683
1.674
1.622
1.615
1.608
1.598
1.588
1.216
-0.000000
HN
O
S
N
N
11b
0.97
2.00
0.99
1.02
1.00
4.02
6.02
9.06
11.0
10.0
9.0
8.0
7.0
6.0
5.0
4.0
3.0
2.0
1.0
0.0
ppm (t1)

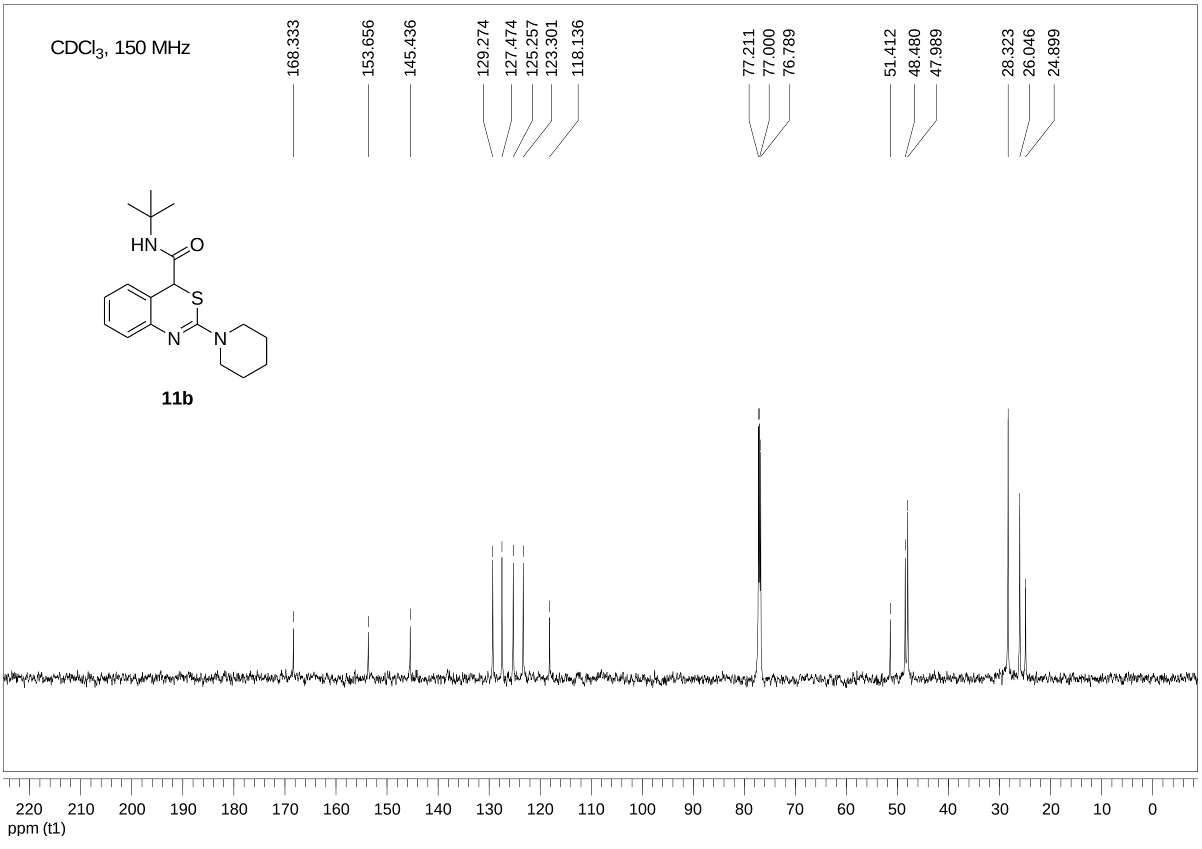
CDCl3, 150 MHz
168.333
153.656
145.436
129.274
127.474
125.257
123.301
118.136
77.211
77.000
76.789
51.412
48.480
47.989
28.323
26.046
24.899
HN
O
S
N
N
11b
220
210
200
190
180
170
160
150
140
130
120
110
100
90
80
70
60
50
40
30
20
10
0
ppm (t1)

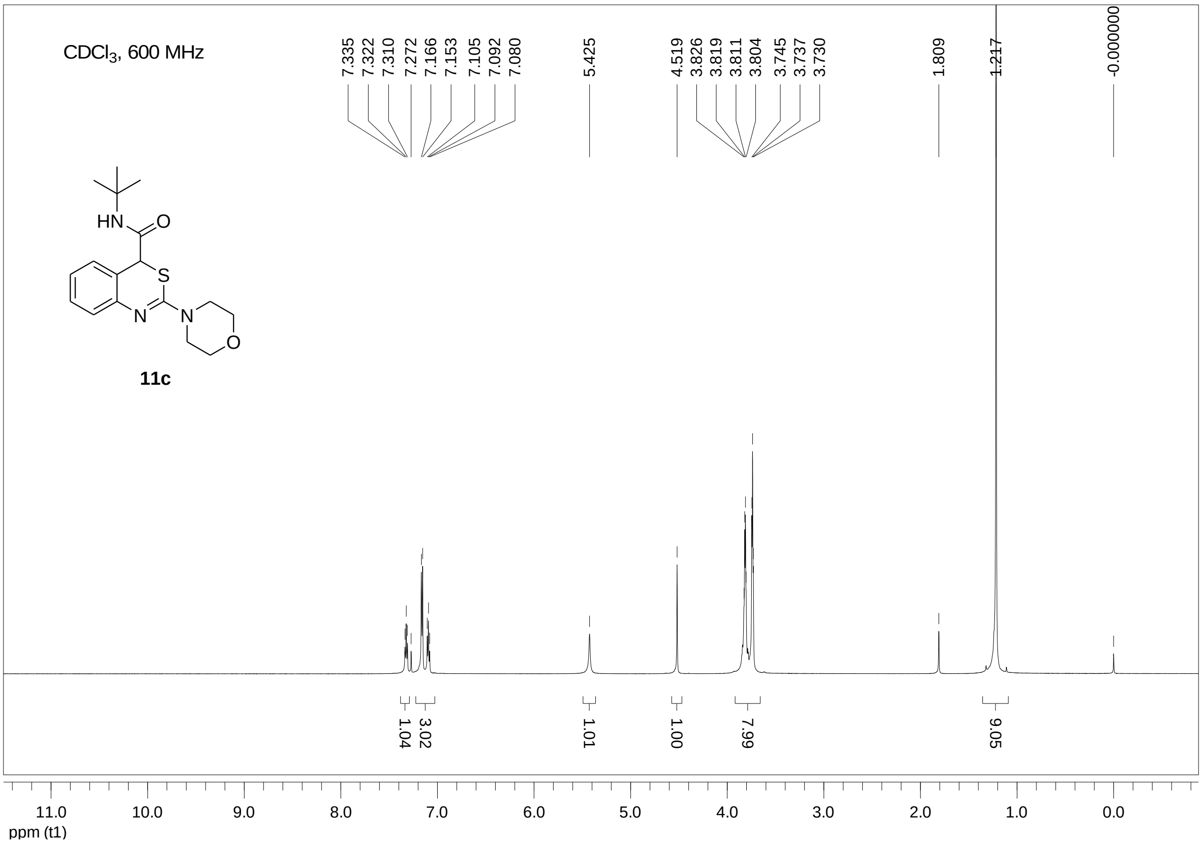
CDCl3, 600 MHz
7.335
7.322
7.310
7.272
7.166
7.153
7.105
7.092
7.080
5.425
4.519
3.826
3.819
3.811
3.804
3.745
3.737
3.730
1.809
1.217
-0.000000
HN
O
S
N
N
O
11c
1.04
3.02
1.01
1.00
7.99
9.05
11.0
10.0
9.0
8.0
7.0
6.0
5.0
4.0
3.0
2.0
1.0
0.0
ppm (t1)

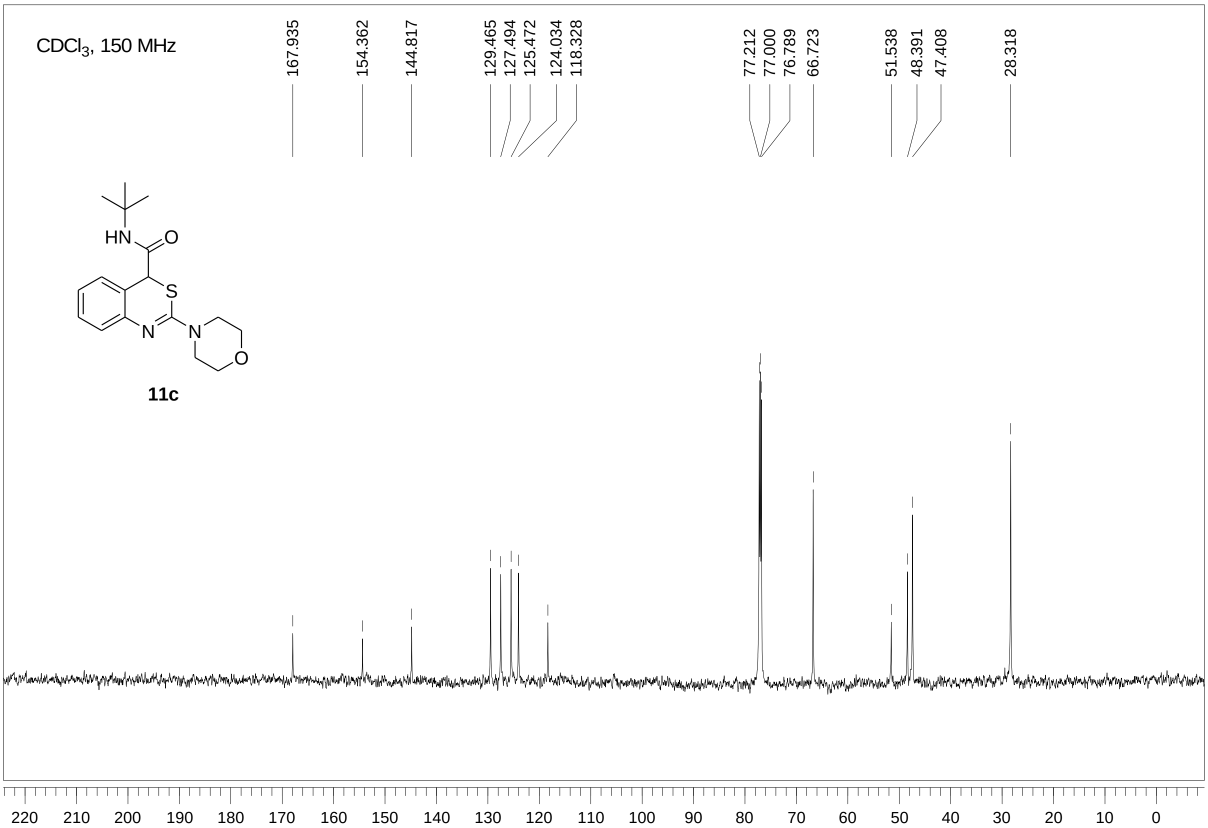
CDCl3, 150 MHz
167.935
154.362
144.817
129.465
127.494
125.472
124.034
118.328
77.212
77.000
76.789
66.723
51.538
48.391
47.408
28.318
HN
O
S
N
N
O
11c
220
210
200
190
180
170
160
150
140
130
120
110
100
90
80
70
60
50
40
30
20
10
0
ppm (t1)

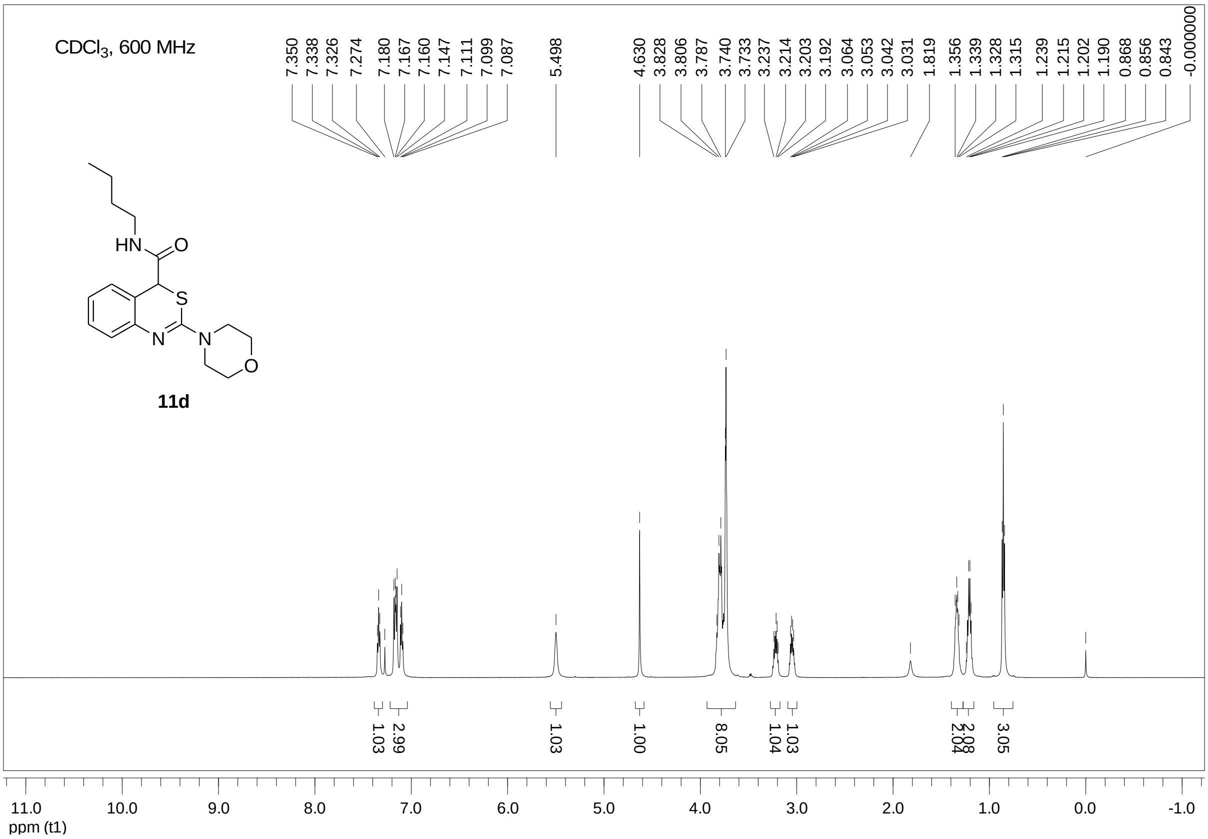
CDCl3, 600 MHz
7.350 7.338 7.326 7.274 7.180 7.167 7.160 7.147 7.111 7.099 7.087
5.498
4.630 3.828 3.806 3.787 3.740 3.733 3.237 3.214 3.203 3.192 3.064 3.053 3.042 3.031 1.819 1.356 1.339 1.328 1.315 1.239 1.215 1.202 1.190 0.868 0.856 0.843 -0.000000
HN
O
S
N
N
O
11d
1.03 2.99 1.03 1.00 8.05 1.04 1.03 2.04 2.08 3.05
11.0 10.0 9.0 8.0 7.0 6.0 5.0 4.0 3.0 2.0 1.0 0.0 -1.0
ppm (t1)

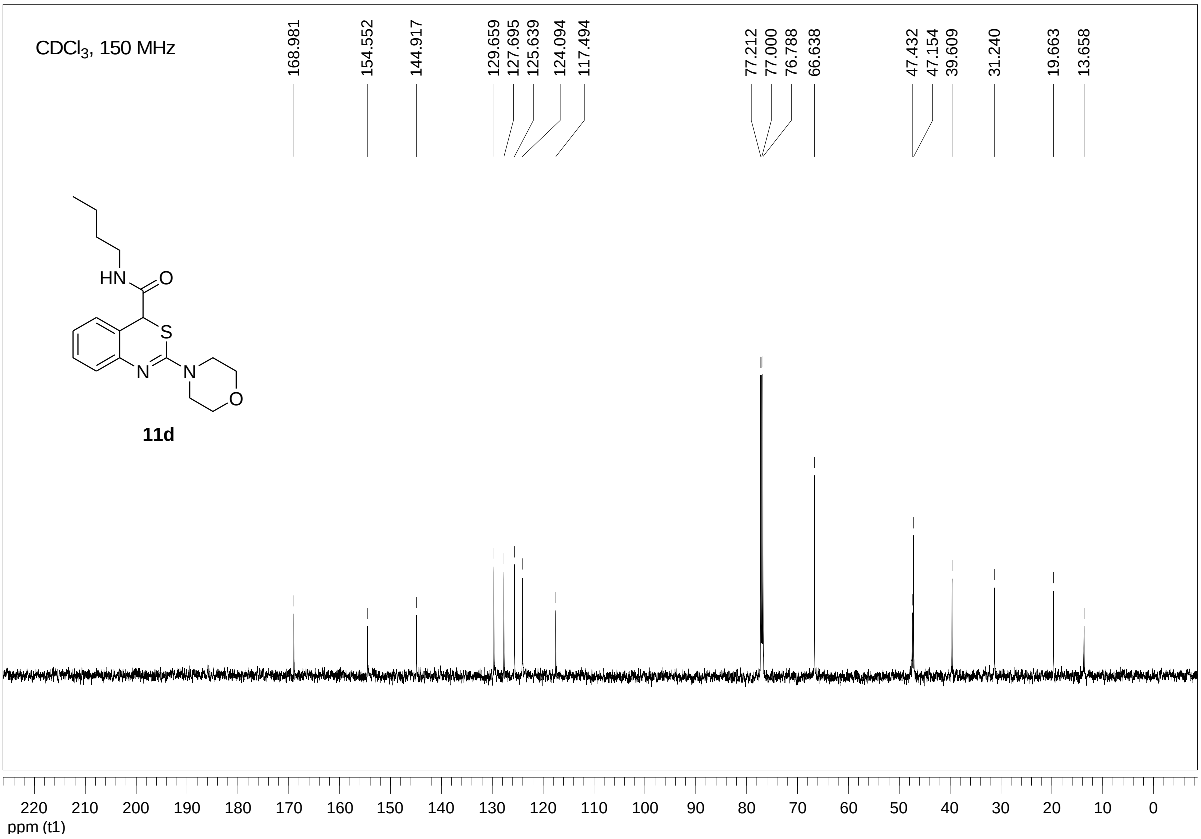
CDCl3, 150 MHz
168.981
154.552
144.917
129.659 127.695 125.639 124.094 117.494
77.212 77.000 76.788 66.638
47.432 47.154 39.609
31.240
19.663 13.658
HN
O
S
N
N
O
11d
220 210 200 190 180 170 160 150 140 130 120 110 100 90 80 70 60 50 40 30 20 10 0
ppm (t1)

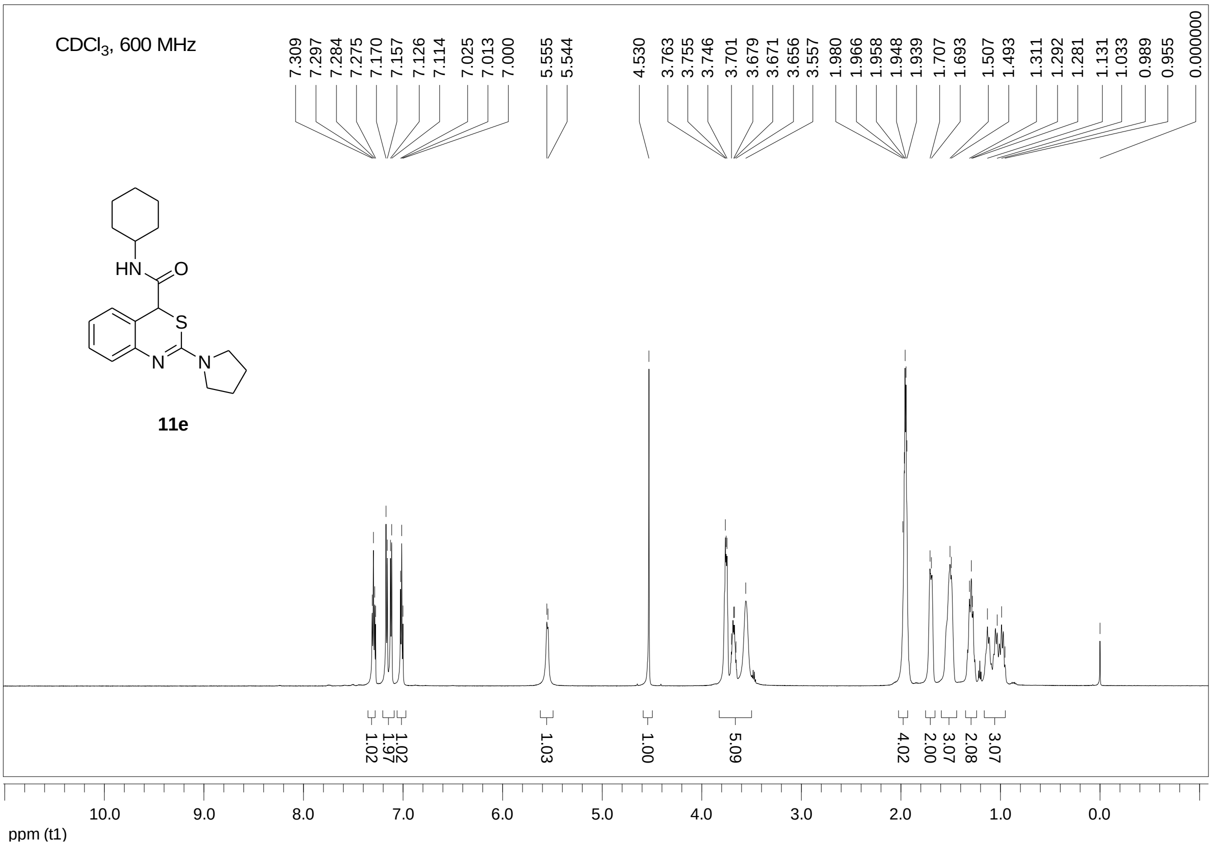
CDCl3, 600 MHz
7.309
7.297
7.284
7.275
7.170
7.157
7.126
7.114
7.025
7.013
7.000
5.555
5.544
4.530
3.763
3.755
3.746
3.701
3.679
3.671
3.656
3.557
1.980
1.966
1.958
1.948
1.939
1.707
1.693
1.507
1.493
1.311
1.292
1.281
1.131
1.033
0.989
0.955
0.000000
HN
O
S
N
N
11e
1.02
1.97
1.02
1.03
1.00
5.09
4.02
2.00
3.07
2.08
3.07
10.0
9.0
8.0
7.0
6.0
5.0
4.0
3.0
2.0
1.0
0.0
ppm (t1)

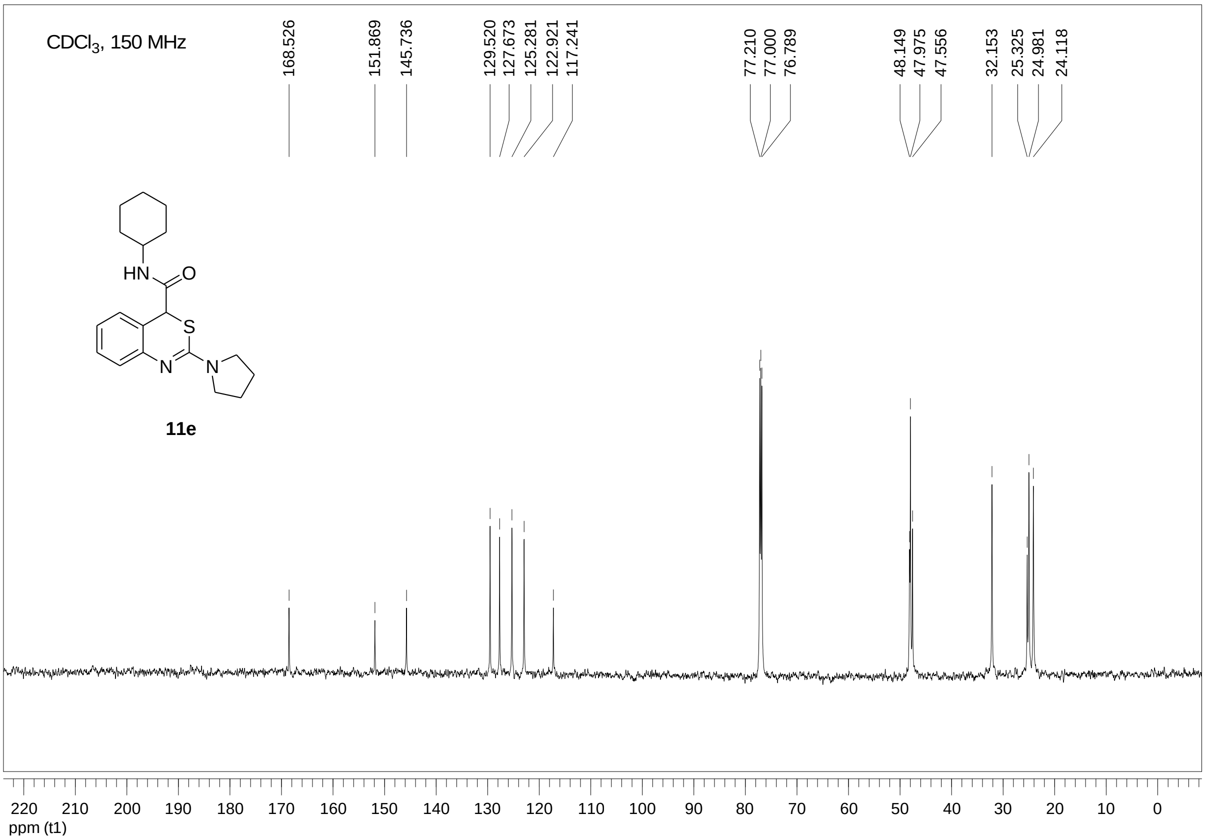
CDCl3, 150 MHz
168.526
151.869
145.736
129.520
127.673
125.281
122.921
117.241
77.210
77.000
76.789
48.149
47.975
47.556
32.153
25.325
24.981
24.118
HN
O
S
N
N
11e
220
210
200
190
180
170
160
150
140
130
120
110
100
90
80
70
60
50
40
30
20
10
0
ppm (t1)

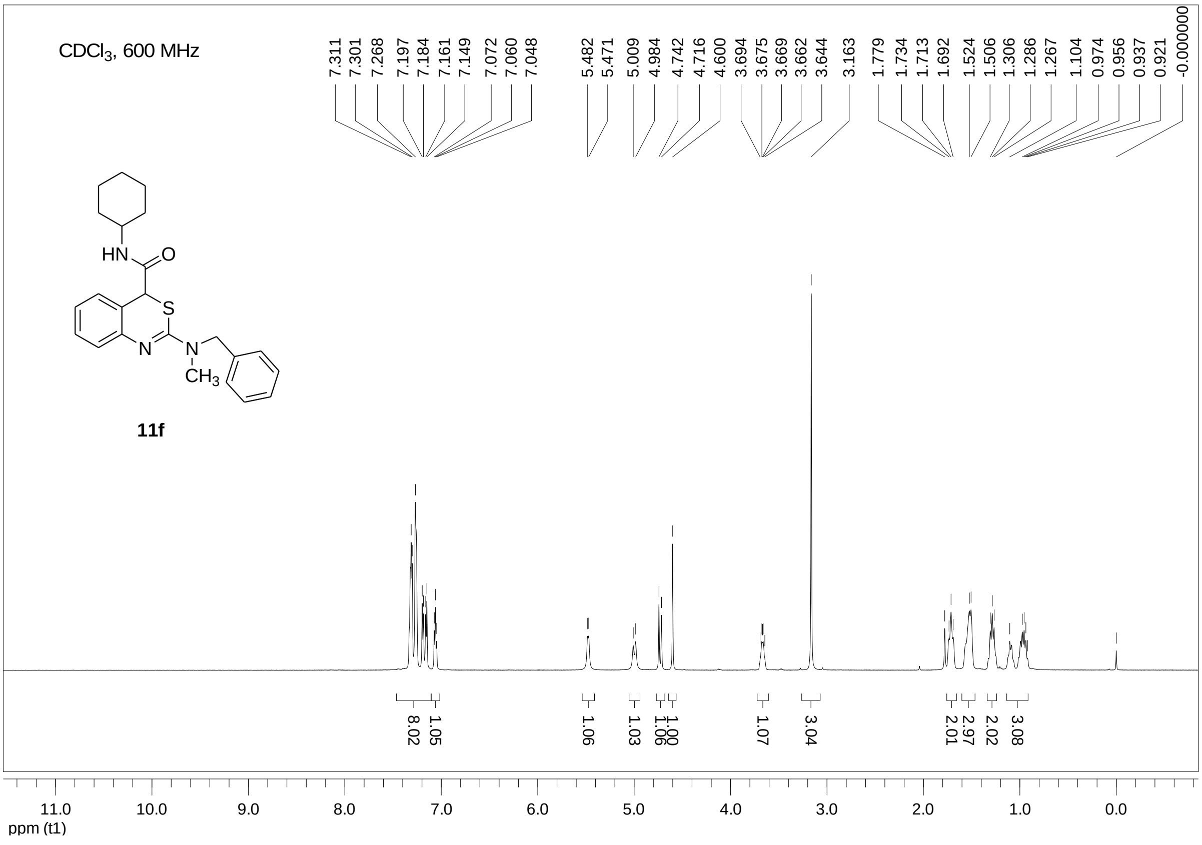
CDCl$_3$, 600 MHz
7.311
7.301
7.268
7.197
7.184
7.161
7.149
7.072
7.060
7.048
5.482
5.471
5.009
4.984
4.742
4.716
4.600
3.694
3.675
3.669
3.662
3.644
3.163
1.779
1.734
1.713
1.692
1.524
1.506
1.306
1.286
1.267
1.104
0.974
0.956
0.937
0.921
-0.000000
HN
O
S
N
N
CH$_3$
11f
8.02
1.05
1.06
1.03
1.06
1.00
1.07
3.04
2.01
2.97
2.02
3.08
11.0
10.0
9.0
8.0
7.0
6.0
5.0
4.0
3.0
2.0
1.0
0.0
ppm (t1)

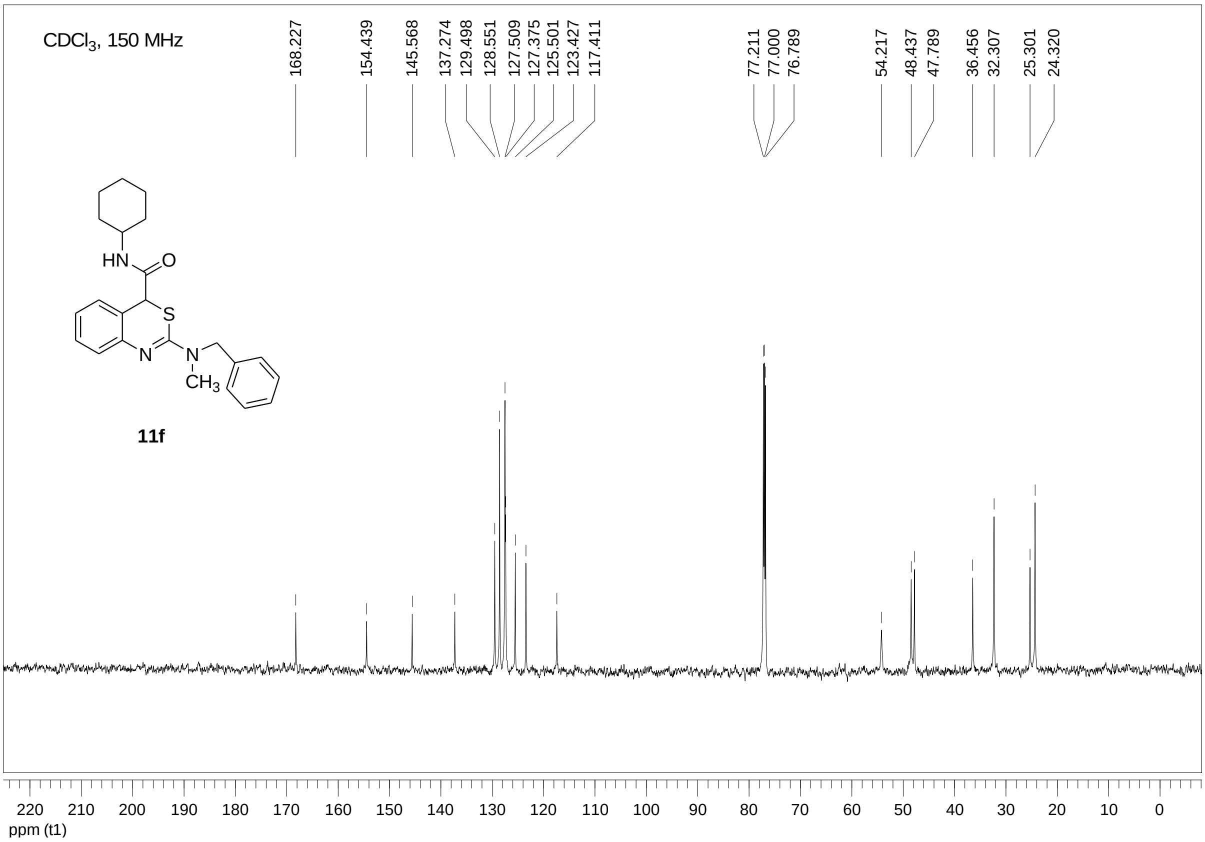
CDCl$_3$, 150 MHz
168.227
154.439
145.568
137.274
129.498
128.551
127.509
127.375
125.501
123.427
117.411
77.211
77.000
76.789
54.217
48.437
47.789
36.456
32.307
25.301
24.320
HN
O
S
N
N
CH$_3$
11f
220
210
200
190
180
170
160
150
140
130
120
110
100
90
80
70
60
50
40
30
20
10
0
ppm (t1)

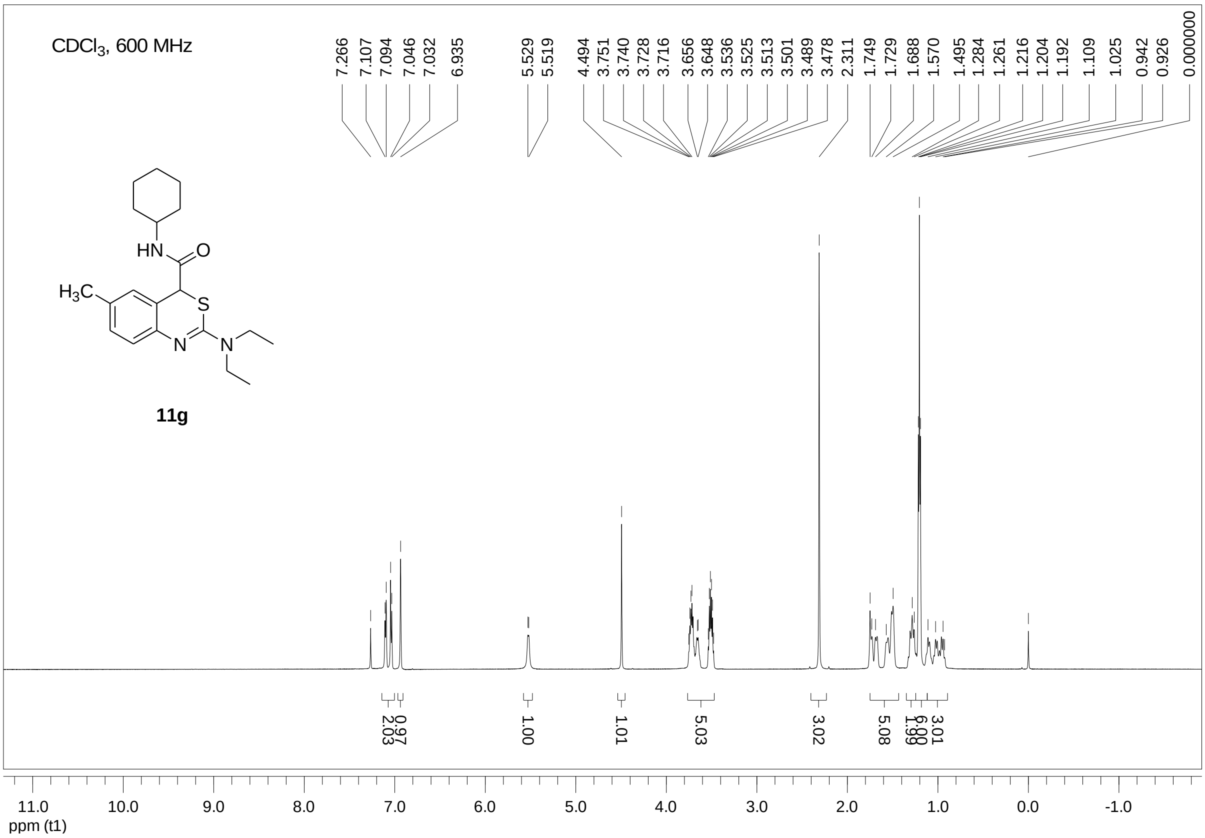
CDCl3, 600 MHz
7.266
7.107
7.094
7.046
7.032
6.935
5.529
5.519
4.494
3.751
3.740
3.728
3.716
3.656
3.648
3.536
3.525
3.513
3.501
3.489
3.478
2.311
1.749
1.729
1.688
1.570
1.495
1.284
1.261
1.216
1.204
1.192
1.109
1.025
0.942
0.926
0.000000
HN
O
H3C
S
N
N
11g
2.03
0.97
1.00
1.01
5.03
3.02
5.08
3.01
11.0
10.0
9.0
8.0
7.0
6.0
5.0
4.0
3.0
2.0
1.0
0.0
-1.0
ppm (t1)

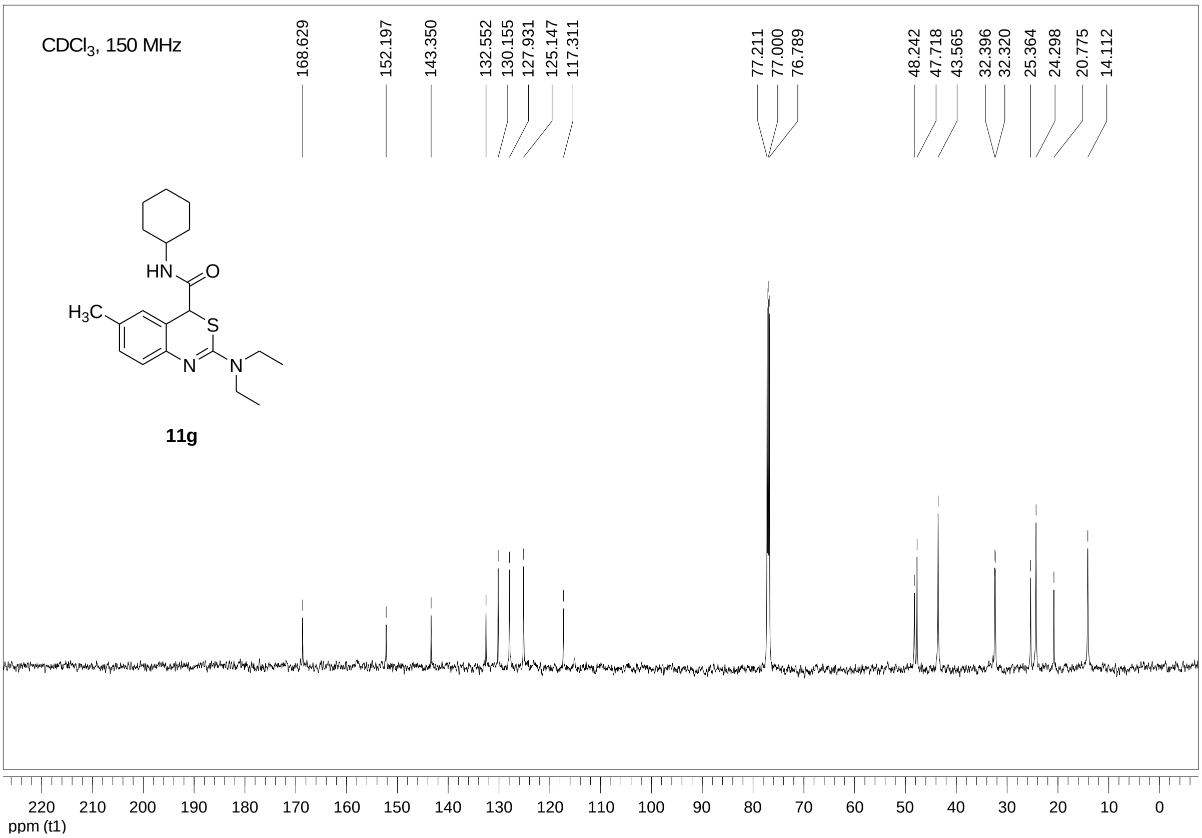
CDCl3, 150 MHz
168.629
152.197
143.350
132.552
130.155
127.931
125.147
117.311
77.211
77.000
76.789
48.242
47.718
43.565
32.396
32.320
25.364
24.298
20.775
14.112
HN
O
H3C
S
N
N
11g
220
210
200
190
180
170
160
150
140
130
120
110
100
90
80
70
60
50
40
30
20
10
0
ppm (t1)

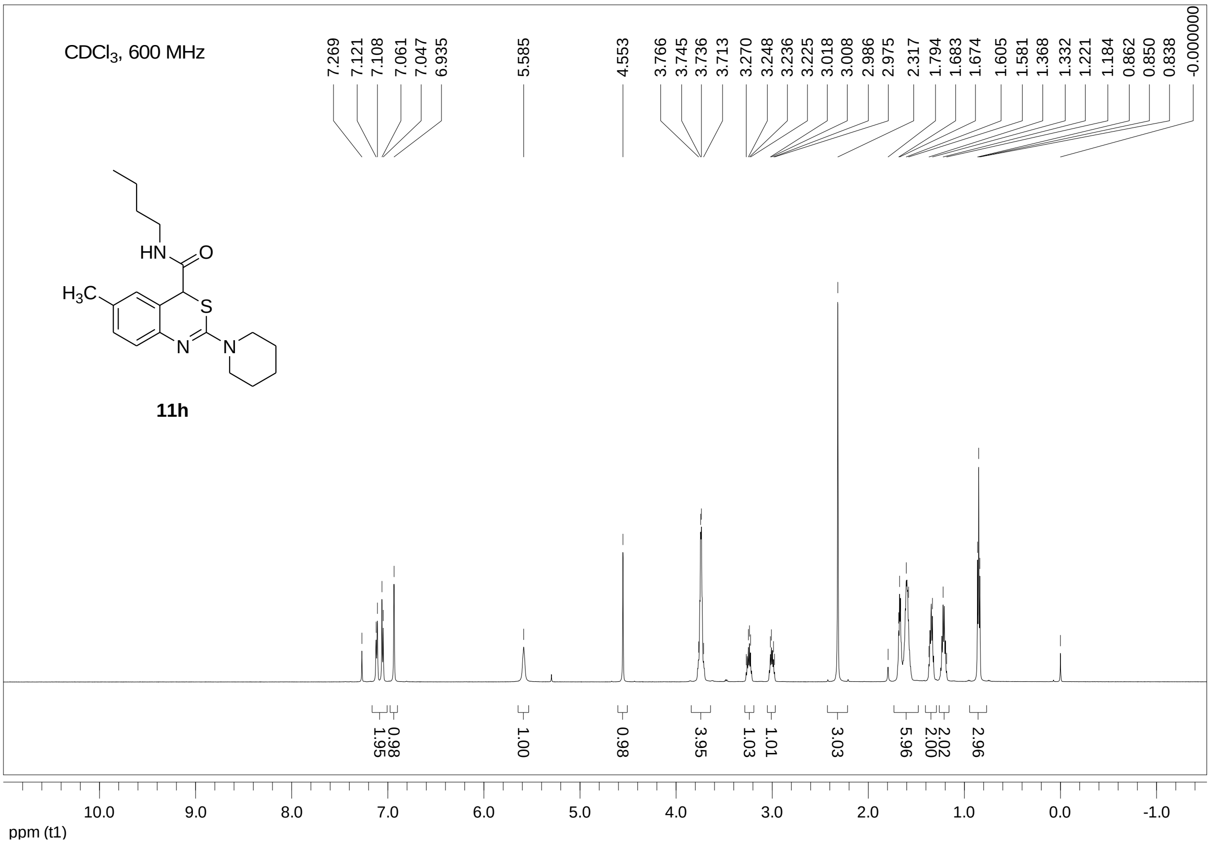
CDCl3, 600 MHz
7.269
7.121
7.108
7.061
7.047
6.935
5.585
4.553
3.766
3.745
3.736
3.713
3.270
3.248
3.236
3.225
3.018
3.008
2.986
2.975
2.317
1.794
1.683
1.674
1.605
1.581
1.368
1.332
1.221
1.184
0.862
0.850
0.838
-0.000000
HN
O
H3C
S
N
N
11h
1.95
0.98
1.00
0.98
3.95
1.03
1.01
3.03
5.96
2.00
2.02
2.96
10.0
9.0
8.0
7.0
6.0
5.0
4.0
3.0
2.0
1.0
0.0
-1.0
ppm (t1)

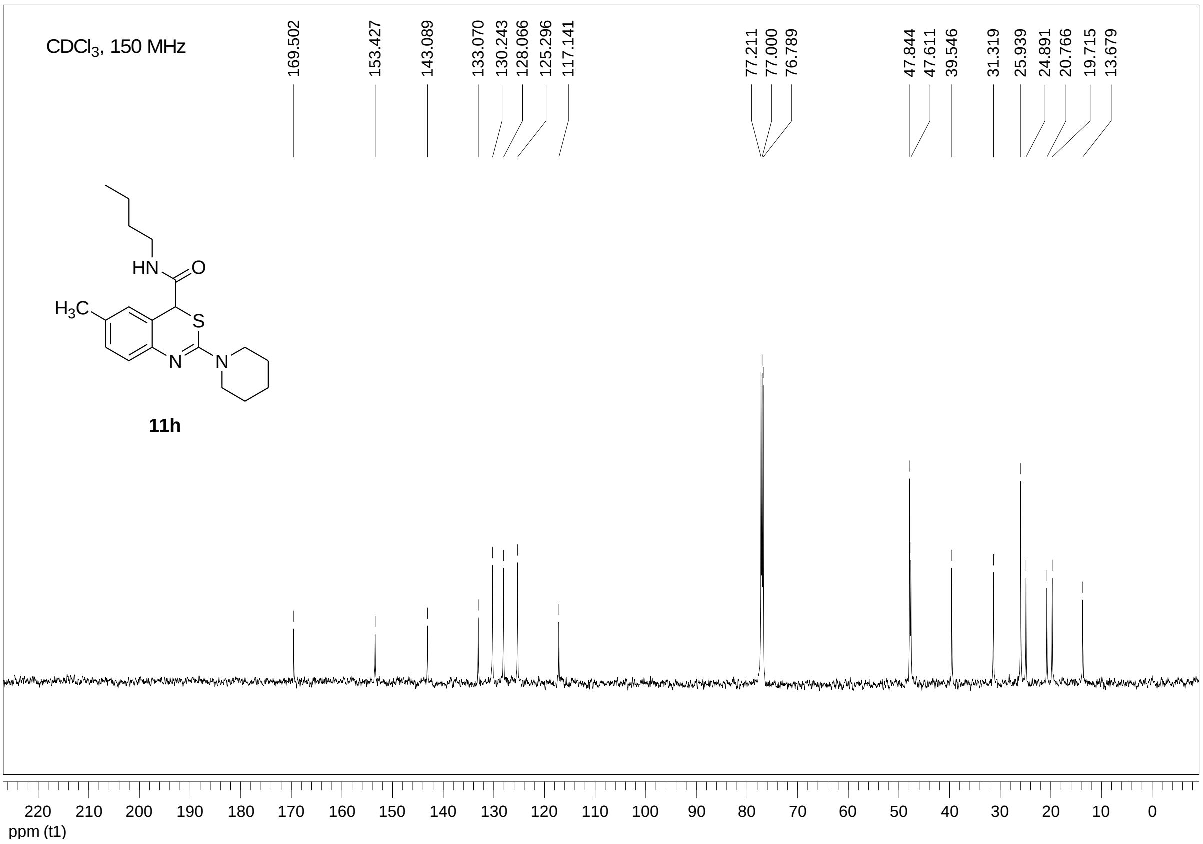
CDCl3, 150 MHz
169.502
153.427
143.089
133.070
130.243
128.066
125.296
117.141
77.211
77.000
76.789
47.844
47.611
39.546
31.319
25.939
24.891
20.766
19.715
13.679
HN
O
H3C
S
N
N
11h
220 210 200 190 180 170 160 150 140 130 120 110 100 90 80 70 60 50 40 30 20 10 0
ppm (t1)

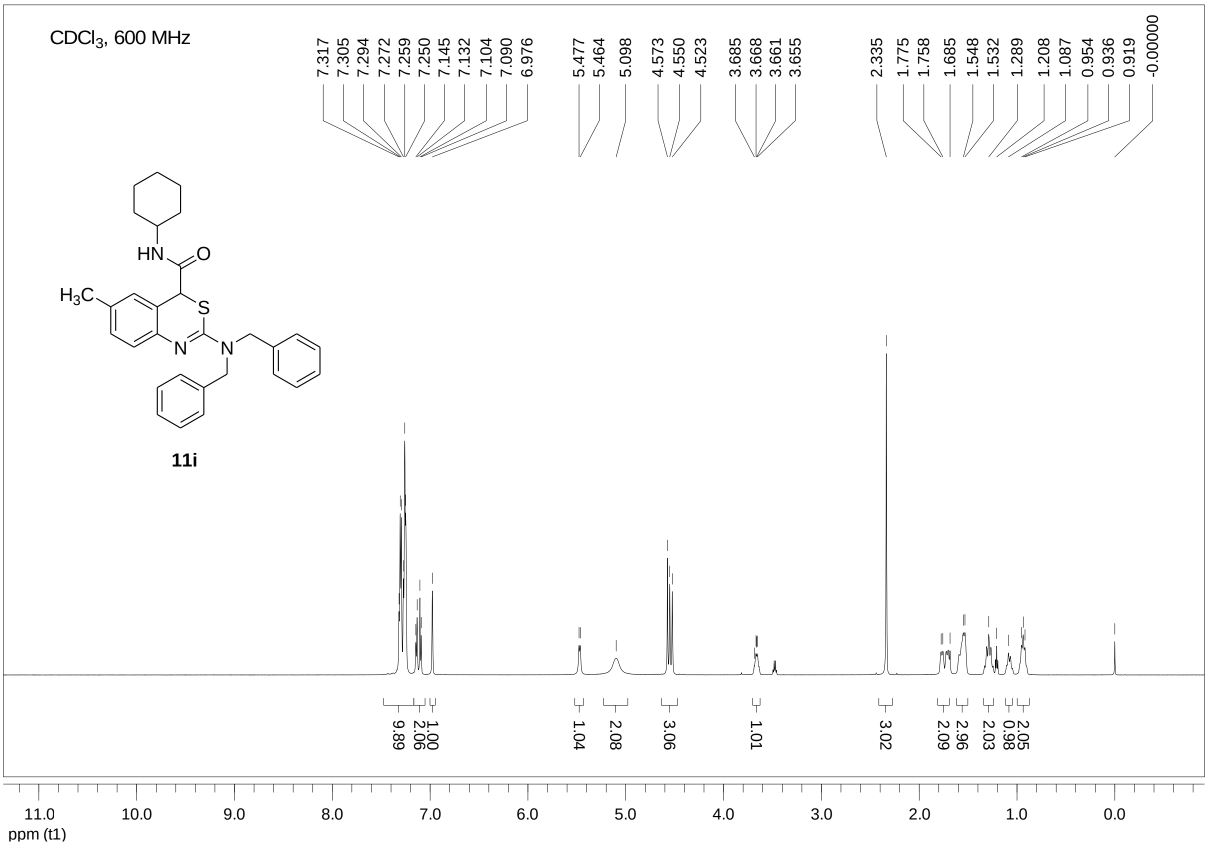
CDCl3, 600 MHz
7.317 7.305 7.294 7.272 7.259 7.250 7.145 7.132 7.104 7.090 6.976
5.477 5.464 5.098
4.573 4.550 4.523
3.685 3.668 3.661 3.655
2.335 1.775 1.758 1.685 1.548 1.532 1.289 1.208 1.087 0.954 0.936 0.919 -0.00000
HN O
H3C
S
N N
11i
9.89 2.06 1.00 1.04 2.08 3.06 1.01 3.02 2.09 2.96 2.03 0.98 2.05
11.0 10.0 9.0 8.0 7.0 6.0 5.0 4.0 3.0 2.0 1.0 0.0
ppm (t1)

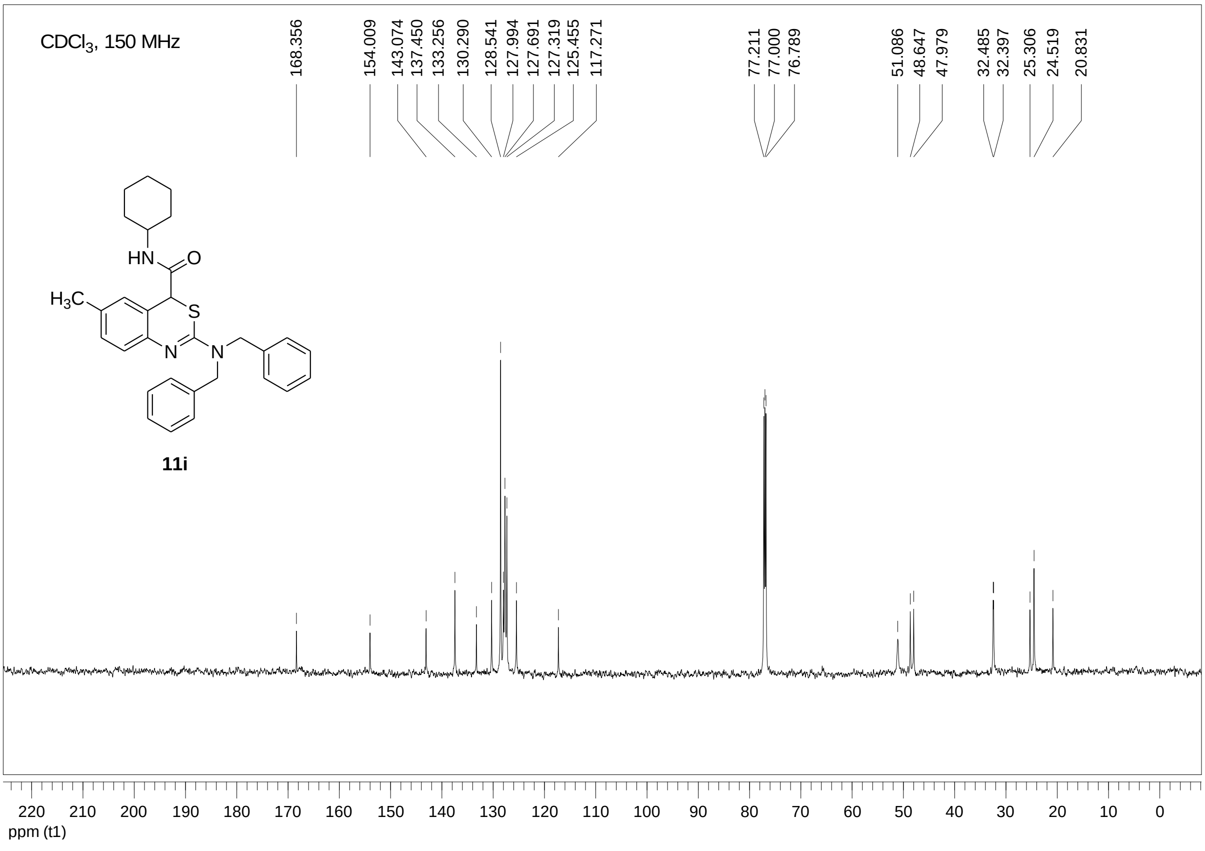
CDCl3, 150 MHz
168.356
154.009 143.074 137.450 133.256 130.290 128.541 127.994 127.691 127.319 125.455 117.271
77.211 77.000 76.789
51.086 48.647 47.979
32.485 32.397 25.306 24.519 20.831
HN O
H3C
S
N N
11i
220 210 200 190 180 170 160 150 140 130 120 110 100 90 80 70 60 50 40 30 20 10 0
ppm (t1)

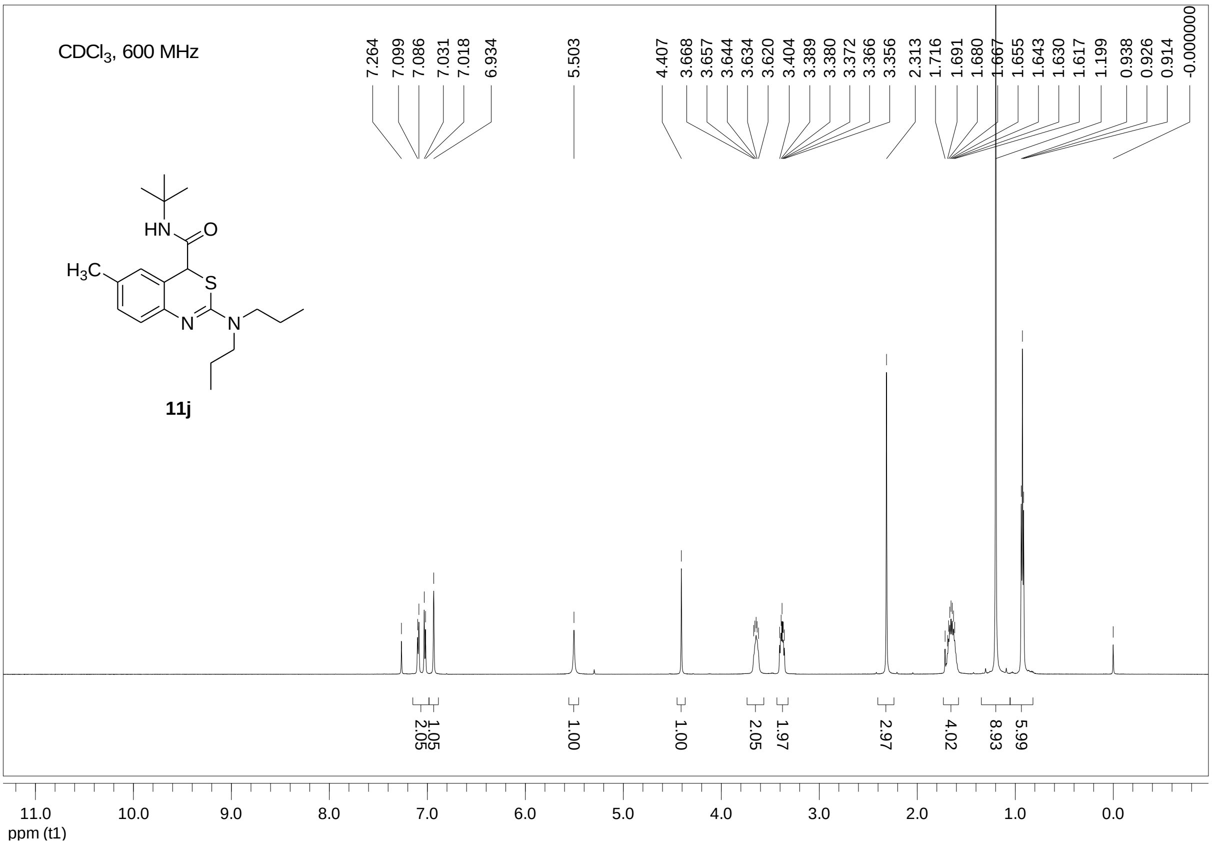
CDCl3, 600 MHz
7.264 7.099 7.086 7.031 7.018 6.934 5.503 4.407 3.668 3.657 3.644 3.634 3.620 3.404 3.389 3.380 3.372 3.366 3.356 2.313 1.716 1.691 1.680 1.667 1.655 1.643 1.630 1.617 1.199 0.938 0.926 0.914 -0.000000
HN
O
H3C
S
N
N
11j
2.05 1.05 1.00 1.00 2.05 1.97 2.97 4.02 8.93 5.99
11.0 10.0 9.0 8.0 7.0 6.0 5.0 4.0 3.0 2.0 1.0 0.0
ppm (t1)

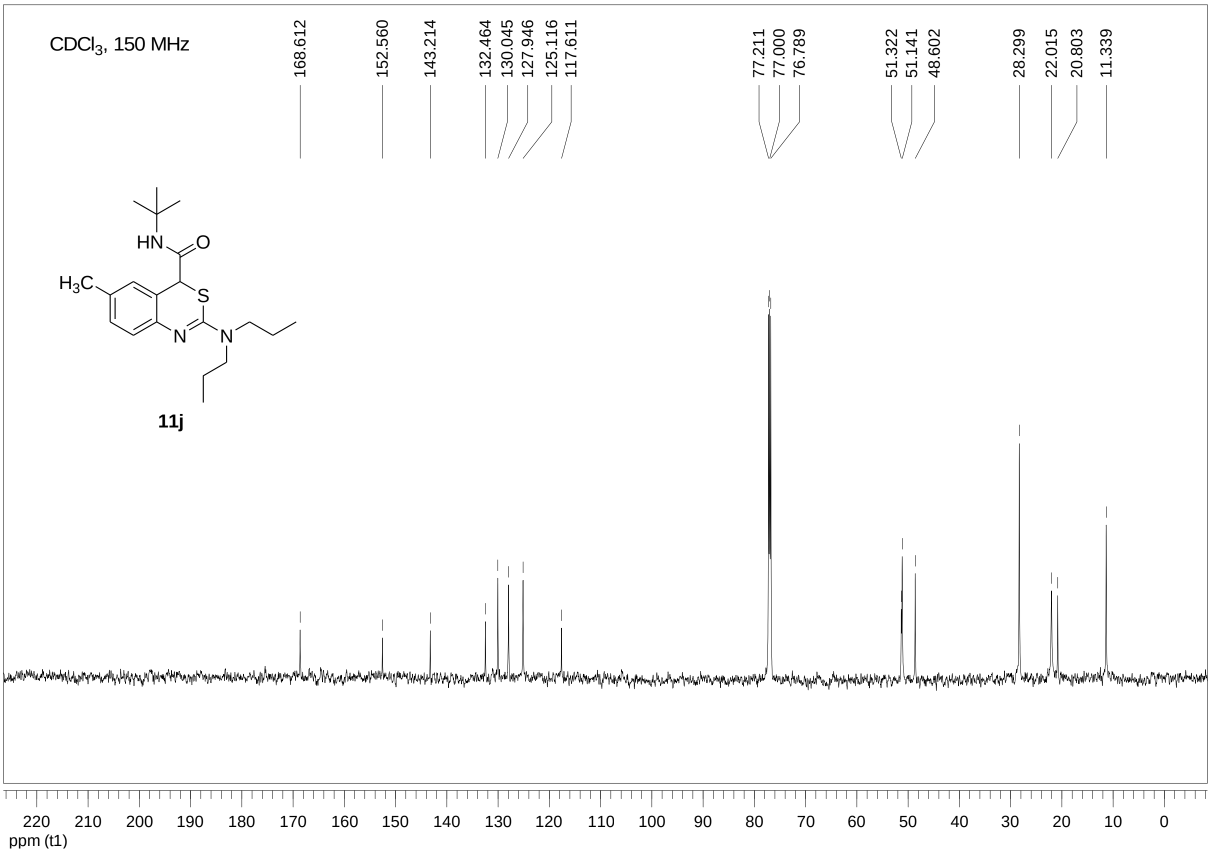
CDCl3, 150 MHz
168.612 152.560 143.214 132.464 130.045 127.946 125.116 117.611 77.211 77.000 76.789 51.322 51.141 48.602 28.299 22.015 20.803 11.339
HN
O
H3C
S
N
N
11j
220 210 200 190 180 170 160 150 140 130 120 110 100 90 80 70 60 50 40 30 20 10 0
ppm (t1)

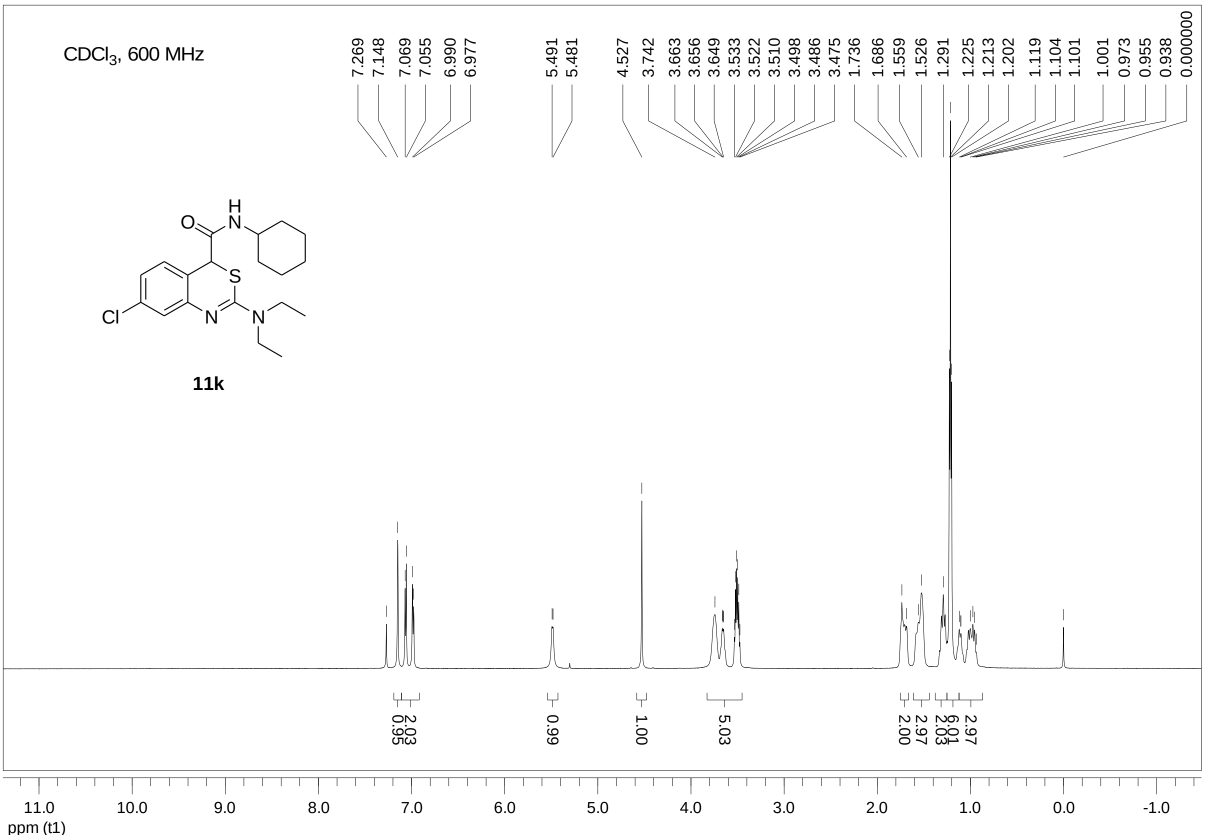

CDCl3, 600 MHz
7.269
7.148
7.069
7.055
6.990
6.977
5.491
5.481
4.527
3.742
3.663
3.656
3.649
3.533
3.522
3.510
3.498
3.486
3.475
1.736
1.686
1.559
1.526
1.291
1.225
1.213
1.202
1.119
1.104
1.101
1.001
0.973
0.955
0.938
0.000000
11k
0.95
2.03
0.99
1.00
5.03
2.00
2.97
2.03
6.01
2.97
ppm (t1)

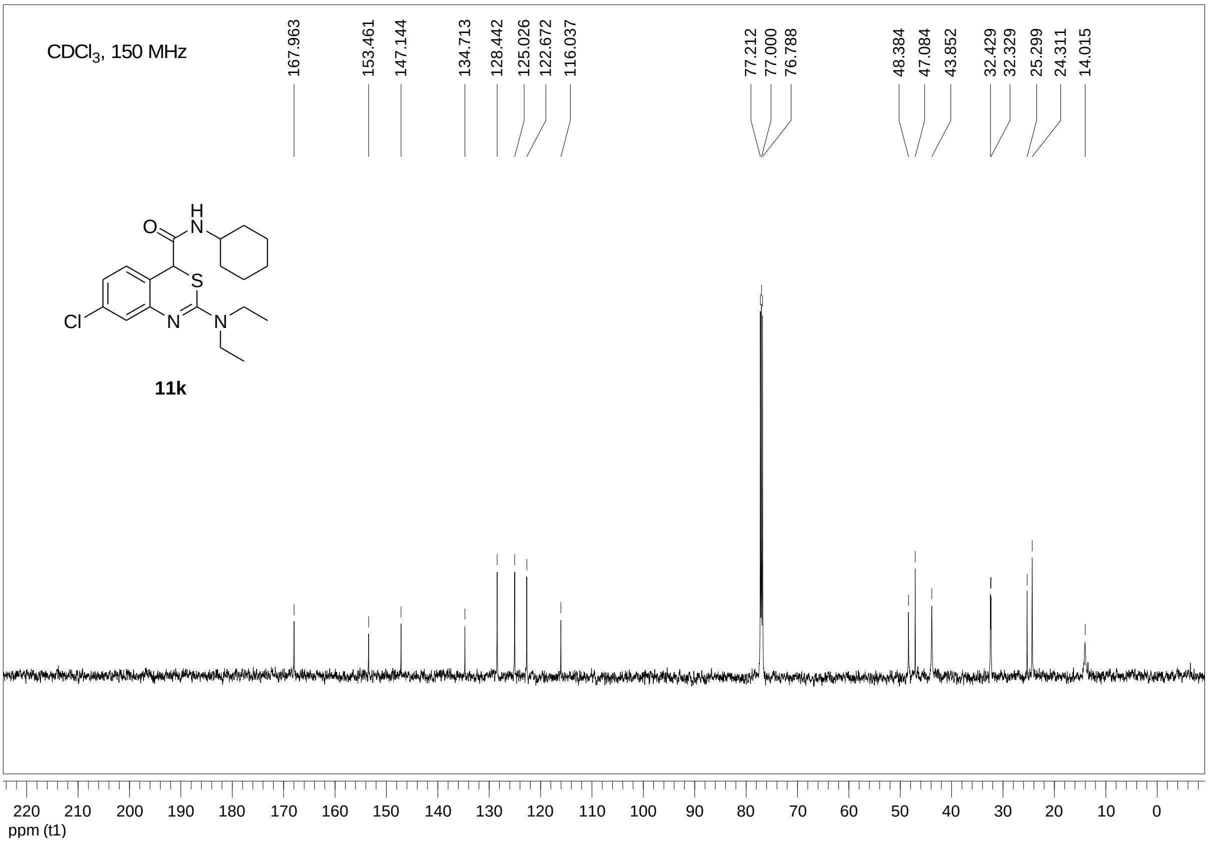

CDCl3, 150 MHz
167.963
153.461
147.144
134.713
128.442
125.026
122.672
116.037
77.212
77.000
76.788
48.384
47.084
43.852
32.429
32.329
25.299
24.311
14.015
11k
ppm (t1)

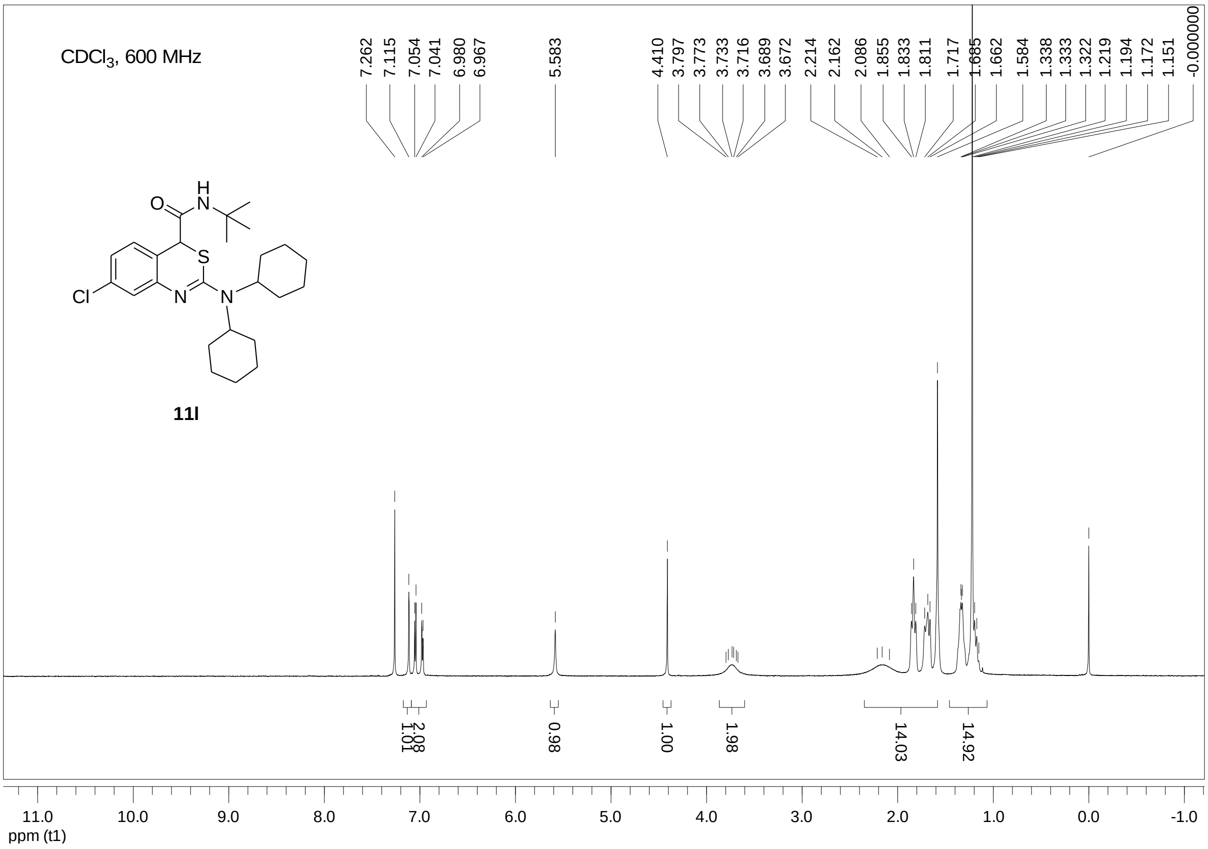
CDCl3, 600 MHz
7.262
7.115
7.054
7.041
6.980
6.967
5.583
4.410
3.797
3.773
3.733
3.716
3.689
3.672
2.214
2.162
2.086
1.855
1.833
1.811
1.717
1.685
1.662
1.584
1.338
1.333
1.322
1.219
1.194
1.172
1.151
-0.000000
11l
1.01
2.08
0.98
1.00
1.98
14.03
14.92
ppm (t1)

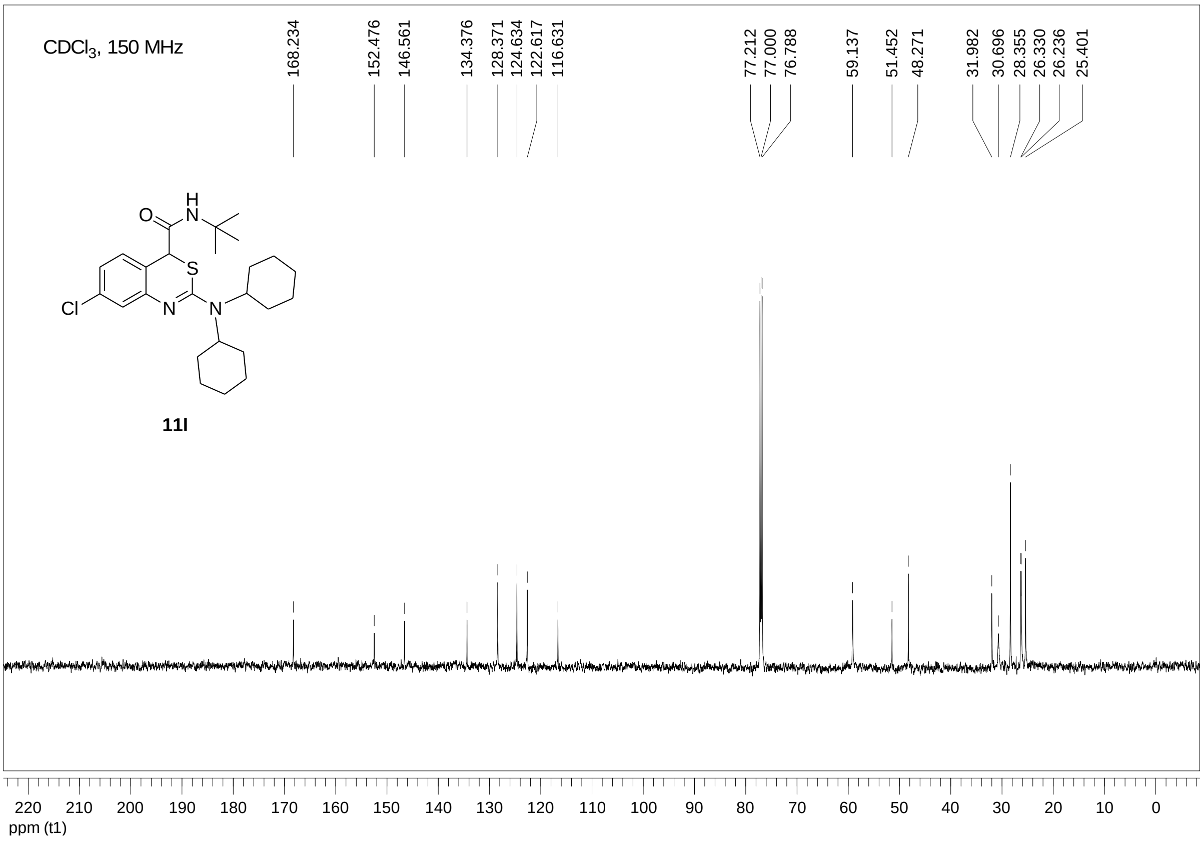
CDCl3, 150 MHz
168.234
152.476
146.561
134.376
128.371
124.634
122.617
116.631
77.212
77.000
76.788
59.137
51.452
48.271
31.982
30.696
28.355
26.330
26.236
25.401
11l
ppm (t1)

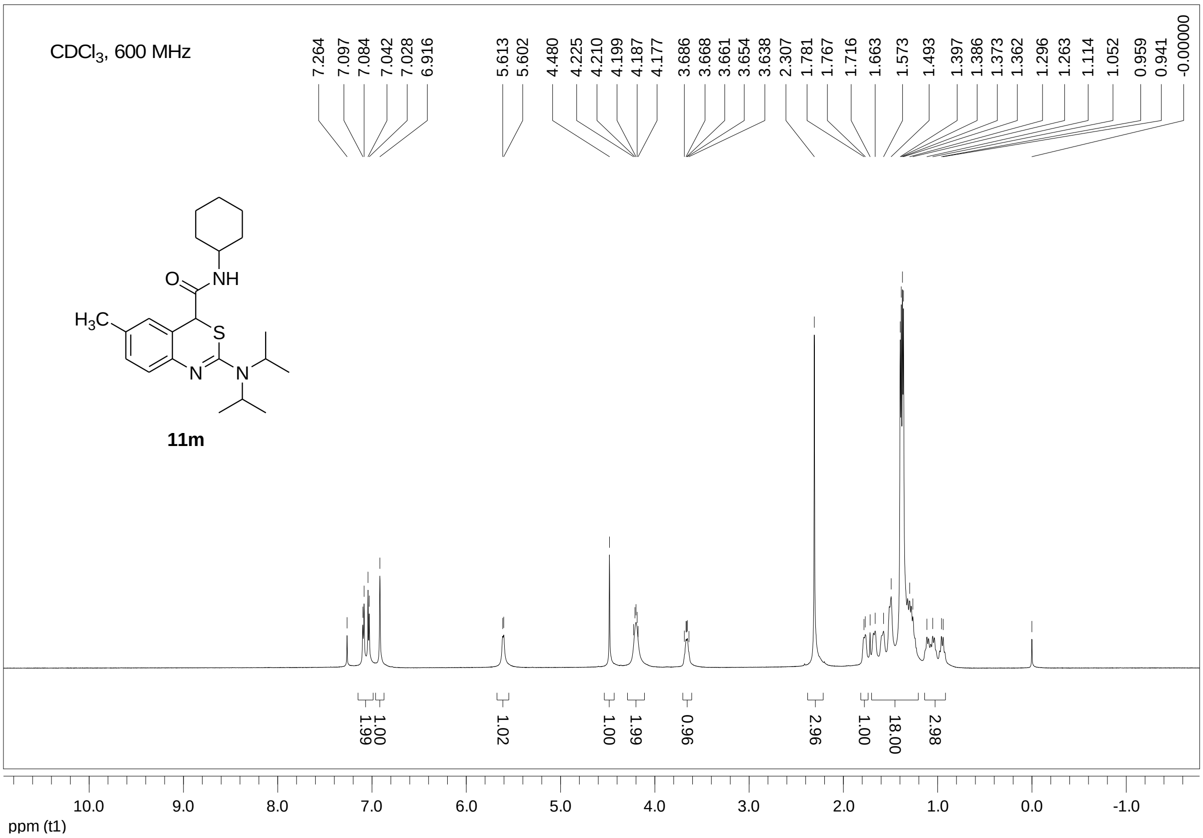
CDCl3, 600 MHz
7.264 7.097 7.084 7.042 7.028 6.916 5.613 5.602 4.480 4.225 4.210 4.199 4.187 4.177 3.686 3.668 3.661 3.654 3.638 2.307 1.781 1.767 1.716 1.663 1.573 1.493 1.397 1.386 1.373 1.362 1.296 1.263 1.114 1.052 0.959 0.941 -0.00000
O NH
H3C
S
N N
11m
1.99 1.00 1.02 1.00 1.99 0.96 2.96 1.00 18.00 2.98
10.0 9.0 8.0 7.0 6.0 5.0 4.0 3.0 2.0 1.0 0.0 -1.0
ppm (t1)

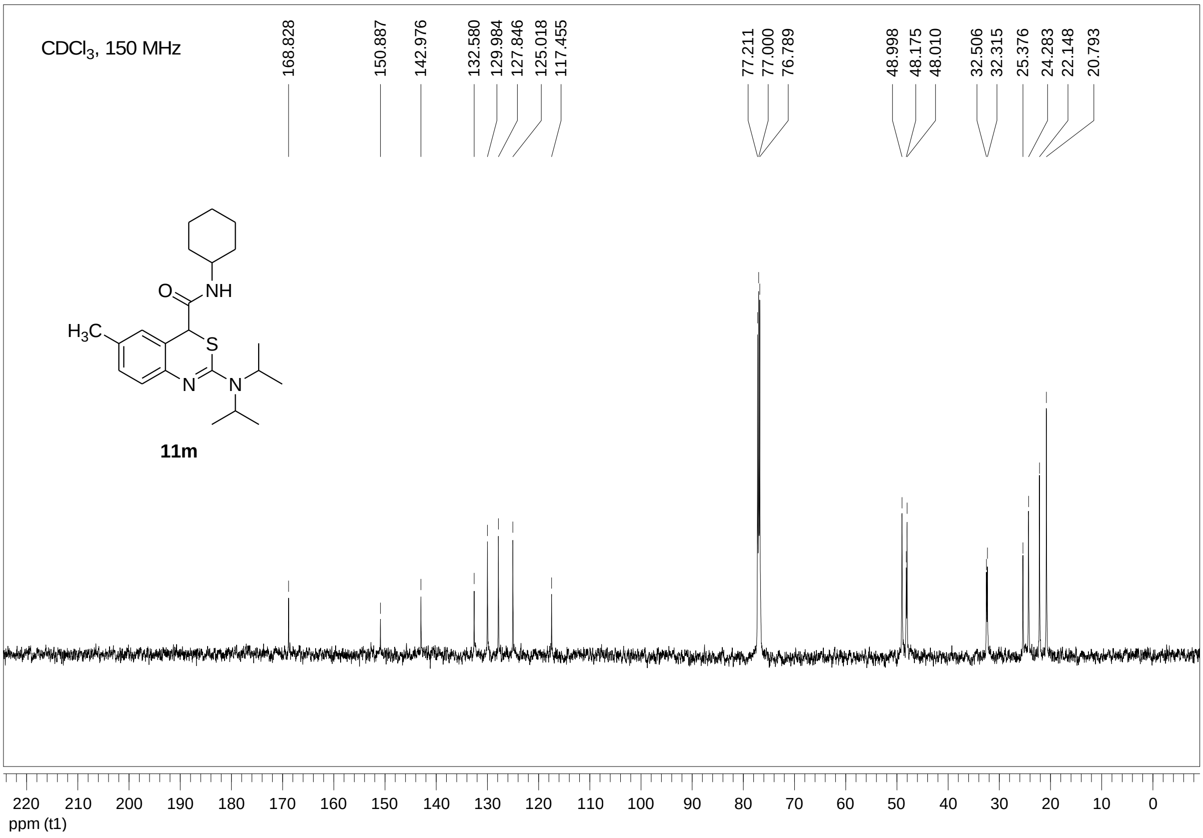
CDCl3, 150 MHz
168.828 150.887 142.976 132.580 129.984 127.846 125.018 117.455 77.211 77.000 76.789 48.998 48.175 48.010 32.506 32.315 25.376 24.283 22.148 20.793
O NH
H3C
S
N N
11m
220 210 200 190 180 170 160 150 140 130 120 110 100 90 80 70 60 50 40 30 20 10 0
ppm (t1)

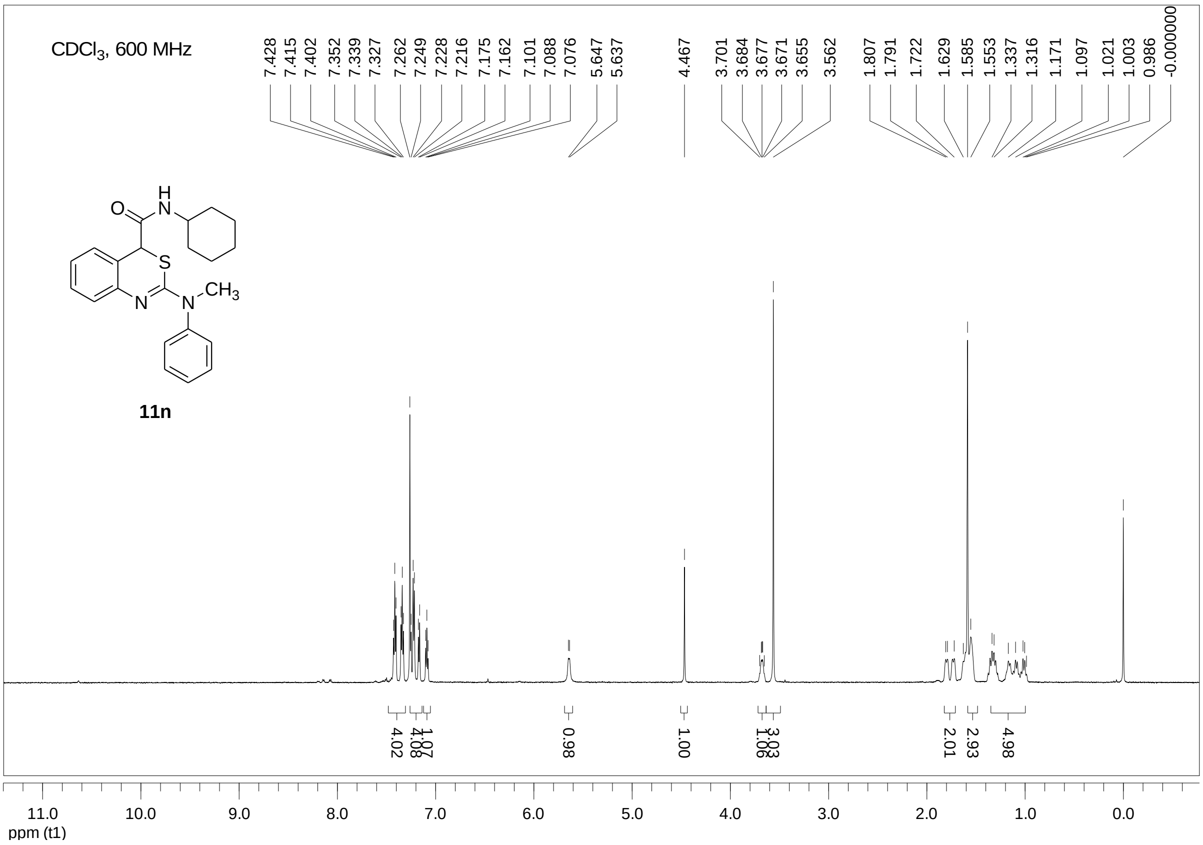
CDCl3, 600 MHz
7.428 7.415 7.402 7.352 7.339 7.327 7.262 7.249 7.228 7.216 7.175 7.162 7.101 7.088 7.076 5.647 5.637 4.467 3.701 3.684 3.677 3.671 3.655 3.562 1.807 1.791 1.722 1.629 1.585 1.553 1.337 1.316 1.171 1.097 1.021 1.003 0.986 -0.000000
O
H
N
S
N
N
CH3
11n
4.02 4.08 1.07 0.98 1.00 1.06 3.03 2.01 2.93 4.98
11.0 10.0 9.0 8.0 7.0 6.0 5.0 4.0 3.0 2.0 1.0 0.0
ppm (t1)

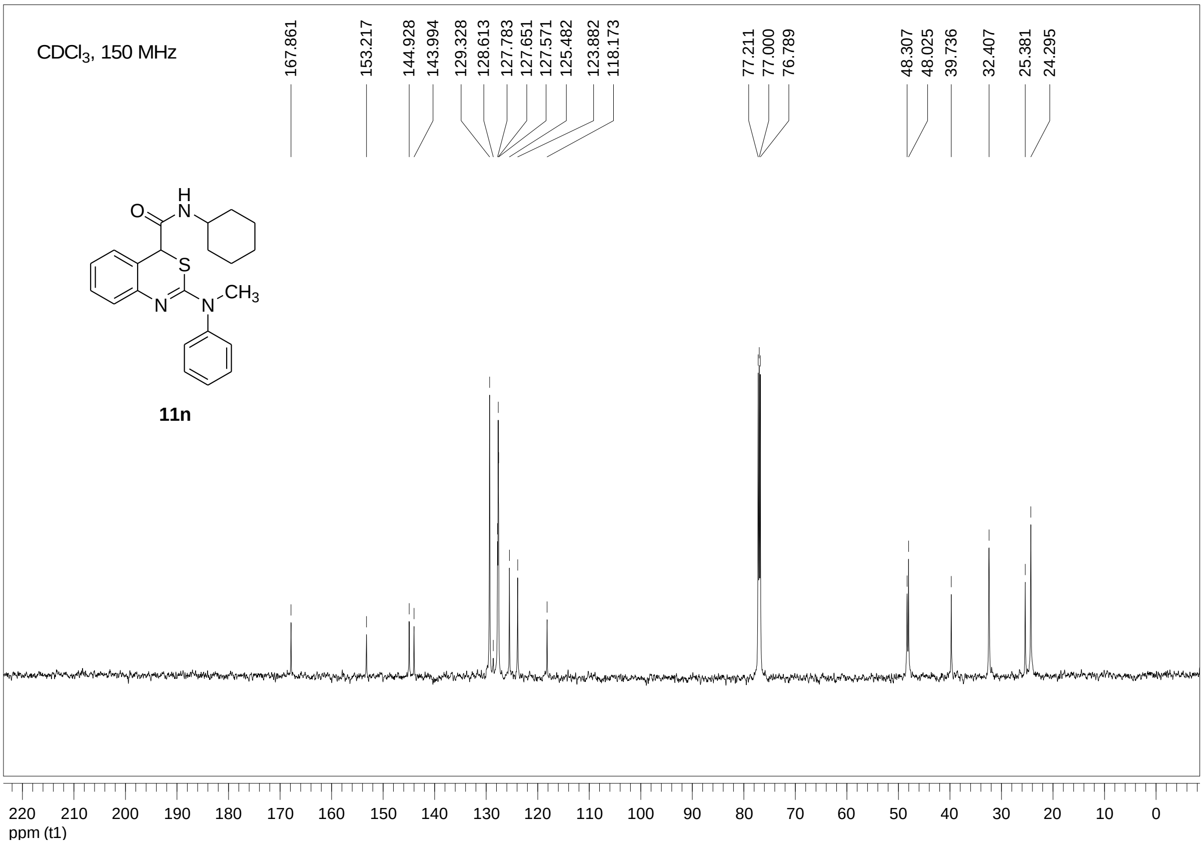
CDCl3, 150 MHz
167.861 153.217 144.928 143.994 129.328 128.613 127.783 127.651 127.571 125.482 123.882 118.173 77.211 77.000 76.789 48.307 48.025 39.736 32.407 25.381 24.295
O
H
N
S
N
N
CH3
11n
220 210 200 190 180 170 160 150 140 130 120 110 100 90 80 70 60 50 40 30 20 10 0
ppm (t1)

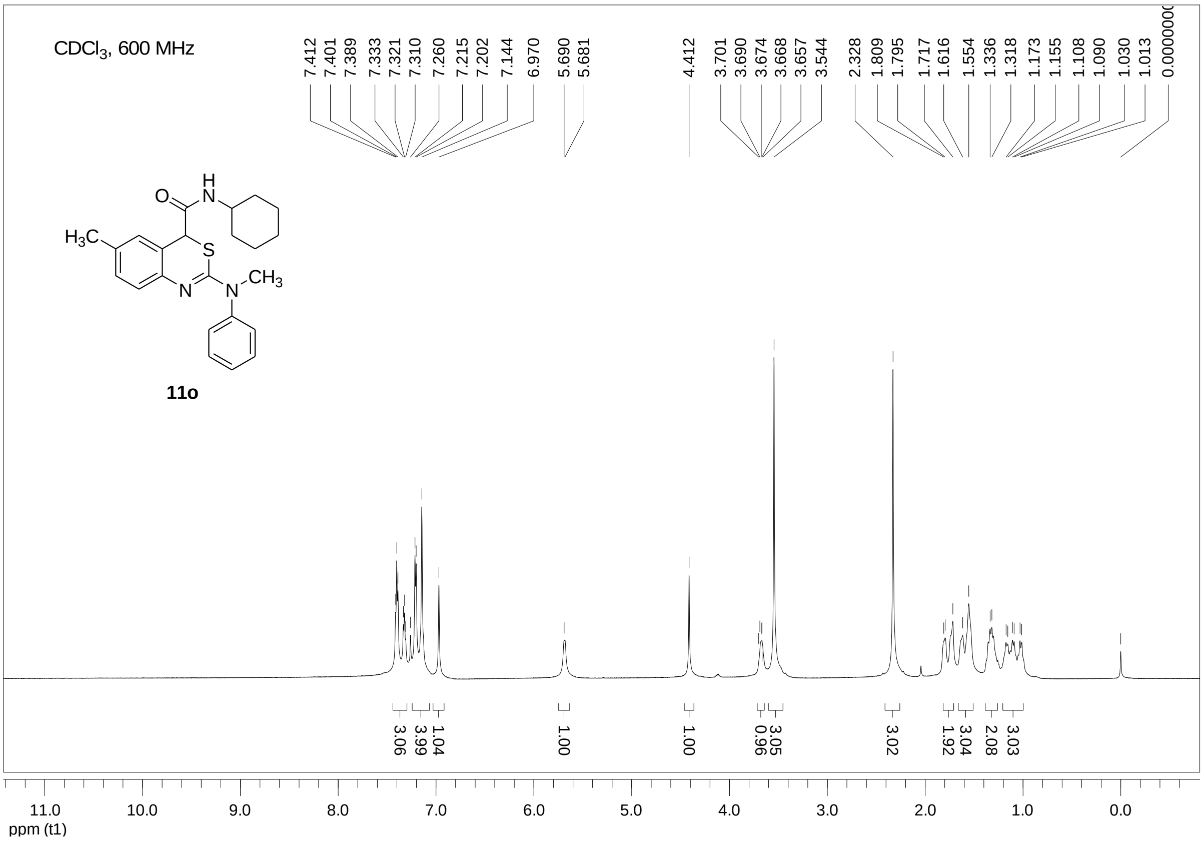
CDCl3, 600 MHz
7.412
7.401
7.389
7.333
7.321
7.310
7.260
7.215
7.202
7.144
6.970
5.690
5.681
4.412
3.701
3.690
3.674
3.668
3.657
3.544
2.328
1.809
1.795
1.717
1.616
1.554
1.336
1.318
1.173
1.155
1.108
1.090
1.030
1.013
0.0000000
11o
3.06
3.99
1.04
1.00
1.00
0.96
3.05
3.02
1.92
3.04
2.08
3.03
11.0
10.0
9.0
8.0
7.0
6.0
5.0
4.0
3.0
2.0
1.0
0.0
ppm (t1)

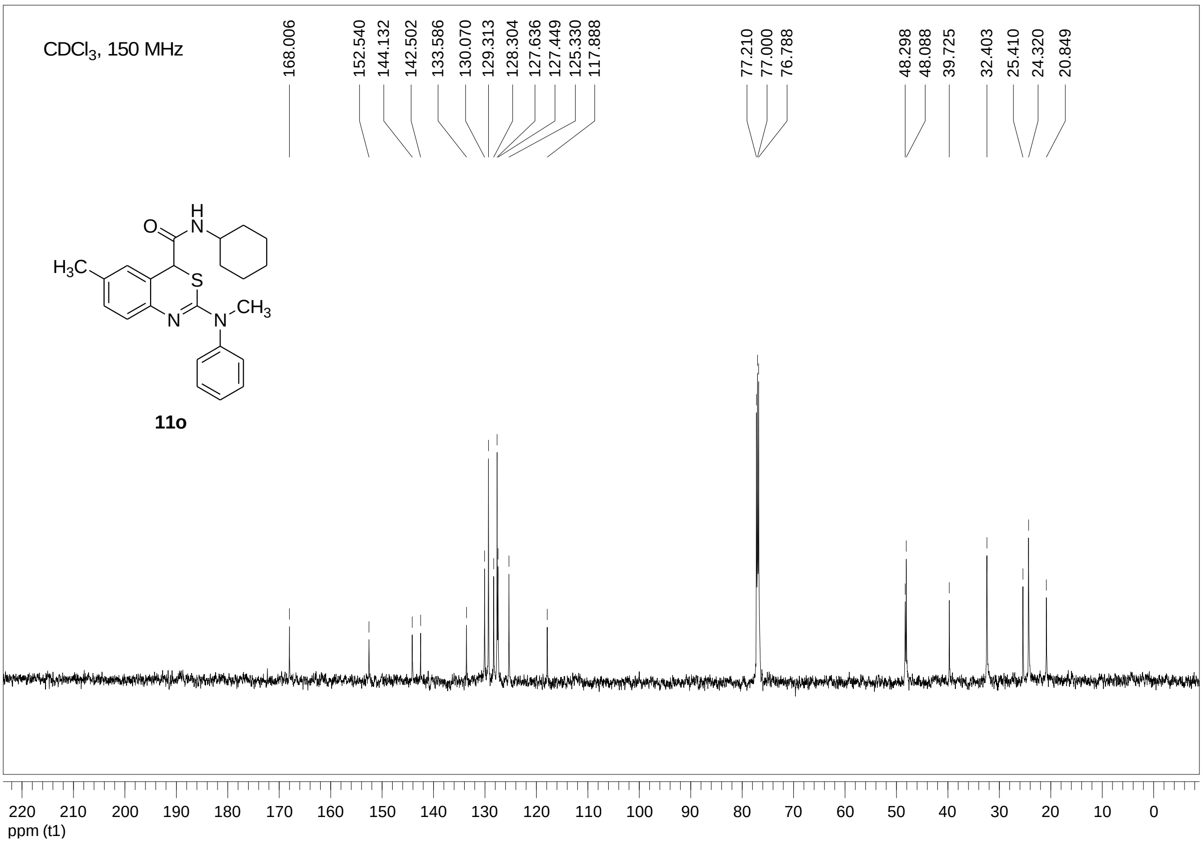
CDCl3, 150 MHz
168.006
152.540
144.132
142.502
133.586
130.070
129.313
128.304
127.636
127.449
125.330
117.888
77.210
77.000
76.788
48.298
48.088
39.725
32.403
25.410
24.320
20.849
11o
220
210
200
190
180
170
160
150
140
130
120
110
100
90
80
70
60
50
40
30
20
10
0
ppm (t1)
